# Exploring the role of modifiable sex/gender-specific risk and protective factors for anxiety among young people in high income countries: a systematic narrative review

**DOI:** 10.1186/s12889-026-26447-9

**Published:** 2026-02-18

**Authors:** Ciara Thomas, Obioha C Ukoumunne, Lucy Biddle, Myles-Jay Linton, Leah Attwell, Tamsin Ford, Judi Kidger

**Affiliations:** 1 University of Bristol, Bristol, UK; 2 NIHR Applied Research Collaboration South West Peninsula, University of Exeter, Exeter, UK; 3 University of Exeter, Exeter, UK; 4 University of Cambridge, Cambridge, UK

**Keywords:** Adolescence, Young people, Anxiety, Mental health, Gender differences

## Abstract

**Background:**

Anxiety is the most common mental health problem in young people and sex/gender differences have been consistently reported, with girls and young women experiencing twice the chance of anxiety compared to boys and young men. There is a limited understanding, however, of the underlying causes of these differences. This systematic review aims to synthesise research identifying modifiable sex/gender-specific risk and protective factors for anxiety among young people aged 16–24 in high income countries.

**Methods:**

A systematic literature search was conducted on 29th February 2024 and updated on 4th July 2025 across MEDLINE (Ovid), PsycINFO (Ovid), EMBASE (Ovid), Scopus, Sociological abstracts, and Web of Science. Observational studies reporting estimates of sex/gender-specific associations between modifiable risk and protective factors and anxiety according to DSM-5 categories were included. Results were summarised using narrative synthesis.

**Results:**

85 studies were included. Modifiable factors were grouped into levels: individual; interpersonal relationships; local community; and wider environment and society levels. The review identified conflicting results for sex/gender differences, demonstrating the methodological limitations of the evidence base and the complexity of the modifiable risk and protective factors implicated in the explanations for sex/differences in anxiety among young people aged 16–24 years. Potential sex/gender-specific risk factors emerged; early alcohol use initiation, parental overprotection and social media may be more anxiety-inducing in females than in males.

**Conclusions:**

This review indicates that sex/gender differences may exist in the associations between modifiable risk and protective factors and anxiety. Future longitudinal studies are crucial to understanding how these pathways differ by sex/gender. Studies are needed which explore whether sex/gender influences the relationship between anxiety and gender discrimination, peer relationships, school/college context, the workplace and the school-to-work transition. Such evidence has the potential to guide the development of effective sex/gender-specific mental health interventions.

**PROSPERO protocol registration:**

CRD42024518279.

**Clinical trial number:**

Not applicable.

**Supplementary Information:**

The online version contains supplementary material available at 10.1186/s12889-026-26447-9.

## Introduction

Anxiety is the most common mental health problem in young people [1, 2], and girls and young women experience higher rates of anxiety compared to boys [1, 3–6]. Sex/gender differences in the prevalence of common mental health problems, such as anxiety and depression, are well established in the literature [7]. Girls and young women are estimated to experience twice the chance of these mental health problems compared to boys and young men [8–12]. Anxiety symptoms are characterised by excessive worry, panic and fear or apprehension about real or perceived threats [1, 13]. While experiencing symptoms of anxiety is common and often situational with little impact on daily life, persisting symptoms can have a profound impact on everyday activities and future outcomes. There is evidence that anxiety first manifests in late childhood [14] and accelerates across mid-to-late adolescence, at around 16 years [15, 16]. Anxiety during adolescence predicts higher levels of anxiety in later life, academic underachievement, poorer adjustment in adulthood, lower life satisfaction, poor coping skills, and high chronic stress [5, 17, 18]. Identifying the modifiable sex/gender-specific risk and protective factors for anxiety will aid development of effective prevention and treatment interventions [19, 20].

Despite numerous studies reporting sex/gender differences in adolescent anxiety prevalence, there is limited understanding of the underlying causes, including psychological, biological, social and economic factors such as gender roles, expectations and inequality, familial factors, specific vulnerability factors and personal traits, academic pressures, body image, sexual violence, and peer relationships [21–25]. Some of these factors such as poverty, family relationships, school experience and neighbourhood environments [26], are more likely to be modifiable through population interventions. This review focussed on modifiable factors to ensure its findings have the potential to aid the development of UK-based gender-specific mental health interventions [26]. Furthermore, due to its intention to inform UK-based interventions, this review focusses on high income counties (HICs), as differences in cultural contexts and resource availability make findings from lower-middle income countries (LMICs) inapplicable to HICs [27].

While designing and conducting this review the research team sought to engage with young people to discuss terminology, priorities and interpretation of the findings. Involving young people through Patient and Public Involvement and Engagement (PPIE) in health research ensures that the voices of individuals affected by research outcomes are heard, enhancing the validity and impact of research by helping researchers understand young peoples’ unique priorities and perspectives [28].

Adolescent mental health problems are an important public health concern, with 1 in 7 adolescents aged 10–19 years estimated to experience a mental disorder [29] while 75% of mental health disorders have onset by the age of 24 [30]. Adolescence is a unique and formative time of life where new stressors such as puberty, identity formation, educational stress, and greater autonomy can increase vulnerability to poor mental health [29, 31–33]. It is a critical stage for psychosocial development [34]. The increasing importance of the peer group during adolescence increases the risk of peer stress and conflict, which can also compromise adolescent mental health [32, 35]. Adolescent mental health problems are associated with worse mental health in later life, a lower quality of life, poor social functioning, risk-taking behaviour, physical ill-health, and lower rates of educational attainment and employment [29, 31, 33, 36–38].

A systematic review was chosen as the most appropriate method because although previous articles have sought to summarise literature on sex/gender differences in internalizing symptoms and suicidal behaviour [1, 17, 20, 39–42], a systematic review identifying modifiable sex/gender-specific risk and protective factors for anxiety among young people aged 16–24 in HICs has not been conducted. This age group was chosen because it reports the highest rates of anxiety [8, 30]. Furthermore, it is during late adolescence that sex/gender differences in anxiety peak; by the age of 18 years, girls are more than twice as likely to experience internalising symptoms compared to boys [12]. Adolescence and young adulthood is a time of uncertainty where decisions about the future, for example deciding on school subjects and career paths, have been cited as a contributor to young people’s increase in mental health problems in recent years [43]. Adjusting to adult roles, completing secondary education, transitioning from school to work, enrolling in post-secondary education or vocational training, and living independently are all common life events during the age 16–24, which can also contribute to a mental health burden [44–47]. The 16–24 age range therefore represents a window of vulnerability where girls are more susceptible to developing anxiety [12, 48].

**Review question**:

What are the modifiable sex/gender-specific risk and protective factors for anxiety in young people aged 16–24 years in HICs?

## Methods

The systematic review was registered on PROSPERO (CRD42024518279). The review was conducted and reported following the Preferred Reporting Items for Systematic Reviews and Meta-Analysis (PRISMA) guidelines.

### Patient public involvement and engagement (PPIE)

Young People’s Advisory Group (YPAG) members from the National Institute for Health and Care Research Applied Research Collaboration (NIHR ARC West) were invited, on separate occasions, to discuss the methods and results of this review. This is in line with NIHR’s UK Standards for Public Involvement [49]. During the planning phase, a meeting was held with the YPAG to discuss potential methods of this review, including scope and terminology. The initial age group of interest for this review was 10 to 24 years and was based on definitions of adolescence and young people according to the World Health Organisation [50]. YPAG members considered this age range too broad, describing how different life is at age 10 compared to age 24 years and suggested a narrower age range. These discussions, plus revisiting the evidence regarding when rates of anxiety and gender differences in anxiety peak, resulted in the final age range of 16–24 years. YPAG members agreed with our initial plan of focussing the review on anxiety and considered this an important area where effective interventions do not always exist. During the session it was agreed that the most appropriate term used to describe the 16–24 age group was ‘young people’.

### Eligibility criteria

#### Inclusion criteria

This review included observational studies that provided extractable data on sex/gender-specific associations between modifiable risk and protective factors and anxiety. Peer-reviewed studies published in the English language from 2010 onwards were included, to ensure the review captured contemporary findings to inform intervention development. Studies were included if the mean age of participants lay between 16 and 24 years, if subgroup data were reported for participants aged 16–24 years, or if the majority of participants were aged 16–24 years. Studies with anxiety symptoms or disorders, in line with the Diagnostic and Statistical Manual of Mental Disorders (DSM-5) [25], as outcomes were included to ensure the review consistently focused on clinically meaningful anxiety symptoms and disorders. According to the fifth edition DSM-5, anxiety disorders include generalized anxiety disorder, panic disorder, specific phobias, agoraphobia, social anxiety disorder, separation anxiety disorder and selective mutism [51].

#### Exclusion criteria

Studies focusing solely on non-modifiable factors, such as genetic factors, were excluded. Studies that solely measured niche anxiety types, for example test anxiety and appearance anxiety, were excluded. As this review focuses on modifiable factors with a view to informing future interventions, excluding studies prior to 2010 ensures the social and environmental factors discussed are relevant to the life of young people today. This cut-off also reflects the steep upward trend in anxiety diagnosis and symptoms among young people in the UK; focussing on studies from this date onwards will aid efforts to explain underlying reasons for this trend [16, 52]. Studies with regression models including 3-way interactions (e.g. sex by discrimination by self-esteem in predicting anxiety) were included only if the interaction effect between discrimination and self-esteem on the outcome was reported separately for each sex/gender group. Finally, this review did not report the main effects for the risk factors from the included paper if the interactions between the risk factors and sex/gender group were included in the published regression model, because when an interaction term is included, the interpretation of the coefficients for the risk factor relates only to the reference category of the sex/gender variable with which it interacts.

### Identification of studies

A comprehensive literature search was conducted in MEDLINE (Ovid), PsycINFO (Ovid), EMBASE (Ovid), Scopus, Sociological abstracts, and Web of Science on 29th February 2024. The MeSH terms in the search strategy were translated for each database [see Additional file 1]. The strategy was finalised after consultation with a subject librarian and the YPAG to refine the search strategy and scope of the systematic review. Screening took place on CADIMA (version 2.2.4.2) [53]. After conducting piloting screening together, the primary reviewer (CT) independently screened 100% of the title/abstracts and for consistency the second reviewer (LA) double-screened 25% of the title/abstracts, which were selected randomly. CT and LA discussed and resolved conflicts together. The same process was done for full-text screening. Reference lists of included studies and relevant reviews were hand-searched to identify relevant studies missed by the search strategy. During full-text screening, corresponding authors were approached for the full text of inaccessible articles. The title-abstract screening consistency check had a concordance of 88%, and 100% concordance after discussion and conflict resolution. Full text screening had a concordance of 98% and then 100% after discussion. After completing a pilot extraction and quality assessment together, CT independently completed 100% of the data extractions and quality assessments, and for consistency, LA independently completed a set of ten data extractions and quality assessments. CT and LA discussed disagreements and appropriate amendments were made. The extraction form included: study characteristics (author, title, year of publication, country); population characteristics (sample size, age, gender, ethnicity, other demographic information, follow-up length, inclusion criteria, and subgroup); methodology (study design, study setting, aim, research question, hypothesis, analytical methods, theory/gender conceptualisation, exposure, exposure measure, exposure domain, outcome, outcome measure, outcome domain, and covariates); results (effect size, 95% confidence interval and p value/statistical significance); discussion (conclusions, strengths, limitations, future research suggestions), and quality rating [See Additional File 2]. Studies were critically appraised using the National Institute of Health (NIH) Quality Assessment Tool for Observational Cohort and Cross-Sectional Studies (14 items), with criteria developed by Tu et al. [54] for quantitative studies.

### Narrative synthesis

After the included studies were identified, CT assessed whether the studies met the criteria for meta-analysis. To meet the criteria, at least two studies had to: assess the same risk factor with the same measure; use the same anxiety disorder or symptom type with the same measure, and report the same measure of effect [55]. As none of the studies met the criteria it was not appropriate to perform meta-analysis. Therefore, a narrative synthesis was conducted to describe and summarise the characteristics and results of the included studies guided by Popay et al. [56]. To aid interpretation, risk and protective modifiable factors were grouped using a framework adapted from previous reviews’ categorisation schemes [26, 42, 57–59]: Modifiable factors were grouped into levels: individual; interpersonal relationships; local community; and wider environment and society levels. Risk and protective factors were organised into categories within each level.

## Results

### Study characteristics

**Fig. 1:**
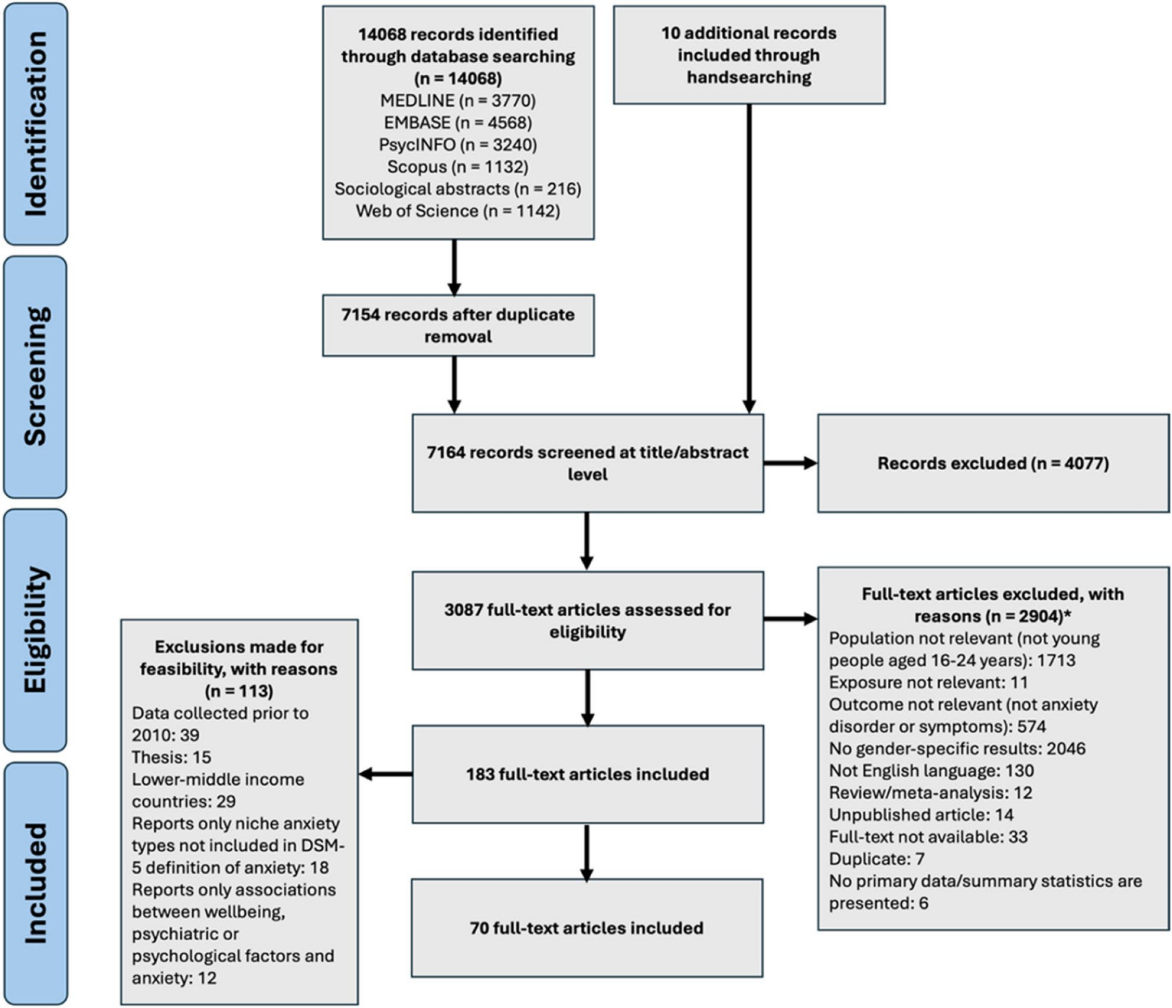
PRISMA diagram of included studies of systematic review covering up to 29th February 2024. *Exclusion reason numbers are generated from the CADIMA systematic reviewscreening software and do not add up to the number of articles excluded due toarticles having multiple exclusion reasons [53]

**Fig. 2:**
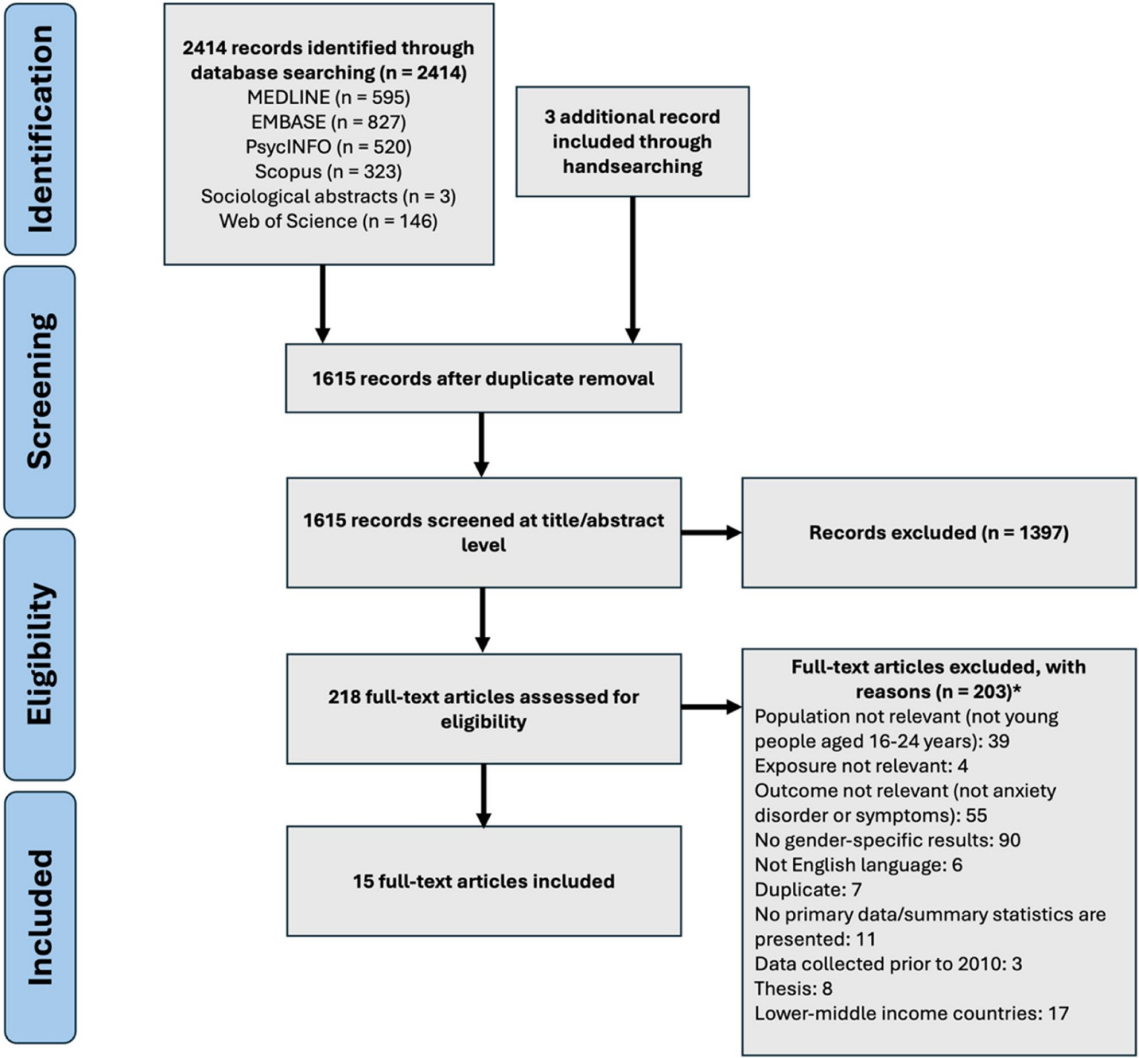
PRISMA diagram of included studies from update search on 4th July 2025. *Exclusion reason numbers are generated from the CADIMA systematic review screening software and do not add up to the number of articles excluded due to articles having multiple exclusion reasons [53]

In total, 7164 papers were identified through database searching and other sources, with 4077 excluded after title and abstract screening (Fig. 1). Of the 3087 full-texts screened, 12 were obtained after contacting authors who provided access to the full texts that were inaccessible to the review team during screening. Of the 3087 papers screened during full-text screening, 183 met inclusion criteria. A series of additional exclusions were made to focus the review. For this systematic review to inform the development of a UK-based mental health intervention, studies conducted in LMICs were excluded (*n* = 29). To further prioritise contemporary findings, studies that collected data before 2010 were excluded (*n* = 39); these studies were initially included as they were published after 2010. Unpublished papers were also excluded; lack of peer-review introduces a risk of bias due to lower methodological quality (*n* = 15). As the comorbidity of mental health problems is well documented [60–63], studies only reporting relationships between anxiety and psychiatric and related factors, such as depression, quality of life, personality, wellbeing, were excluded (*n* = 12). These decisions were made after meetings with an academic advisory board and with the YPAG. An update search was conducted on 4th July 2025, yielding 15 additional studies to be included in this review (Fig. 2). This selection process yielded a final number of 85 articles.

The studies included in this review explored symptoms of anxiety (*n* = 66), social anxiety (*n* = 13), generalised anxiety (*n* = 10), worry (*n* = 2), and panic (*n* = 1). The studies applied 37 different measures of anxiety. The mean age of participants ranged from 16 to 24 years. The total number of participants ranged from 34 [64] to 609,381 [65]. Seventy-four studies had a mixed sex/gender group of participants, and ten studies were single sex/gender [66–75]. The mixed sex/gender studies were mostly sex/gender balanced, although 30 studies comprised more than 60% girls/women/females [64, 68, 76–105] and two studies had more than 60% boys/men/males [104, 105]. The majority of studies were conducted in the USA (*n* = 42), with others from Canada (*n* = 8), Australia (*n* = 5), Spain (*n* = 4), Italy (*n* = 5), Norway (*n* = 3), Sweden (*n* = 3), Denmark (*n* = 2), Finland (*n* = 2), Greece (*n* = 2), plus the Netherlands, Taiwan, France, Hong Kong, Ireland, South Korea, Slovakia, Sweden, and Austria (all *n* = 1). The ethnicity of participants was not reported in 40 studies [65, 72, 76, 77, 80, 82, 84, 85, 87, 96, 98–100, 106–129]. A total of 15 studies predominantly consisted of individuals from ethnic minority groups [68, 70, 73, 75, 79, 89, 91, 92, 97, 103, 104, 130–133]. The remaining studies had a predominantly Caucasian population; 28 consisted of more than 60% Caucasian participants [66, 67, 69, 71, 74, 78, 81, 88, 90, 93–95, 101, 102, 105, 134–146]. Socioeconomic information was reported in 27 studies [65, 68, 69, 73, 79, 88, 90–95, 99, 107, 109, 113, 115, 120, 125, 127, 134, 136, 138, 141, 145–147] and participants were mostly from middle-class socio-economic status backgrounds. A summary of the included studies is presented in Additional file 3, Appendix 1.

### Sex and gender terminology of included studies

Included studies often used ‘sex’ and ‘gender’ terminology interchangeably. Sex is defined as a binary biological attribute i.e. ‘male or female’ [148], whereas ‘gender’ refers to socially constructed norms, roles and expectations of women, men, girls and boys [60, 149, 150]. The World Health Organisation defines gender as “someone’s personal and deeply felt internal sense of the self, which may or may not correspond with the person’s physiology or designated sex at birth” [150]. The terms ‘male’ and ‘female’ are sex-specific terminology and ‘woman’, ‘man’, ‘girl’, and ‘boy’ are gender-specific [151], but only 15 studies followed this terminology consistently [85, 86, 96, 103, 109, 116, 118, 125, 126, 132, 135, 139, 141, 142, 145], of which, seven explored ‘sex’ differences and used the terms ‘male and females’ [86, 109, 118, 135, 139, 141, 145], and eight explored ‘gender’ differences and used the terms ‘woman’, ‘man’, ‘girl’, and ‘boy’ [85, 96, 103, 116, 125, 126, 132, 142]. The remaining studies used these terms interchangeably, without differentiating between sex and gender [151].

Regarding sex/gender analyses, 42 studies used the term ‘gender’ but also the binary sex-specific ‘male and female’ approach to analyses and gave no detail on how gender was ascertained [76, 77, 79, 81, 82, 84, 87, 88, 90, 91, 93–98, 100, 101, 104–106, 110, 114–117, 121, 126–133, 136–138, 140, 142, 143, 152]. Four studies used the term ‘gender’ despite using registry information that indicated biological sex to conduct analyses [65, 107, 112, 120]. Eleven studies asked for participants’ gender [64, 80, 83, 85, 92, 99, 102, 122, 123, 125, 146], but five of these conducted analyses based on biological sex [80, 99, 102, 123, 146]. Of the ten single-sex/gender studies [66–75], only four were explicit about sex or gender-related inclusion criteria; three included participants assigned female at birth [72–74] and another included participants who identified as cisgender males [67]. The remaining studies explored ‘sex’ differences and used the binary sex-specific ‘male and female’ approach to analyses [78, 80, 86, 89, 108, 109, 111, 113, 118, 119, 123, 124, 134, 135, 139, 141, 144, 145, 147].

For consistency, this review uses the term sex/gender due to the entanglement of sex and gender in previous studies [153]. This review uses the same sex-specific or gender-specific terminology used by the authors; when studies use only gender-specific terminology this review does the same. However, when studies use sex and gender-specific terminology interchangeably this review uses sex-specific terminology ‘male’ and ‘female’.

### Quality assessment

The studies included in this review all had a well-defined research question, study population, eligibility criteria and outcome measurement. Nine studies conducted a sample size power calculation [64, 73, 80, 90, 98, 100, 106, 109, 136]. Sixty-two studies were cross-sectional studies and did not meet the following NIH quality assessment criteria: exposures measured prior to the outcome; a sufficient timeframe, and exposures being assessed more than once over time. One study blinded outcome assessors to participants’ exposure status [144]. Seven studies did not examine different levels of exposure and instead had dichotomous exposure variables [65, 70, 75, 120, 128, 129, 146]. All variables were clearly defined, but twelve studies developed their own non-validated exposure [75, 87, 99, 102, 111, 119, 120, 125, 126, 145, 146] and outcome [112] variables. A summary of the quality assessment is presented in Additional file 3, Appendix 2–3.

### Certainty of evidence

A Grading of Recommendation, Assessment, Development and Evaluation (GRADE) assessment was conducted following Murad et al.’s [72] guidance on using GRADE to rate the certainty of evidence when a narrative summary is conducted instead of meta-analysis. The overall rating of the certainty was ‘very low’ due to serious risk of bias, inconsistency and imprecision. A summary of the GRADE assessment is presented in Additional file 3, Appendix 4.

## Individual level modifiable factors

Seven factors fell under this category: individual negative life events and family adversity; self-esteem; physical health and health behaviours; sociodemographic factors; social media, and religion. A simplified summary of the sex/gender-specific results is presented in Additional file 3, Appendix 5.

### Individual negative life events and family adversity

The review identified ten cross-sectional [75, 78, 81, 82, 88, 95, 99, 101, 104, 125] and six longitudinal studies [93, 107, 115, 120, 129, 141] that explored the association between anxiety and negative life events and family adversity.

#### Sexual abuse

Six cross-sectional studies [75, 81, 82, 88, 99, 104] explored the association between sexual abuse and anxiety. Duncan et al. [81] found evidence of sex/gender moderation (interaction *p* = 0.006), such that the association between sexual harassment and social anxiety symptoms was significant for females aged 18 (*p* = 0.013) and 16 years (*p* = 0.001) and males aged 18 (*p* = 0.002) and 20 years (*p* = 0.001), but not females aged 20 years and males aged 16 years. Carlberg et al. [99] found evidence of sex moderation (interaction: *p* < 0.001), such that unwanted online sexual solicitation (requests to engage in sexual activities, sexual talk, to give personal sexual information, or to meet offline) was a greater risk factor for anxiety in males than females (no p-values reported). Of the three studies reporting sex/gender stratified results only [82, 88, 104], two reported a sex/gender difference [82, 88]. Maciel et al. [88] found that child sexual abuse, where victims had family ties with the perpetrator, was a risk factor of anxiety in females (correlation coefficient = 0.33, *p* < 0.05) but not in males (correlation coefficient = -0.01, *p* < 0.05). Reed et al.’s [75] female-only study found evidence that cyber sexual harassment was a risk factor for anxiety (*p* < 0.001). Gasso et al. [82] found that receiving a sext (sexual text messages, nude images, and/or sexual content) was a risk factor for anxiety in females (OR = 1.53, 95% CI: 1.18 to 1.99, *p* = 0.001) but not in males (OR = 0.97, 95% CI: 0.62 to 1.54, *p* = 0.907). Being a victim of nonconsensual dissemination was also positively associated with anxiety in females (OR = 2.20, 95% CI: 1.02 to 4.72, *p* = 0.038;) but not in males (OR = 2.36, 95% CI: 0.60 to 9.30, *p* = 0.206). Being pressured to sext was positively associated with anxiety in females (OR = 1.55, 95% CI: 1.19 to 2.02, *p* = 0.001) but not in males (OR = 1.37, 95% CI: 0.79 to 2.37, *p* = 0.256). Being threatened to sext was positively associated with anxiety in females (OR = 2.12, 95% CI: 1.10, 4.09, *p* = 0.02) but not in males (OR = 1.95, 95% CI: 0.18 to 21.73, *p* = 0.580). ‘Any sexting behaviours’ was also positively associated with anxiety in females (OR = 1.45, 95% CI: 1.09 to 1.92, p = 0.010) but not in males (OR: 1.55, 95% CI: 0.95 to 2.53, p = 0.075).

#### Child maltreatment and parent abuse

One longitudinal [115] and one cross-sectional study [104] explored the association between anxiety and child maltreatment and parent abuse, and reported sex/gender-stratified results. Only Davila et al. [104] identified a sex/gender difference, such that a high level of childhood maltreatment was associated with higher levels of anxiety in males (*p* < 0.01), but not in females (p-value not reported).

#### Family mental health problems

Two cross-sectional studies [78, 95] and one longitudinal study [107] explored the association between family mental health and anxiety. One study explored whether sex/gender moderated these associations [107] and two studies reported sex/gender-stratified results only [78, 95]. Berenz et al. [78] identified sex/gender differences; lifetime traumatic events and family history of mental health problems were risk factors for anxiety in females (standardised regression coefficient = 0.18, 95% confidence interval (CI): 0.08 to 0.27, *p* < 0.001; standardised regression coefficient = 0.21, 95% CI: 0.07 to 0.35, *p* = 0.002, respectively) but not in males (standardised regression coefficient = 0.18, 95% CI: -0.15 to 0.46, *p* = 0.24; standardised regression coefficient = 0.02, 95% CI: -0.25 to 0.28, *p* = 0.9, respectively). Stearns et al. [95] found that perceived maternal and paternal anxiety were risk factors for anxiety in males and females (all *p* < 0.01).

#### ‘Adverse life events’

Durham et al. [101] found evidence of a 3-way interaction between sex, negative life events **a**nd the ‘Difficulty with goal-directed behaviour aspect’ of the emotional regulation scale in predicting anxiety. Sex moderated the interaction between negative life events and ‘Difficulty with goal-directed behaviour’ in predicting anxiety (interaction: *p* = 0.025), such that for males, there was a positive association between negative life events and trait anxiety for those who have more difficulty engaging in goal-directed behaviour, but a slightly negative relationship for those with less difficulty engaging in goal-directed behaviour. For females there was a positive association between negative life events and trait anxiety regardless of difficulty engaging in goal-directed behaviour.

### Self-esteem and related factors

Ten cross-sectional studies [66, 73, 77, 81, 90, 92, 96, 110, 136, 137] and one longitudinal study [79] explored associations between anxiety and self-esteem and related factors. Four studies explored whether sex/gender moderated these associations [79, 92, 110, 136], six reported sex/gender-stratified results [66, 77, 81, 90, 96, 137] and one was single-sex [73]. Of these, one study found evidence of a three-way interaction between sex/gender, self-compassion and age (interaction *p* = 0.008), such that for those aged 17–19 years, self-compassion was more protective against anxiety in males compared to females (*p* = 0.013) [136]. Di Blasi et al. [137] identified sex/gender differences, such that subscales of the Offer self-Image questionnaire were associated with anxiety in males and females differently: Positive perceptions of family relationships (family relationships subscale) was a statistically significant risk factor for social anxiety (Social Interaction Anxiety Scale) in females (OR = 1.070, 95% CI: 1.02 to 1.12, *p* = 0.007), but not in males (OR = 1.057, 95% CI: 0.99 to 1.12, *p* = 0.065), and higher levels of External Mastery (a state of adjustment between the subject and his or her environment that is favourable to emotional growth) was protective against social anxiety in females (OR = 0.92, 95% CI: 0.87 to 0.97, *p* = 0.003) but not in males (OR = 0.985, 95% CI: 0.92 to 1.05, *p* = 0.649). Higher levels of Emotional Tone (consistency and stability of emotions) were protective against social anxiety in males (OR = 0.883, 95% CI: 0.82 to 0.95, *p* = 0.001), but not in females (OR = 0.999, 95% CI: 0.94 to 1.06, *p* = 0.968). In the male-only study, self-esteem and self-compassion were protective factors against anxiety (both *p* < 0.001) [66].

### Physical health and health behaviours

Five longitudinal [69, 108, 113, 124, 147] and 19 cross-sectional [64, 73, 74, 76, 78, 80, 83–85, 97, 104, 105, 118, 119, 127, 135, 138, 145, 146] studies explored the relationship between anxiety and physical health or health behaviours.

#### Alcohol and drug use

Three longitudinal [113, 124, 147] and ten cross-sectional studies [73, 74, 78, 80, 83, 104, 105, 119, 127, 135] explored the association between anxiety and alcohol or drug use.

Of the five studies exploring sex/gender moderation [80, 83, 113, 119, 127], three studies found evidence of sex/gender moderating the association between alcohol and drug use and anxiety [83, 119, 127]. Johannessen et al. [119] found evidence of sex/gender moderation (interaction *p* = 0.01), such that early onset of alcohol consumption was more strongly related to anxiety in females (no p-values reported) than males [119]. Similarly, Hellemans et al. [83] found evidence of sex/gender moderation (interaction *p* < 0.01), such that the association between problematic cannabis use and anxiety was stronger for females (*p* < 0.001), compared to males (*p* < 0.05). Skogen et al. [127] found evidence of sex/gender moderation (interaction *p* = 0.021), such that there was an association between illicit drug use and anxiety in males (odds ratio (OR) = 2.44, 95% CI: 1.57 to 3.78, p-value not reported) but not in females (OR = 1.30, 95% CI 0.98 to 1.73, p-value not reported). Sex/gender moderated the association between excessive alcohol consumption and anxiety (interaction *p* < 0.001), such that the there was an association in males (OR = 4.36, 95% CI: 2.52 to 7.57) but not in females (OR = 1.23, 95% CI: 0.87 to 1.73) [127].

Of the six studies reporting sex/gender-stratified results only [78, 104, 105, 124, 135, 147], three identified sex/gender differences [78, 124, 135]. Atkinson et al. [135] reported a statistically significant sex/gender difference (test for sex/gender difference *p* < 0.05), such that the association between lifetime alcohol problems and anxiety was slightly stronger in males (correlation coefficient = 0.379, *p* < 0.0001) than in females (correlation coefficient = 0.249, *p* < 0.0001). Reiselbach et al. [124] also reported a statistically significant sex/gender difference (test for sex/gender difference *p* < 0.05), such that substance use was a protective factor against anxiety in females (standardised regression coefficient = − 0.15, 95% CI: −0.22 to − 0.08, *p* < 0.01), but not in males (standardised regression coefficient = − 0.02, 95% CI: −0.09 to 0.05, *p* = 0.54). Berenz et al. [78] found an association between anxiety and a younger age of alcohol use initiation in females (standardised regression coefficient = -0.15, 95% CI: -0.29 to -0.02, *p* = 0.03), but not in males (standardised regression coefficient = -0.11, 95% CI: -0.28 to 0.07, *p* = 0.22). Buckner et al. [74] included participants assigned female at birth and found evidence that alcohol-related problems, and drinking to cope, conform, be social or enhance enjoyment were risk factors for anxiety (*p* < 0.01). Tao et al. [73] included participants assigned female at birth who self-identified as Asian, Black, Hispanic or Latina and found evidence that substance use as coping was a risk factor for anxiety (*p* < 0.001).

#### Physical activity

This review identified one longitudinal [108] and four cross-sectional studies investigating the association between physical activity and anxiety [84, 85, 128, 145]. Herring et al. [84] identified a sex/gender difference, such that physical activity was a protective factor for generalised anxiety disorder in males (standardised regression coefficient = -0.708, 95% CI: -1.074 to -0.341, *p* < 0.001), but not females (standardised regression coefficient = -0.058, 95% CI: -0.909 to 0.793, *p* = 0.894) [84]; physical activity, however, was a protective factor for social anxiety disorder in girls (standardised regression coefficient = -0.689, 95% CI: -1.078 to 0.300, *p* = 0.001) but not boys (standardised regression coefficient = -0.226, 95% CI: -1.117 to 0.665, *p* = 0.619) [84]. Wise et al. [128] found no evidence of sex/gender moderating the negative association between returning to sport after Covid-19 restrictions and anxiety [128]. Giannotta et al. [108] found no evidence that the associations between anxiety and vigorous physical activity were different between girls and boys (non-significant p-value, not reported).

#### Physical health condition

Five cross-sectional studies investigated the association between physical health conditions and anxiety [64, 76, 97, 118, 146]. Ferro et al. [118] found no evidence of sex/gender moderating the positive association between chronic health conditions and anxiety. Of the four studies reporting sex/gender stratified results [64, 76, 97], one identified a sex/gender difference, such that pain intensity was a risk factor for worry in females (*p* < 0.001) but not in males (*p*-value not reported) [97].

#### Body dissatisfaction

Two studies explored the relationship between body dissatisfaction and anxiety symptoms [69, 76]. One cross-sectional study identified no sex/gender differences [76] and one women-only longitudinal study found evidence of positive associations between anxiety and body surveillance and body dissatisfaction in both Caucasian (*p* < 0.001) and African American (*p* < 0.001) young women [69]. No association was found between BMI and anxiety, except in African American young women at baseline only (correlation coefficient = 0.26, *p* < 0.05).

### Socioeconomic status

Two longitudinal [120, 139] and five cross-sectional [65, 70, 73, 91, 97] studies explored the association between socioeconomic status (SES) and anxiety. Neblett et al. [91] found evidence of sex/gender moderation (interaction *p* = 0.028), such that young men from lower SES backgrounds (*p* < 0.001) and young women from higher SES backgrounds (*p* = 0.002) were at greater risk of anxiety associated with racial discrimination. Of the four studies reporting sex/gender-stratified results [65, 97, 120, 139], two identified potential sex/gender differences. Zvolensky et al. [97] found that a higher subjective social status was a protective factor against worry in females (*p* < 0.05) but not in males (*p*-value not reported). Mar et al. [65] found that compared to medium-to-high SES females, medium-to-high SES males had a lower likelihood of anxiety (OR: 0.52, 95% CI: 0.50 to 0.54, *p* < 0.01), as did low SES males (OR: 0.85, 95% CI: 0.78 to 0.93, *p* < 0.05) [65]. Marshal et al.’s [70] single-gender study on girls found a small positive association between low parent education and anxiety (standardised regression coefficient = 0.07, *p* < 0.01). Tao et al. [73] recruited young women of colour, assigned female at birth, and found evidence that self-reported social status was protective against anxiety (*p* < 0.001).

### Education

Three cross-sectional studies reported the association between education and anxiety [67, 73, 105]. Bauermeister et al. [67] recruited sexual minority cisgender males and found that anxiety was associated with having completed high school (compared to not completing) (*p* < 0.05) [67]. Tao et al.’s [73] female-only study found no evidence of an association between education level and anxiety. Shorey et al. [105] found no association between years of education and anxiety in males or females.

### Income and parental employment

One longitudinal study [129] and four cross-sectional studies explored associations between income and anxiety [68, 73, 89, 146]. Of the three studies reporting sex/gender-stratified results only, one identified a sex/gender difference. Ranta et al. [129] found evidence of an association between parental unemployment in the last 12 months and social phobia in males (OR = 2.2, 95% CI: 1.0 to 5.0, *p* < 0.05) but not females (OR = 0.5, 95% CI: 0.2 to 1.2, p-value not reported). Burke et al.’s [68] women-only study found no evidence of an association between household income and anxiety, however Tao et al.’s [73] female-only study found evidence that financial insecurity (*p* < 0.001) was a risk factor for anxiety.

### Family type and parental country of origin

One longitudinal study [120] found a positive association between anxiety and living with one parent in males (OR 1.98, 95% CI: 1.17 to 3.36, *p* < 0.05), but not in females (OR = 1.32, 95% CI: 0.94 to 1.87, p-value not reported). Living in an “other” family type (not with parents or in a shared residence) was associated with anxiety in females (OR 2.04, 95% CI: 1.02 to 4.08, *p* < 0.05) but not in males (OR = 0.67, 95% CI: 0.16 to 2.71, p-value not reported). Having two parents born outside the country of residence was associated with anxiety in males (OR 2.20, 95% CI: 1.37 to 3.53, *p* < 0.01) but not in females (OR = 1.18, 95% CI: 0.84 to 1.66, p-value not reported).

### Social media and related factors

This review identified nine cross-sectional studies [85, 98, 102, 106, 111, 126, 140, 143, 145].

that explored the associations between anxiety and social media and related factors. Vannucci et al. [143] found no evidence of sex/gender moderating the positive association between daily social media use and anxiety. Of the eight studies reporting sex/gender-stratified results only, four identified sex/gender differences. Hawes et al. [140] found evidence of a statistically significant sex/gender difference (sex/gender difference test: *p* = 0.04), such that time spent on social media was positively associated with social anxiety in females (*p* < 0.01) but not in males (p-value not reported). Kaltschik et al. [85] found that smartphone usage was a risk factor for anxiety in girls (*p* < 0.01) but not in boys (p-value not reported). Woodward et al. [102] found that TikTok use was a risk factor for anxiety in females (*p* = 0.001) but not in males (p-value not reported). Bilali et al. [98] found that the ‘conflict’ aspect of TikTok addiction (when TikTok interferes with daily activities) was a risk factor for anxiety in males (*p* < 0.006), but not females (*p* = 0.081).

### Religion

One cross-sectional study explored the association between religion and anxiety [95]. Of the five factors in the religious traits assessment, the Social Support factor (being active in faith or church) and conservatism (strictly following religious beliefs) were protective factors for anxiety in females (both *p* < 0.01), but not in males (p-value not reported). Stearns et al. [95] found a statistically significant sex/gender difference between males and females regarding the interaction between perceived maternal anxiety symptoms and religiosity in predicting anxiety (test for sex/gender difference: *p* < 0.001). This interaction was statistically significant in females (interaction *p* < 0.05), such that religiosity increased the positive association between maternal and daughters’ anxiety in females (*p* = 0.001).

## Interpersonal relationship level

Four groups of modifiable factors are captured in this level: general social support; bullying and victimisation; participation in romantic or sexual behaviours, and parental relationships. A simplified summary of the sex/gender-specific results is presented in Additional file 3, Appendix 6.

### General social support

One longitudinal [152] and two cross-sectional [64, 73] studies explored the association between social support and anxiety. No sex/gender differences were identified. Tao et al.’s [73] female-only study found no evidence of associations between anxiety and friendship intimacy or support (no p-values reported).

### Bullying and victimisation

Five longitudinal [93, 115, 120, 129, 141] and three cross-sectional studies [81, 99, 125] explored the association between anxiety and experiences of bulling and victimisation. Three studies [93, 99, 120] explored the moderating role of sex/gender, but found no evidence. Of the five studies reporting sex/gender-stratified results [81, 115, 125, 129, 141], three found evidence of sex/gender differences [125, 129, 141]. Leadbeater et al. [141] found that relational victimization (peer manipulation or peers damaging relationships or social status to cause harm) was a risk factor in females (*p* < 0.01), but not in males (p-value not reported); this sex/gender difference was statistically significant (test for sex/gender difference: interaction *p* < 0.001). Ranta et al. [129] also found that relational victimisation at age 15 was a risk factor for social phobia at age 17 in females (OR = 2.8, 95% CI: 1.0 to 7.7, *p* < 0.05), but not in males (OR = 0.3, 95% CI: 0.1 to 2.3), whereas direct victimisation (peers’ direct acts of verbal or physical aggression e.g., hitting, pushing, name-calling) at age 15 was a risk factor for social phobia at age 17 in males (OR = 2.6, 95% CI: 1.1 to 6.3, *p* < 0.05), but not in females (OR = 1.2, 95% CI: 0.4 to 4.0). Sares-Jaske et al. [125] indicated that, out of all gender groups, previous experience of being bullied had a greater positive association with anxiety (OR = 32.7, 95% CI: 29.0 to 37.0) and social anxiety (OR = 13.0, 95% CI: 11.5 to 14.8) in transmasculine young people, when compared to the reference group (cisgender boys with no previous experience of being bullied); the 95% confidence intervals for the transmasculine young people did not overlap with those for other gender groups, indicating evidence at the 5% level of a gender difference. However, when compared to participants of the same gender group with no previous experience of being bullied, the experience of being bullied had a greater positive association with anxiety (OR = 6.47, 95% CI: 5.80 to 7.22) and social anxiety (OR = 2.24, 95% CI: 2.13 to 2.36) in cisgender boys. The 95% confidence interval for the association for cisgender boys did not overlap with those for other gender groups, indicating a statistically significant gender difference at the 5% level.

### Romantic relationships and sexual behaviours

One longitudinal study [152] and two cross-sectional studies [72, 116] explored the associations between romantic relationships and sexual behaviours and anxiety. One study reported sex/gender-stratified results [152] and another explored the moderating role of gender [116].

Whitton et al.’s [72] study on emerging adults (aged 18 to 20 years) assigned female at birth (cisgender female, transgender male, non-binary assigned female at birth) found evidence of an association between relationship involvement and anxiety being moderated by gender identity (*p* = 0.01), sexual identity (*p* = 0.03), and partner gender (*p* < 0.01). Cisgender and lesbian participants who were in a relationship reported lower levels of anxiety than single cisgender and lesbian females respectively (both *p* < 0.01). However, this was not true for gender minority participants, bisexual/pansexual participants or participants with other sexual identities.

### Relationships with parents

Two longitudinal [134, 144] and ten cross-sectional studies [71, 86, 90, 94, 96, 100, 114, 132, 133, 142] explored the associations between parental relationships and anxiety.

#### Positive parental relationships

One longitudinal [134] and five cross-sectional studies [90, 96, 100, 114, 142] reported the sex/gender stratified associations between positive parental relationships and anxiety, and all identified sex/gender-specific factors [90, 96, 100, 114, 134, 142]. Mckinney et al. [90] found that, for females, both paternal and maternal caring were protective factors for anxiety (*p* < 0.01), whereas for males, only paternal caring was a protective factor (*p* < 0.01). Benedetto et al. [114] found that, while paternal and maternal emotional availability were protective factors for anxiety in males (*p* < 0.05, *p* < 0.01, respectively), only maternal emotional availability was a protective factor in females (*p* < 0.01). Apsley et al. [134] found that maternal connection was a protective factor for males (*p* = 0.001) but not females. Carollo et al. [100] found that parental care was a protective factor against anxiety and worry in males (*p* = 0.002, *p* < 0.001, respectively ), but not females. Van Beusekom et al. [142] found that mother and father acceptance (warmth, affection, and care that parents express toward their children) were protective factors for anxiety in both males and females (all *p* < 0.01). In the ‘boys’ subgroup, father acceptance moderated the association between gender nonconformity and anxiety (interaction *p* < 0.001); father acceptance reduced the association between gender nonconformity and anxiety (*p* < 0.001). In the ‘girls’ subgroup, mother acceptance moderated the association between same-sex attraction with anxiety (interaction p = 0.003); mother acceptance reduced the association between same-sex attraction and anxiety (*p* < 0.003). Sebokova et al. [96] found evidence that in boys, family cohesion (emotional bonding between family members) moderated the association between self-consciousness and anxiety (interaction *p* < 0.05), such that high levels of family cohesion increased the association between self-consciousness and anxiety (*p* < 0.05). In girls, family adaptability (ability to respond to stressful situations) moderated the association between self-consciousness and anxiety (interaction *p* < 0.05), such that family adaptability reduces the association between self-consciousness and anxiety (*p* < 0.05). Additionally, in girls, family communication moderated the association between self-consciousness and anxiety (interaction *p* < 0.05), such that family communication reduces the association between self-consciousness and anxiety (*p* < 0.05).

#### Parenting styles

Four cross-sectional studies explored the association between various parenting styles and anxiety [86, 90, 94, 100]. One study explored and found no evidence of sex/gender moderation [86] and three studies reported sex/gender stratified results [90, 94, 100]. Two studies [90, 100] identified sex/gender differences. Mckinney et al. [90] found that maternal and paternal authoritative parenting styles (parents are nurturing, responsive, and supportive, yet set firm limits for their children) were protective factors for anxiety in females (*p* < 0.01), but not in males. Both paternal and maternal overprotection was a risk factor for anxiety in females (*p* < 0.01), whereas for males, only paternal overprotection was a risk factor (*p* < 0.01). Carollo et al. [100] found that parental overprotection was a risk factor for anxiety in females (*p* = 0.008), but not in males, and a protective factor against worry in females (*p* < 0.001), but not males.

#### Autonomy support

Two cross-sectional studies explored the relationship between autonomy support (children feel in control of their actions and decisions) and anxiety [86, 114]. Kouros et al. [86] found evidence of sex/gender moderating the negative association between autonomy support and anxiety (interaction *p* = 0.019), such that autonomy was a protective factor against anxiety in males (*p* = 0.002) but not in females (*p* = 0.1). Benedetto et al. [114] found that paternal autonomy support was a protective factor against anxiety in males (*p* < 0.01), whereas maternal autonomy support was a protective factor in females (*p* < 0.01).

#### Negative parental behaviours

Two longitudinal [134, 144] and three cross-sectional studies [71, 132, 133] examined the relationship between negative parental behaviours and anxiety. Two studies tested for and found evidence of sex/gender moderation [132, 133], two reported sex/gender stratified results and identified sex/gender differences [134, 144], and one was a single-sex study on young gay men [71] which found evidence of a positive association between parental disapproval and anxiety (*p* < 0.05).

Thornhill et al. [133] found evidence that sex/gender moderated the positive association between intracultural family accusations of assimilation and anxiety (interaction *p* < 0.01), such that intracultural accusations of assimilation were associated with anxiety in males (*p* < 0.001), but not in females (*p* > 0.05). Kim et al. [132] found that gender moderated the interaction between discrimination and family hostility in predicting anxiety (interaction *p* < 0.01) (See section ‘Experience of discrimination, stigma and prejudice’).

Apsley et al. [134], found evidence that maternal psychological control (manipulative and intrusive behaviours to control a child’s thoughts and feelings) was a risk factor for anxiety in females (*p* = 0.005) but not in males (*p* ≥ 0.05). Wijsbroek et al. [144] found that for males, parental psychological and behavioural control were risk factors for anxiety at baseline (*p* < 0.001, *p* < 0.05, respectively) and two years after baseline (*p* < 0.05, *p* < 0.001) in males. However, an association between psychological control at two years after baseline and anxiety four years after baseline was found in females (*p* < 0.001), but not in males (*p* ≥ 0.05).

## Local community level

Three groups of modifiable factors are captured in this level: social isolation; community networks; residential greenness; school-related factors and safety, crime and violence. A simplified summary of the sex/gender-specific results is presented in Additional file 3, Appendix 7. Three cross-sectional studies explored the association between social isolation and anxiety [96, 106, 112]; two studies reported sex/gender-stratified results and found no evidence of sex/gender differences [96, 106], and the other found no evidence of sex/gender moderation [112]. Gillespie et al.’s [131] longitudinal study on resettled Somalian young people found a positive association between assimilation (orientation towards new culture of residence and away from the culture of origin) and anxiety in males (*p* < 0.01) but not in females [131]. Larsen et al.’s [109] longitudinal study found no evidence of an interaction between sex, age and residential greenness (access to green spaces) in predicting anxiety. Surprenant et al.’s [146] cross-sectional study found evidence of a positive association between time doing homework and anxiety in females (*p* < 0.001), but not males (p-value not reported). Fontaine et al.’s [139] longitudinal study found no evidence of sex differences; non-violent delinquency and violent delinquency at age 15 years were positively associated with anxiety at age 17 years in males (both *p* ≤ 0.001) and females (*p* ≤ 0.05 and *p* ≤ 0.01, respectively). Non-violent delinquency and violent delinquency at age 17 years were also positively associated with anxiety at age 17 years in males and females (both *p* ≤ 0.001).

## Wider environment and society level

Two modifiable factor groups fell under this category: experience of discrimination, stigma and prejudice; plus the Covid-19 pandemic. A simplified summary of the sex/gender-specific results is presented in Additional file 3, Appendix 8.

### Experience of discrimination, stigma and prejudice

Fourteen cross-sectional studies [67, 68, 71, 73, 74, 91, 92, 103, 110, 121, 122, 130, 132, 138] and three longitudinal studies [79, 93, 131] explored the association between anxiety and risk factors related to discrimination, stigma and prejudice. Nine studies reported the moderating role of sex/gender [79, 91–93, 103, 122, 130, 132, 138], three studies reported gender-stratified results [110, 121, 131] and three had single-sex samples [67, 68, 71].

Of the nine studies investigating the moderating role of sex/gender, four found evidence of 2-way interactions between sex/gender and discrimination (based on ethnicity, race and sexuality, respectively) in predicting anxiety [93, 103, 122, 130]. Poteat et al. [93] found evidence of sex/gender moderation (interaction *p* < 0.05), such that homophobic victimisation was a risk factor for males (*p* < 0.001) but not females (*p* = 0.28). Cano et al. [130] found evidence of sex/gender moderating the relationship between anxiety and ethnicity-based discrimination via social media (interaction *p* = 0.01), such that social media discrimination was a risk factor for anxiety in males (*p* ≤ 0.001), but not in females (*p* = 0.58). Grapin et al. [103] found evidence of an interaction between gender and online racial discrimination in predicting social anxiety (interaction: *p* = 0.019), such that online racial discrimination was a risk factor in women (*p* = 0.014) but not in men (*p* = 0.177). Lee et al. [122] found evidence of sex/gender moderating the positive association between anxiety and internalised sexual stigma in gay and bisexual young people (interaction *p* < 0.05), such that internalised sexual stigma was a risk factor in females (*p* < 0.001), but not in males (*p* = 0.252).

Of the eight studies investigating sex/gender moderation, four found evidence of 3-way interactions between sex/gender, discrimination and an additional variable in predicting anxiety [91, 92, 132, 138]. Kim et al. [132] found that gender moderated the interaction between discrimination and family hostility in predicting anxiety (interaction *p* < 0.01), such that family hostility reduced the association between discrimination and anxiety in women (*p* < 0.01) but not in men (*p* > 0.1). El-Sheikh et al. [138] found evidence of sex/gender moderation, such that poor sleeping habits exacerbated the relationship between discrimination and anxiety in females only. Sex/gender moderated the interaction between general discrimination and minutes asleep in predicting anxiety (interaction *p* < 0.01), such that duration of sleep was protective against the association between general discrimination and anxiety in females (*p* < 0.001). Sex/gender moderated the interaction between racial discrimination and minutes slept in predicting anxiety (interaction *p* < 0.05), such that sleep in minutes was protective in the association between racial discrimination and anxiety in females (*p* < 0.001). Sex/gender moderated the interaction between racial discrimination and sleep efficiency in predicting anxiety (interaction *p* < 0.01), such that sleep efficiency was protective against the association between racial discrimination and anxiety in females (*p* < 0.001) but not males. Sex/gender moderated the interaction between racial discrimination and sleep variability in predicting anxiety (interaction *p* < 0.05), such that sleep variability was protective against the association between racial discrimination and anxiety in females (*p* < 0.001) but not males. Neblett et al. [91] found evidence of sex/gender moderation (interaction *p* = 0.028) such that males from lower SES backgrounds (*p* < 0.001) and females from higher SES backgrounds (*p* = 0.002) were at greater risk of the anxiety associated with racial discrimination. Perkins et al. [92] found evidence of sex/gender moderation (interaction *p* < 0.01) such that a higher public regard (perceived societal sentiment toward one’s racial group) exacerbated the association between offline discrimination and anxiety in Black males (*p* < 0.001) and reduced the association in Black females (*p* < 0.001). Sex/gender also moderated the interaction between online discrimination and public regard in predicting anxiety (interaction *p* < 0.05), such that public regard was a protective factor against anxiety associated with the online discrimination in Black females only (*p* = 0.001).

None of the three studies reporting only sex/gender-stratified results found statistically significant sex/gender differences [110, 121, 131].

Five studies had single sex/gender samples. Burke et al. [68] found that in Black women, gendered racial microaggression stress was a risk factor of social anxiety (*p* < 0.001) and generalised anxiety (*p* < 0.01). Buckner et al.‘s [74] female-only study found that past-year experiences of sexism was a risk factor for anxiety (*p* < 0.01). Tao et al. [73] female-only study found that, in young women of colour, exposure to gendered racial discrimination (*p* < 0.001) and oppression awareness socialization (*p* < 0.05) were risk factors for anxiety. Tao et al. [73] also found evidence of the indirect effect of discrimination on anxiety via co-rumination with friends (standardised regression coefficient = 0.043, 95% CI: 0.001 to 0.091, no p-values reported) and the direct effect exposure to discrimination to anxiety symptoms (standardised regression coefficient = 0.16, 95% CI: 0.036 to 0.25, no p-values reported), indicating that gendered racism co-rumination with friends partially mediated the association between exposure to discrimination and anxiety. Bauermeister et al.’s [67] male-only study found that being told to stop acting feminine (*p* < 0.001) and disciplinary actions related to gender policing (*p* < 0.005) were risk factors for anxiety. Pachankis et al.’s [71] male-only study found that sexual orientation concealment (*p* < 0.01), public self-consciousness (*p* < 0.01) and parent disapproval (*p* < 0.05) were risk factors for anxiety.

### Covid-19 pandemic

One cross-sectional [87] and two longitudinal studies [117, 123] explored the impact of Covid-19 on anxiety. Two studies found evidence of sex/gender moderation [87, 123]. Minhas et al. [123] collected data before and during Covid-19 and found evidence of an interaction between sex/gender and time (interaction *p* = 0.00988), such that there was an increase in anxiety in females (*p* < 0.001), but not in males (p-value not reported) [123]. Kornilaki et al. [87] found evidence of an interaction between sex/gender and life disruption due to Covid-19 in predicting anxiety (interaction *p* = 0.010), such that the association was stronger in females compared to males (p-values not reported). De France et al. [117] found statistically significant evidence of sex/gender differences in the association between fear of Covid-19 and anxiety (*p* = 0.001), such that the association was higher for males than females (p-values not reported).

## Discussion

This systematic review summarises the evidence base of studies identifying modifiable sex/gender-specific risk and protective factors for anxiety in young people aged 16–24 years. We grouped the risk and protective factors into the following levels: individual; interpersonal relationships; local community; and wider environment and society. There was evidence indicating that the following risk factors may be more anxiety-inducing in females than in males: early alcohol use initiation and social media use, from the individual level; parental overprotection, from the interpersonal level, and gender-based harassment / violence from the wider environment and society level. A large amount of heterogeneity and methodological limitations in the included studies have resulted in conflicting evidence regarding many of the factors explored. This makes it difficult to form a theoretical contribution as to why some factors are sex/gender-specific and why girls and young women experience such high levels of anxiety, restricting any insights on intervention development. What this review does provide is a critique of the evidence base and a signpost for future research.

For physical health and health behaviours, potential sex/gender-specific risk factors included early onset al.cohol consumption in females [78, 119, 154]. Berenz et al. [78] attributed this to an increased risk of sexual or physical assault [78, 155]. However, as cross-sectional studies cannot demonstrate causality, drinking could be a coping mechanism for pre-existing anxiety. No clear sex/gender differences emerged in the associations between anxiety and physical activity, although studies have found that in girls, the social aspects of physical activity have more influence on mental health than physical activity itself [156–161].

This review has revealed conflicting findings regarding sex/gender differences in the association between anxiety and social media. Of the nine studies exploring these relationships, three found evidence that social media use was a risk factor for anxiety in girls but not in boys [85, 102, 140], one found the reverse pattern [98], and five found no difference [106, 111, 126, 143, 145]. Keles and Platt [162] propose that aspects of social media, such as online harassment and social comparison through highly idealised representation of peers, influencers and celebrities [11, 163], underlie internalizing symptoms in girls [140, 164, 165]. Preoccupation with, and failure to meet, the unrealistic standards of beauty promoted on social media results in body dissatisfaction and body image concerns [11, 163, 166–168] and have been identified as risk factors for internalizing problems for girls [169–172]. Girls are more likely to be ‘High Communicators’ and ‘Broadcaster’ user types [173] and these different social media experiences may contribute to the gender gap in anxiety [11, 23, 25, 163, 174]. Exposure to online misogyny could be another contributor to social media having a stronger impact on girls’ anxiety [175–179]. These conflicting findings could be a result of heterogeneity in measures of social media use. Indeed, the three studies that identified sex/gender-specific risk factors [85, 102, 140] used similar measures: ‘time on social media’ [102, 140] and ‘smart phone use’ [85], while other studies’ varied, for example ‘TikTok Addiction scale’ [98].

Of the six studies exploring sexual harassment or abuse, three studies found evidence of sex/gender specific risk factors [75, 82, 99]. These conflicting findings could be a result of a heterogeneity in exposure types. Indeed, the three studies that found evidence of a sex/gender-specific risk factor all explored online sexual harassment [75, 82, 99]. The remaining studies investigated broader experiences of sexual abuse or harassment with different measures [81, 88, 104]. Gasso et al. found that receiving a sext, being a victim of nonconsensual dissemination, and being pressured or threatened to sext were risk factors for anxiety in females but not in males [82]. However, Carlberg et al. found that unwanted online sexual solicitation was a greater risk factor for anxiety in males [99]. Gasso et al. [82] and Carlberg et al. [99] use different measures of anxiety (Brief Symptom Checklist and The Revised Child Anxiety and Depression Scale, respectively) and age groups (21.4 and 16.70 years, respectively). This heterogeneity could account for the conflicting results. This matches the wider evidence base of the mental health implications of online sexual harassment, where inconsistent terminology, measures and analysis result in conflicting findings [180, 181]. Nevertheless, studies have reported girls and women experiencing more distress following online sexual harassment [175–179]. This has been attributed to online sexual harassment being perceived as ‘an expression of gender hierarchies and patriarchal ideology’ [99, 180, 182, 183], and a reinforcement of the threat of sexual violence, producing feelings of fear, helplessness and disempowerment, which has negative implications for anxiety [99, 184, 185].

The conflicting findings for the gendered negative impact of bullying and victimisation could also be attributed to the heterogeneous evidence base with diverse measures of anxiety and types of bullying and victimisation; of the three studies that found evidence of sex/gender-specific risk factor [125, 129, 141], two found that relational victimization was a risk factor for anxiety in females only [129, 141]. Keyes and Platt [162] suggest that girls are more vulnerable to interpersonal stressors and resultant anxiety because they are more likely to experience sexual harassment, and intimate partner violence [186], whereas boys are more likely to experience physical violence. One female-only study explored modifiable factors related to peer relationships in this review; friendship intimacy or friendship support were not associated with anxiety [73]. However, an association between peer relationships and anxiety has been found in other age groups [61, 187–189], and Chui et al.’s [187] meta-analysis of cross-sectional studies with age-ranges outside of this review’s, found no evidence of gender moderating the relationship between friendship quality and social anxiety. Nevertheless, girls are often regarded as more sensitive to distress in peers [32, 190, 191], and more likely to rely on peers for emotional support. Thus, interpersonal stressors may have a greater impact on their mental health compared to boys [156, 192].

Parental overprotection appeared to have a stronger association with anxiety in females: the socialization of boys to be more independent may shield them from anxiety associated with overprotection [90, 100]. Girls are reportedly more affected by negative family interactions [193–195], but Telzor et al. [196], investigating internalizing symptoms rather than anxiety, found similar associations in boys and girls. Telzor et al. [196] suggested that their finding that girls experience more frequent negative daily family interactions, may explain higher levels of internalizing symptoms, perhaps because they are more likely than boys to rely on family for emotional support and to strive for harmonious relationships [196]. Telzer et al. [196] also found that positive family relationships accounted for gender differences in internalizing symptoms, and with Klasen et al. [197], suggested that interventions focus on improving communication and the relational context of families. This review identified a potential sex/gender difference where the association between autonomy-restricting parenting styles and anxiety was stronger in males [86, 114]. Kouros et al. [86] attributed this to the gender intensification hypothesis [198], where socialization encourages traditional gender roles [199, 200], with boys expecting more independence and experiencing anxiety when their autonomy is restricted [86, 94].

In terms of community-level modifiable factors, no clear sex/gender-specific risk factors emerged, likely due to the diverse exposure measures, for example social isolation, assimilation and residential greenness [96, 106, 109, 131]. Only one study explored sex/gender differences in the relationship between anxiety and school-related factors, and found evidence that time spent doing homework was associated with anxiety in females, not males [146]. This aligns with prior findings that girls are more likely to internalize academic pressure, school stress and other school-related factors and have heightened emotional responses to academic stressors [201], while boys externalise stress, and become cynical towards school [202].

No included studies explored the role of employment, the workplace, the school-to-work transition or educational settings beyond schoolwork. Employment is an important contributing factor to young people’s wellbeing and identity [203], and the school-to-work transition can exacerbate or trigger mental health problems [47]. Adjusting to adult roles, completing secondary education, obtaining employment, enrolling in post-secondary education or vocational training, financial concerns and living independently can also contribute to a mental health burden [44–46]. Having to maintain sleep, diet, work schedules and peer relationships during this time of transition results in an increased risk for onset of mental health problems [204]. Uncertainty around decisions about the future, for example deciding on school subjects and career paths, have been cited as a contributor to the increase in mental health problems among young people [43]. There is a lack of research exploring this transitional period of young adulthood [48] and whether there are any sex/gender differences in how it is experienced or impacts on anxiety. There is also a lack of evidence demonstrating if or how sex/gender influences exposure to workplace stressors and the role of the workplace in sex/gender differences in anxiety levels in young adulthood.

Turning to wider societal factors, there is little research exploring the impact of sexism on young women’s anxiety. One female-only study in this review explored this: Buckner et al. [74] found that past-year experience of sexism was a risk factor for anxiety. Everyday sexism can include comments that promote gender stereotypes and question women’s competence and experiences of sexual objectification. These experiences may contribute to anxiety [205] through their negative impact on women and girls’ self-esteem and self-efficacy [206–208]. Shute et al. [209] and Hartas et al. [11] propose that claims that we live in a post-feminism society, despite patriarchal social systems still being present could be a contributing factor to girls’ and young women’s anxiety.

Two studies in this review considered the intersection of race and sex/gender when exploring the association between gendered racial discrimination and anxiety. Burke et al. [68] and Tao et al. [73] found that in young women of colour, gendered racial microaggressions and discrimination were risk factors for anxiety. Intersectionality underscores the idea that an individual’s identity is comprised of many different characteristics and was initially constructed to encapsulate Black women’s experiences of racism and sexism [210, 211]. Although not investigating anxiety in young people 16–24 years, Mossakowski et al. [212] found that discrimination was more psychologically distressing among women in Hawaii compared to men, and attributed this to the ‘double jeopardy of sexism and racism’ [213] exacerbating the relationship between discrimination and mental health due to women ruminating about whether the discrimination was gender- or ethnicity-based [214]. This demonstrates the importance of examining the intersection of ethnicity and gender, and other identities such as social class, disability and sexuality, when exploring gender differences in anxiety.

Three studies found evidence of factors that protected against the association between discrimination and anxiety in females but not males [92, 132, 138]. This potentially supports the stress vulnerability hypothesis, which posits that the effect of discrimination on mental health is determined by an individual’s access to resources to cope with the negative consequences of stress [215–217]. Alternatively, the stress exposure hypothesis proposes that the effect discrimination has on mental health is determined by the amount of exposure; Individuals of more disadvantaged social status may have greater exposure and therefore more negative mental health effects [218, 219].

### Methodological limitations of the evidence base

Several limitations emerged which are important to address in future research. Firstly, 62 studies were cross-sectional and unable to demonstrate the direction of influence between proposed modifiable factors and outcomes. Of the studies reporting ethnicity and socioeconomic status, participants were predominantly Caucasian and from middle-class socio-economic status backgrounds, limiting the generalizability of findings. There was also a sex/gender imbalance with 32 studies having more than 60% of one sex/gender [64, 68, 76–105]. The overrepresentation of females in the sample introduces a risk of detecting an association between anxiety and the modifiable factor in the female subgroup, but not in the male subgroup. This is an issue for small sample sizes, for example Kouros et al.’s [86] sample size of 116 and its 83.1% female limits its precision.

The studies included in this review lacked clarity around how they differentiated between sex and gender [149]. The interchangeability of these terms introduces a risk of misgendering participants. Where questionnaires do not distinguish between sex and gender participants will interpret the question themselves using their understanding of terminology, increasing the risk of mismeasurement for participants where sex and gender differs [151]. Using a single measure of gender, for example a questionnaire which asks if participants’ gender is: ‘boy or girl’ [125] or ‘man or woman’ [116], fails to capture the complexity and fluidity of gender, potentially limiting the quality of findings [151, 220]. As gender encapsulates identity, expression, social status and norms, when studies do not specify the aspect of gender being captured, participants’ responses may differ [151]. Future research must avoid using sex and gender interchangeably and be explicit about how to answer questions related to sex or gender [153]. These questions could be improved by assessing gender-specific psychosocial factors, such as identification with gender roles, gender stereotypes, or measurements of femininity and masculinity [20, 151].

The approach to sex/gender analysis varied from exploring the moderating role of sex/gender to reporting sex/gender stratified associations between anxiety and the modifiable factors. Of the studies reporting sex/gender stratified results, 41 studies had no statistical tests for sex/gender differences to formally identify sex/gender specific risk factors. In some studies an association was statistically significant in one sex/gender group but not the other, but as the confidence intervals overlap there may not be a statistically significant sex/gender difference [78, 82, 84, 98, 129, 146]. Few studies report confidence intervals; of the 41 studies with no formal tests for sex/gender differences, only eight report confidence intervals [71, 82, 89, 94, 98, 129, 137, 146], resulting in potential over-estimation of sex/gender differences.

### Strengths and limitations of the systematic review process

One limitation of this review was the exclusions made after full-text screening to focus the review, introducing the risk of selection bias. All exclusions were deliberated on with the supervisory team and YPAG members. While excluding studies from LMICs was appropriate for the review due to its aim to inform potential UK-based gender-specific mental health interventions, it does not guarantee that the included studies have comparable social, economic and cultural contexts. Excluding LMICs prevents any attempt at addressing global disparities [221]. Further research is required to identify modifiable sex/gender-specific risk and protective factors for anxiety among young people in LMICs and non-Western countries. Studies that were not peer reviewed academic journals and non-English studies were excluded, introducing a risk of publication bias and reducing the generalisability of findings to non-English speaking populations.

The strengths of this systematic literature review include a detailed search strategy. Rather than including gender difference* the search strategy included gender*, which was more appropriate given the inclusion of single-sex/gender studies. This increased the amount of screening but reduced the risk of overlooking relevant studies. Another strength of this review is the PPIE work undertaken with the YPAG to focus the review and, in subsequent sessions, interpret findings. The systematic review protocol was registered, and amendments (the additional exclusions to focus the systematic review) were submitted. This is the first systematic review synthesising studies identifying modifiable sex/gender-specific risk and protective factors for anxiety among 16–24-year-olds in HICs. This is a key age group to consider given the sex/gender differences in anxiety levels that emerge here.

### Future research and implications for practice

This review identifies numerous opportunities for further research, that might help inform future interventions tailored to the needs of each gender. There is a need for longitudinal research that (i) cover a longer period of time with more frequent follow-ups than the studies in this review, (ii) include more ethnically and socially diverse populations, and (iii) provide more clarity around how information on sex and gender is obtained.

The conflicting results in this review warrant further research of modifiable risk and protective factors to confirm how sex/gender moderates their relationships with anxiety. Further longitudinal research is required that explores sex/gender differences in the association between discrimination and anxiety in 16-24-year-olds, the intersection of ethnicity, gender and anxiety, and the role of online misogyny. Girls and young women are being exposed to online misogyny at increasingly high levels [222]; 73% of girls aged 11–16 and 84% of young women aged 17–21 reported facing sexism online [223]. Therefore, it is imperative that further longitudinal research is conducted to explore these relationships. Such work may have the potential to inform gender-sensitive mental health interventions.

This review identified risk factors that have not been examined as a sex/gender-specific risk factor for anxiety in 16–24 year olds, despite being known risk factors for mental health in general. These factors include gender discrimination, peer relationships, the school/college context, the workplace and the school-to-work transition [203]. Future research should synthesise qualitative studies regarding gender specific risk factors for anxiety, to complement and add nuance to the quantitative findings summarized here by providing insights into the reasons behind associations. Although conflicting findings and methodological limitations limit the ability of this review to directly inform future sex/gender-specific interventions, this review has the potential to do so indirectly by signposting future research that will improve the evidence base. Herrmann et al. [19] argue that gender-specific interventions can: respond to gender-specific needs and experiences; provide a safe space and a supportive, empowering environment; be more effective than gender-non-specific interventions and can address gender disparities and contribute to the achievement of social justice. Hermann et al. [19] identified a lack of interventions for anxiety tailored for girls; interventions that do not address girls’ unique developmental, social, and psychological needs limit their effectiveness [224]. Future research is required to explore what girls and young women want and need in gender-specific mental health interventions for anxiety, building an evidence base that supports effective, high impact mental health promotion and improves outcomes for this high-risk group [224].

## Conclusions

This systematic review indicates that sex/gender differences may exist in the modifiable risk and protective factors for anxiety among 16–24 year olds. The least contradictory evidence indicates that early alcohol use initiation, parental overprotection and social media experiences, in particular online sexual harassment, may be more anxiety-inducing in females than in males, however methodological limitations of the evidence base limit the certainty of this review and these conclusions should be considered cautiously. Future longitudinal studies are required that explore a wider range of risk and protective factors.

## Supplementary Information

Additional file 1.

Additional file 2.

Additional file 3. Appendix 1. Included studies characteristics. Appendix 2. National Institutes of Health Quality Assessment Tool. Appendix 3. Quality assessment of included studies. Appendix 4. Certainty of evidence GRADE assessment. Appendix 5. Individual-level sex/gender results. Appendix 6. Interpersonal-level sex/gender results. Appendix 7. Local community-level sex/gender results. Appendix 8. Wider society-level sex/gender results.

## Data Availability

All data analysed during this study are included in this published article and its supplementary information files.

## Abbreviations

DSM-5: Diagnostic and Statistical Manual of Mental Disorders
PRISMA: Preferred Reporting Items for Systematic Reviews and Meta-Analysis
PPIE: Patient public involvement and engagement
YPAG: Young People’s Advisory Group
NIHR ARC West: National Institute for Health and Care Research Applied Research Collaboration
CT: Ciara Thomas
LA: Leah Attwell
LB: Lucy Biddle
MJL: Myles-Jay Linton
UO: Obioha Ukoumunne
TF: Tamsin Ford
JK: Judi Kidger
NIH: National Institutes of Health
LMICs: Lower-middle income countries
HICs: High income counties
GRADE: Grading of Recommendation, Assessment, Development and Evaluation
OR: Odds ratio
CI: Confidence interval
SES: Socioeconomic status
ACEs: Adverse childhood experiences
