# Supplementary material for: Exploring the role of modifiable sex/gender-specific risk and protective factors for anxiety among young people in high income countries: a systematic narrative review
Source: BMC Public Health. 2026 Feb 18;26:1292. doi: 10.1186/s12889-026-26447-9 (PMC13097721; doi:10.1186/s12889-026-26447-9)
Supplement: Supplementary file 1 — Additional file 1. [file 12889_2026_26447_MOESM1_ESM.docx]

**Additional file 1: Database searches**

Table of Contents

[Initial search: 29^th^ February 2024 1](#_Toc203126454)

[Ovid Medline 1](#_Toc203126455)

[APA PsychInfo 4](#_Toc203126456)

[Embase 7](#_Toc203126457)

[Web Of Science 10](#_Toc203126458)

[Sociological abstracts: ERIC, International Bibliography of the Social Sciences (IBSS)‎ 12](#_Toc203126459)

[Scopus 12](#_Toc203126460)

[Update search: 4^th^ July 2025 13](#_Toc203126461)

[Ovid Medline 13](#_Toc203126462)

[APA PsychInfo 13](#_Toc203126463)

[Embase 13](#_Toc203126464)

[Web Of Science 14](#_Toc203126465)

[Sociological abstracts: ERIC, International Bibliography of the Social Sciences (IBSS)‎ 14](#_Toc203126466)

[Scopus 14](#_Toc203126467)

# Initial search: 29^th^ February 2024

## Ovid Medline

Database(s): **Ovid MEDLINE(R)**1946 to present (29th February 2024)
Search Strategy:

| **#** | **Searches** | **Results** |
| --- | --- | --- |
| 1 | Adolescent/px [Psychology] | 298 |
| 2 | Adolescen*.ab,ti. | 352140 |
| 3 | Young Adult/px [Psychology] | 58 |
| 4 | young adult*.ab,ti. | 121691 |
| 5 | youth.ab,ti. | 87682 |
| 6 | emerging adult*.ab,ti. | 3954 |
| 7 | teen*.ab,ti. | 35387 |
| 8 | young people*.ti,ab. | 38023 |
| 9 | high school student*.ti,ab. | 12502 |
| 10 | secondary school student*.ti,ab. | 3230 |
| 11 | 1 or 2 or 3 or 4 or 5 or 6 or 7 or 8 or 9 or 10 | 546706 |
| 12 | sex difference*.ti,ab. | 43539 |
| 13 | gender difference*.ti,ab. | 37864 |
| 14 | Sex Factors/ | 279751 |
| 15 | gender-sensitiv*.ti,ab. | 1168 |
| 16 | gender-specific*.ti,ab. | 14557 |
| 17 | sex-specific*.ti,ab. | 29520 |
| 18 | gender-related.ti,ab. | 6074 |
| 19 | gender sensitiv*.ti,ab. | 1168 |
| 20 | gender specific*.ti,ab. | 14557 |
| 21 | sex specific*.ti,ab. | 29520 |
| 22 | gender related.ti,ab. | 6074 |
| 23 | genderrelated.ti,ab. | 5 |
| 24 | gender gap*.ti,ab. | 2185 |
| 25 | gender-gap*.ti,ab. | 2185 |
| 26 | gender*.ti,ab. | 426662 |
| 27 | 12 or 13 or 14 or 15 or 16 or 17 or 18 or 19 or 20 or 21 or 22 or 23 or 24 or 25 or 26 | 688427 |
| 28 | exp epidemiologic studies/ | 3247572 |
| 29 | Longitudinal study.ti,ab. | 74877 |
| 30 | Observational study.ti,ab. | 123942 |
| 31 | Cohort study.ti,ab. | 296569 |
| 32 | Case control study.ti,ab. | 111138 |
| 33 | Time series study.ti,ab. | 1816 |
| 34 | Prospective study.ti,ab. | 162941 |
| 35 | Retrospective study.ti,ab. | 215980 |
| 36 | Follow-up.ti,ab. | 1221826 |
| 37 | Cross-sectional study.ti,ab. | 255884 |
| 38 | risk factors/ | 974269 |
| 39 | risk factor*.ti,ab. | 771012 |
| 40 | protective factors/ | 6432 |
| 41 | correlat$.ab,ti. | 2294054 |
| 42 | causal factor*.ti,ab. | 6499 |
| 43 | associat$.ab,ti. | 5682272 |
| 44 | high risk.ti,ab. | 370985 |
| 45 | at risk.ti,ab. | 219924 |
| 46 | "resource factor*".ti,ab. | 200 |
| 47 | predictor*.ti,ab. | 506073 |
| 48 | determinant*.ti,ab. | 287231 |
| 49 | (protective adj (factor* or characteristic*)).ti,ab. | 29521 |
| 50 | (predictive adj (factor* or characteristic*)).ti,ab. | 36019 |
| 51 | "correlation of data"/ | 2774 |
| 52 | correlat$.ab,ti. | 2294054 |
| 53 | exp Regression Analysis/ | 462749 |
| 54 | regression$.ab,ti. | 1091041 |
| 55 | multivariate analysis/ | 132212 |
| 56 | multivariate analysis.ti,ab. | 189006 |
| 57 | etiolog*.ti,ab. | 318378 |
| 58 | 28 or 29 or 30 or 31 or 32 or 33 or 34 or 35 or 36 or 37 or 38 or 39 or 40 or 41 or 42 or 43 or 44 or 45 or 46 or 47 or 48 or 49 or 50 or 51 or 52 or 53 or 54 or 55 or 56 or 57 | 10798995 |
| 59 | anxiet$.ab,ti. | 265508 |
| 60 | panic*.ti,ab. | 23774 |
| 61 | worry*.ti,ab. | 18362 |
| 62 | worri*.ti,ab. | 15450 |
| 63 | fear*.ti,ab. | 108517 |
| 64 | exp Fear/et, px [Etiology, Psychology] | 7322 |
| 65 | Stress, Psychological/ep, et, px [Epidemiology, Etiology, Psychology] | 51716 |
| 66 | Psychological Distress/ | 4457 |
| 67 | Psychological distress.ti,ab. | 28015 |
| 68 | psychological stress*.ti,ab. | 11072 |
| 69 | Anxiety Disorders/ep, et, px [Epidemiology, Etiology, Psychology] | 22707 |
| 70 | Anxiety, Separation/ep, et, px [Epidemiology, Etiology, Psychology] | 1172 |
| 71 | Panic Disorder/ep, et, px [Epidemiology, Etiology, Psychology] | 4273 |
| 72 | Phobic Disorders/ep, et, px [Epidemiology, Etiology, Psychology] | 6213 |
| 73 | Agoraphobia/ep, et, px [Epidemiology, Etiology, Psychology] | 1700 |
| 74 | Phobia, Social/ep, et, px [Epidemiology, Etiology, Psychology] | 645 |
| 75 | separation anxiet*.ti,ab. | 1778 |
| 76 | panic disorder*.ti,ab. | 10211 |
| 77 | phobic disorder*.ti,ab. | 428 |
| 78 | phobia*.ti,ab. | 9800 |
| 79 | agoraphobi*.ti,ab. | 3653 |
| 80 | social anxiet*.ti,ab. | 7905 |
| 81 | social phobi*.ti,ab. | 4218 |
| 82 | Anxiety/ep, et, px [Epidemiology, Etiology, Psychology] | 54717 |
| 83 | generali*ed anxiety disorder*.ti,ab. | 10846 |
| 84 | selective mutism.ti,ab. | 272 |
| 85 | 59 or 60 or 61 or 62 or 63 or 64 or 65 or 66 or 67 or 68 or 69 or 70 or 71 or 72 or 73 or 74 or 75 or 76 or 77 or 78 or 79 or 80 or 81 or 82 or 83 or 84 | 467874 |
| 86 | 11 and 27 and 58 and 85 | 4926 |
| 87 | limit 86 to yr="2010 -Current" | 3770 |

## APA PsychInfo

Database(s): **APA PsycInfo**1806 to February Week 4 2024
Search Strategy:

| **#** | **Searches** | **Results** |
| --- | --- | --- |
| 1 | Adolescen*.ab,ti. | 276908 |
| 2 | adolescent psychology/ | 5899 |
| 3 | emerging adulthood/ | 8494 |
| 4 | emerging adult*.ti,ab. | 7450 |
| 5 | young adult*.ti,ab. | 61212 |
| 6 | adolescent mental health/ | 2456 |
| 7 | young adult mental health/ | 2456 |
| 8 | teen*.ti,ab. | 24853 |
| 9 | youth*.ti,ab. | 122708 |
| 10 | young people*.ti,ab. | 38538 |
| 11 | high school student*.ti,ab. | 27380 |
| 12 | secondary school student*.ti,ab. | 5747 |
| 13 | 1 or 2 or 3 or 4 or 5 or 6 or 7 or 8 or 9 or 10 or 11 or 12 | 445677 |
| 14 | exp human sex differences/ | 127932 |
| 15 | sex difference*.ti,ab. | 27634 |
| 16 | gender difference*.ti,ab. | 36833 |
| 17 | gender-sensitiv*.ti,ab. | 1029 |
| 18 | gender-specific*.ti,ab. | 6550 |
| 19 | sex-specific*.ti,ab. | 5504 |
| 20 | gender-related.ti,ab. | 3576 |
| 21 | gender sensitiv*.ti,ab. | 1029 |
| 22 | gender specific*.ti,ab. | 6550 |
| 23 | sex specific*.ti,ab. | 5504 |
| 24 | gender related.ti,ab. | 3576 |
| 25 | genderrelated.ti,ab. | 4 |
| 26 | gender gap*.ti,ab. | 2390 |
| 27 | gender-gap*.ti,ab. | 2390 |
| 28 | gender*.ti,ab. | 260939 |
| 29 | exp gender gap/ | 939 |
| 30 | 14 or 15 or 16 or 17 or 18 or 19 or 20 or 21 or 22 or 23 or 24 or 25 or 26 or 27 or 28 or 29 | 329821 |
| 31 | exp longitudinal studies/ | 17454 |
| 32 | Longitudinal study.ti,ab. | 47640 |
| 33 | Observational study.ti,ab. | 9808 |
| 34 | exp cohort analysis/ | 1717 |
| 35 | Cohort study.ti,ab. | 26940 |
| 36 | Case control study.ti,ab. | 7722 |
| 37 | Time series study.ti,ab. | 180 |
| 38 | exp prospective studies/ | 1341 |
| 39 | Prospective study.ti,ab. | 12722 |
| 40 | exp Retrospective Studies/ | 979 |
| 41 | Retrospective study.ti,ab. | 6827 |
| 42 | follow-up.ti,ab. | 142977 |
| 43 | Cross-sectional study.ti,ab. | 37994 |
| 44 | exp Risk Factors/ | 105946 |
| 45 | risk factor*.ti,ab. | 107925 |
| 46 | Protective factors/ | 8442 |
| 47 | correlat$.ab,ti. | 438737 |
| 48 | exp statistical correlation/ | 11175 |
| 49 | causal factor*.ti,ab. | 2904 |
| 50 | causal analysis/ | 2512 |
| 51 | exp causality/ | 5745 |
| 52 | exp Causal Analysis/ | 8007 |
| 53 | associat$.ab,ti. | 1004207 |
| 54 | at risk populations/ | 41585 |
| 55 | high risk*.ti,ab. | 48685 |
| 56 | at risk*.ti,ab. | 67483 |
| 57 | resource factor*.ti,ab. | 176 |
| 58 | predictor*.ti,ab. | 170486 |
| 59 | determinant*.ti,ab. | 62456 |
| 60 | (protective adj (factor* or characteristic*)).ti,ab. | 19678 |
| 61 | (predictive adj (factor* or characteristic*)).ti,ab. | 2844 |
| 62 | regression*.ab,ti. | 247209 |
| 63 | exp statistical regression/ | 10207 |
| 64 | multivariate analysis.ti,ab. | 14511 |
| 65 | exp Multivariate Analysis/ | 42986 |
| 66 | etiolog*.ti,ab. | 50459 |
| 67 | 31 or 32 or 33 or 34 or 35 or 36 or 37 or 38 or 39 or 40 or 41 or 42 or 43 or 44 or 45 or 46 or 47 or 48 or 49 or 50 or 51 or 52 or 53 or 54 or 55 or 56 or 57 or 58 or 59 or 60 or 61 or 62 or 63 or 64 or 65 or 66 | 1814541 |
| 68 | anxiet*.ti,ab. | 240472 |
| 69 | anxiety disorders/ | 21762 |
| 70 | anxiety/ | 80252 |
| 71 | panic*.ti,ab. | 18174 |
| 72 | exp panic/ | 2348 |
| 73 | panic attack/ | 1003 |
| 74 | worry*.ti,ab. | 13718 |
| 75 | worri*.ti,ab. | 8419 |
| 76 | fear*.ti,ab. | 96988 |
| 77 | psychological stress/ | 9881 |
| 78 | psychological stress*.ti,ab. | 6389 |
| 79 | Psychological distress.ti,ab. | 24459 |
| 80 | separation anxiety/ | 1655 |
| 81 | panic disorder/ | 8050 |
| 82 | phobias/ | 5896 |
| 83 | agoraphobia/ | 2993 |
| 84 | social phobia/ | 5331 |
| 85 | social anxiety/ | 6217 |
| 86 | separation anxiet*.ti,ab. | 2975 |
| 87 | panic disorder*.ti,ab. | 11280 |
| 88 | phobic disorder*.ti,ab. | 493 |
| 89 | phobia*.ti,ab. | 13089 |
| 90 | agoraphobi*.ti,ab. | 5266 |
| 91 | social anxiet*.ti,ab. | 11299 |
| 92 | social phobi*.ti,ab. | 5684 |
| 93 | generali*ed anxiety disorder*.ti,ab. | 9454 |
| 94 | selective mutism.ti,ab. | 518 |
| 95 | fear/ | 20947 |
| 96 | generalized anxiety disorder/ | 3806 |
| 97 | selective mutism/ | 549 |
| 98 | 68 or 69 or 70 or 71 or 72 or 73 or 74 or 75 or 76 or 77 or 78 or 79 or 80 or 81 or 82 or 83 or 84 or 85 or 86 or 87 or 88 or 89 or 90 or 91 or 92 or 93 or 94 or 95 or 96 or 97 | 373978 |
| 99 | 13 and 30 and 67 and 98 | 4426 |
| 100 | limit 99 to yr="2010 -Current" | 3240 |

## Embase

Database(s): **Embase**1974 to 2024 February 28
Search Strategy:

| **#** | **Searches** | **Results** |
| --- | --- | --- |
| 1 | Adolescen*.ab,ti. | 473284 |
| 2 | young adult*.ab,ti. | 165755 |
| 3 | youth.ab,ti. | 112003 |
| 4 | emerging adult*.ab,ti. | 4735 |
| 5 | teen*.ab,ti. | 50025 |
| 6 | young people*.ti,ab. | 54258 |
| 7 | high school student*.ti,ab. | 15119 |
| 8 | secondary school student*.ti,ab. | 3676 |
| 9 | 1 or 2 or 3 or 4 or 5 or 6 or 7 or 8 | 731213 |
| 10 | sex factor/ | 12700 |
| 11 | sex difference*.ti,ab. | 54875 |
| 12 | gender difference*.ti,ab. | 51974 |
| 13 | gender-sensitiv*.ti,ab. | 1359 |
| 14 | gender-specific*.ti,ab. | 20228 |
| 15 | sex-specific*.ti,ab. | 38556 |
| 16 | gender-related.ti,ab. | 8196 |
| 17 | gender sensitiv*.ti,ab. | 1359 |
| 18 | gender specific*.ti,ab. | 20228 |
| 19 | sex specific*.ti,ab. | 38556 |
| 20 | gender related.ti,ab. | 8196 |
| 21 | genderrelated.ti,ab. | 110 |
| 22 | gender gap*.ti,ab. | 2576 |
| 23 | gender-gap*.ti,ab. | 2576 |
| 24 | gender*.ti,ab. | 696888 |
| 25 | 10 or 11 or 12 or 13 or 14 or 15 or 16 or 17 or 18 or 19 or 20 or 21 or 22 or 23 or 24 | 782946 |
| 26 | epidemiology/ | 256446 |
| 27 | longitudinal study/ | 207378 |
| 28 | longitudinal study.ti,ab. | 97566 |
| 29 | observational study/ | 360630 |
| 30 | Observational study.ti,ab. | 209205 |
| 31 | cohort analysis/ | 1124099 |
| 32 | Cohort study.ti,ab. | 447126 |
| 33 | Case control study.ti,ab. | 149303 |
| 34 | case control study/ | 213708 |
| 35 | Time series study.ti,ab. | 2048 |
| 36 | time series analysis/ | 39796 |
| 37 | Prospective study.ti,ab. | 253644 |
| 38 | prospective study/ | 906493 |
| 39 | Retrospective study.ti,ab. | 352871 |
| 40 | retrospective study/ | 1574411 |
| 41 | follow-up.ti,ab. | 1999815 |
| 42 | follow up/ | 2149066 |
| 43 | Cross-sectional study.ti,ab. | 345801 |
| 44 | cross-sectional study/ | 616269 |
| 45 | risk factor/ | 1388588 |
| 46 | risk factor*.ti,ab. | 1166475 |
| 47 | protective factors.ti,ab. | 19944 |
| 48 | correlat$.ab,ti. | 3087615 |
| 49 | correlation analysis/ | 278996 |
| 50 | causal factor*.ti,ab. | 8709 |
| 51 | associat$.ab,ti. | 7931104 |
| 52 | high risk*.ti,ab. | 602791 |
| 53 | at risk*.ti,ab. | 331617 |
| 54 | resource factor*.ti,ab. | 250 |
| 55 | predictor*.ti,ab. | 779948 |
| 56 | determinant*.ti,ab. | 356585 |
| 57 | (protective adj (factor* or characteristic*)).ti,ab. | 39123 |
| 58 | (predictive adj (factor* or characteristic*)).ti,ab. | 59950 |
| 59 | regression analysis/ | 139313 |
| 60 | regression$.ab,ti. | 1589042 |
| 61 | multivariate analysis/ | 174206 |
| 62 | multivariate analysis.ti,ab. | 302271 |
| 63 | 26 or 27 or 28 or 29 or 30 or 31 or 32 or 33 or 34 or 35 or 36 or 37 or 38 or 39 or 40 or 41 or 42 or 43 or 44 or 45 or 46 or 47 or 48 or 49 or 50 or 51 or 52 or 53 or 54 or 55 or 56 or 57 or 58 or 59 or 60 or 61 or 62 | 14766667 |
| 64 | anxiet$.ab,ti. | 386795 |
| 65 | anxiety/ep, et [Epidemiology, Etiology] | 2923 |
| 66 | anxiety disorder/ep, et [Epidemiology, Etiology] | 6693 |
| 67 | generalized anxiety disorder/ep, et [Epidemiology, Etiology] | 762 |
| 68 | panic/ep, et [Epidemiology, Etiology] | 2220 |
| 69 | panic*.ti,ab. | 30434 |
| 70 | worry*.ti,ab. | 27229 |
| 71 | worri*.ti,ab. | 23403 |
| 72 | fear*.ti,ab. | 149690 |
| 73 | mental stress/ep, et [Epidemiology, Etiology] | 5832 |
| 74 | distress syndrome/ep, et [Epidemiology, Etiology] | 1382 |
| 75 | Psychological distress.ti,ab. | 36234 |
| 76 | psychological stress*.ti,ab. | 15423 |
| 77 | separation anxiety/ep, et [Epidemiology, Etiology] | 279 |
| 78 | separation anxiet*.ti,ab. | 2449 |
| 79 | panic disorder*.ti,ab. | 13566 |
| 80 | phobia/ep, et [Epidemiology, Etiology] | 1283 |
| 81 | phobic disorder*.ti,ab. | 549 |
| 82 | phobia*.ti,ab. | 13485 |
| 83 | agoraphobia/ep, et [Epidemiology, Etiology] | 561 |
| 84 | agoraphobi*.ti,ab. | 4877 |
| 85 | social phobia/ep, et [Epidemiology, Etiology] | 956 |
| 86 | social anxiety/ | 1456 |
| 87 | social phobi*.ti,ab. | 5873 |
| 88 | social anxiet*.ti,ab. | 10309 |
| 89 | generali*ed anxiety disorder*.ti,ab. | 15128 |
| 90 | selective mutism/ep, et [Epidemiology, Etiology] | 17 |
| 91 | selective mutism.ti,ab. | 418 |
| 92 | fear/ | 82532 |
| 93 | 64 or 65 or 66 or 67 or 68 or 69 or 70 or 71 or 72 or 73 or 74 or 75 or 76 or 77 or 78 or 79 or 80 or 81 or 82 or 83 or 84 or 85 or 86 or 87 or 88 or 89 or 90 or 91 or 92 | 615081 |
| 94 | 9 and 25 and 63 and 93 | 5363 |
| 95 | limit 94 to yr="2010 -Current" | 4568 |

## Web Of Science

<https://www.webofscience.com/wos/woscc/summary/a35ddd68-04c6-4ff8-977e-a2c522e9447c-cfab84cc/relevance/1>

Abstract search results: 1135

(AB=(Adolescen* OR “Young adult*” OR Youth OR “Emerging adult*” OR Teen* OR “young people” OR “high school student*” OR “high school student*”) AND AB=(“Sex difference*” OR “Gender difference*” OR “Gender-sensitiv*” OR “Gender-specific*” OR “Sex-specific*” OR “Gender-related” OR “Gender sensitiv*” OR “Gender specific*” OR “Sex specific*” OR “Gender related” OR Genderrelated OR Gender*) AND AB=(Anxiet* OR Panic* OR Worry* OR fear* OR “Psychological distress” OR “Psychological stress” OR “Separation anxiety” OR “Phobic disorder*” OR Agoraphobia OR “Social anxiety” OR “Social phobia” OR “generali*ed anxiety disorder*” OR “Selective mutism” ) AND AB=(“Longitudinal study” OR “Observational study” OR “Cohort study” OR “Case control study” OR “Time series study” OR “Prospective study” OR “Retrospective study” OR “Follow-up” OR “Cross-sectional study” OR “Risk factor*” OR Correlat$ OR “Causal factor*” OR Associat$ OR “Birth cohort” OR “High risk” OR “At risk” OR "Resource factor*" OR Predictor* OR Determinant* OR “Protective factor*” OR “Predictive factor” OR Correlat$ OR Regression$ OR “Multivariate analysis” OR Etiolog*)) AND (TASCA==("PSYCHOLOGY DEVELOPMENTAL" OR "PSYCHOLOGY MULTIDISCIPLINARY" OR "PSYCHOLOGY" OR "FAMILY STUDIES" OR "SOCIAL SCIENCES INTERDISCIPLINARY" OR "PSYCHOLOGY SOCIAL" OR "PSYCHIATRY" OR "SOCIOLOGY" OR "PSYCHOLOGY EDUCATIONAL" OR "PSYCHOLOGY APPLIED" OR "SOCIAL SCIENCES BIOMEDICAL") AND SJ==("PSYCHOLOGY" OR "PSYCHIATRY" OR "FAMILY STUDIES" OR "SOCIAL SCIENCES OTHER TOPICS" OR "SOCIAL WORK" OR "SOCIOLOGY" OR "WOMEN S STUDIES")) and 2024 or 2023 or 2022 or 2021 or 2020 or 2019 or 2018 or 2017 or 2016 or 2015 or 2014 or 2013 or 2012 or 2011 or 2010 (Publication Years) and Psychiatry or Psychology Developmental or Psychology Multidisciplinary or Psychology or Family Studies or Social Sciences Interdisciplinary or Psychology Social or Psychology Applied or Psychology Educational or Sociology or Anthropology or Developmental Biology or Women S Studies or Social Issues or Education Educational Research or Ethnic Studies (Web of Science Categories) and Psychology or Psychiatry or Social Work or Social Sciences Other Topics or Family Studies or Sociology or Education Educational Research or Ethnic Studies or Women S Studies or Anthropology or Social Issues (Research Areas)

<https://www.webofscience.com/wos/woscc/summary/ac42e428-4391-4d63-a1f1-560144c6fc73-cfabb7cc/relevance/1>

Title search results: 7

**(TI=(Adolescen* OR “Young adult*” OR Youth OR “Emerging adult*” OR Teen* OR “young people” OR “high school student*” OR “high school student*”) AND TI=(“Sex difference*” OR “Gender difference*” OR “Gender-sensitiv*” OR “Gender-specific*” OR “Sex-specific*” OR “Gender-related” OR “Gender sensitiv*” OR “Gender specific*” OR “Sex specific*” OR “Gender related” OR generelated OR Gender*) AND TI=(Anxiet* OR Panic* OR Worry* OR fear* OR “Psychological distress” OR “Psychological stress” OR “Separation anxiety” OR “Phobic disorder*” OR Agoraphobia OR “Social anxiety” OR “Social phobia” OR “generali*ed anxiety disorder*” OR “Selective mutism” ) AND TI=(“Longitudinal study” OR “Observational study” OR “Cohort study” OR “Case control study” OR “Time series study” OR “Prospective study” OR “Retrospective study” OR “Follow-up” OR “Cross-sectional study” OR “Risk factor*” OR Correlat$ OR “Causal factor*” OR Associat$ OR “Birth cohort” OR “High risk” OR “At risk” OR "Resource factor*" OR Predictor* OR Determinant* OR “Protective factor*” OR “Predictive factor” OR Correlat$ OR Regression$ OR “Multivariate analysis” OR Etiolog*))** AND (TASCA==("PSYCHOLOGY DEVELOPMENTAL" OR "PSYCHOLOGY MULTIDISCIPLINARY" OR "PSYCHOLOGY" OR "FAMILY STUDIES" OR "SOCIAL SCIENCES INTERDISCIPLINARY" OR "PSYCHOLOGY SOCIAL" OR "PSYCHIATRY" OR "SOCIOLOGY" OR "PSYCHOLOGY EDUCATIONAL" OR "PSYCHOLOGY APPLIED" OR "SOCIAL SCIENCES BIOMEDICAL") AND SJ==("PSYCHOLOGY" OR "PSYCHIATRY" OR "FAMILY STUDIES" OR "SOCIAL SCIENCES OTHER TOPICS" OR "SOCIAL WORK" OR "SOCIOLOGY" OR "WOMEN S STUDIES")) and 2024 or 2023 or 2022 or 2021 or 2020 or 2019 or 2018 or 2017 or 2016 or 2015 or 2014 or 2013 or 2012 or 2011 or 2010 (Publication Years) and Psychiatry or Psychology Developmental or Psychology Multidisciplinary or Psychology or Family Studies or Social Sciences Interdisciplinary or Psychology Social or Psychology Applied or Psychology Educational or Sociology or Anthropology or Developmental Biology or Women S Studies or Social Issues or Education Educational Research or Ethnic Studies (Web of Science Categories) and Psychology or Psychiatry or Social Work or Social Sciences Other Topics or Family Studies or Sociology or Education Educational Research or Ethnic Studies or Women S Studies or Anthropology or Social Issues (Research Areas)

## Sociological abstracts: ERIC, International Bibliography of the Social Sciences (IBSS)‎

Abstract search results: 215

abstract(Adolescen* OR "Young adult*" OR Youth OR "Emerging adult*" OR Teen* OR "young people" OR "high school student*" OR "high school student*") AND abstract("Sex difference*" OR "Gender difference*" OR "Gender-sensitiv*" OR "Gender-specific*" OR "Sex-specific*" OR "Gender-related" OR "Gender sensitiv*" OR "Gender specific*" OR "Sex specific*" OR "Gender related" OR Genderrelated OR Gender*) AND abstract(Anxiet* OR Panic* OR Worry* OR fear* OR "Psychological distress" OR "Psychological stress" OR "Separation anxiety" OR "Phobic disorder*" OR Agoraphobia OR "Social anxiety" OR "Social phobia" OR "generali*ed anxiety disorder*" OR "Selective mutism") AND abstract("Longitudinal study" OR "Observational study" OR "Cohort study" OR "Case control study" OR "Time series study" OR "Prospective study" OR "Retrospective study" OR "Follow-up" OR "Cross-sectional study" OR "Risk factor*" OR Correlat OR "Causal factor*" OR Associat OR "Birth cohort" OR "High risk" OR "At risk" OR "Resource factor*" OR Predictor* OR Determinant* OR "Protective factor*" OR "Predictive factor" OR Correlat OR Regression OR "Multivariate analysis" OR Etiolog*)

Title search results: 1

title(Adolescen* OR "Young adult*" OR Youth OR "Emerging adult*" OR Teen* OR "young people" OR "high school student*" OR "high school student*") AND title("Sex difference*" OR "Gender difference*" OR "Gender-sensitiv*" OR "Gender-specific*" OR "Sex-specific*" OR "Gender-related" OR "Gender sensitiv*" OR "Gender specific*" OR "Sex specific*" OR "Gender related" OR Genderrelated OR Gender*) AND title(Anxiet* OR Panic* OR Worry* OR fear* OR "Psychological distress" OR "Psychological stress" OR "Separation anxiety" OR "Phobic disorder*" OR Agoraphobia OR "Social anxiety" OR "Social phobia" OR "generali*ed anxiety disorder*" OR "Selective mutism") AND title("Longitudinal study" OR "Observational study" OR "Cohort study" OR "Case control study" OR "Time series study" OR "Prospective study" OR "Retrospective study" OR "Follow-up" OR "Cross-sectional study" OR "Risk factor*" OR Correlat OR "Causal factor*" OR Associat OR "Birth cohort" OR "High risk" OR "At risk" OR "Resource factor*" OR Predictor* OR Determinant* OR "Protective factor*" OR "Predictive factor" OR Correlat OR Regression OR "Multivariate analysis" OR Etiolog*)

## Scopus

Abstract search results: 1125

( ABS ( adolescen* OR "Young adult*" OR youth OR "Emerging adult*" OR teen* OR "young people" OR "high school student*" OR "high school student*" ) AND ABS ( "Sex difference*" OR "Gender difference*" OR "Gender-sensitiv*" OR "Gender-specific*" OR "Sex-specific*" OR "Gender-related" OR "Gender sensitiv*" OR "Gender specific*" OR "Sex specific*" OR "Gender related" OR genderrelated OR gender* ) AND ABS ( anxiet* OR panic* OR worry* OR fear* OR "Psychological distress" OR "Psychological stress" OR "Separation anxiety" OR "Phobic disorder*" OR agoraphobia OR "Social anxiety" OR "Social phobia" OR "generali*ed anxiety disorder*" OR "Selective mutism" ) AND ABS ( "Longitudinal study" OR "Observational study" OR "Cohort study" OR "Case control study" OR "Time series study" OR "Prospective study" OR "Retrospective study" OR "Follow-up" OR "Cross-sectional study" OR "Risk factor*" OR correlat$ OR "Causal factor*" OR associat$ OR "Birth cohort" OR "High risk" OR "At risk" OR "Resource factor*" OR predictor* OR determinant* OR "Protective factor*" OR "Predictive factor" OR correlat$ OR regression$ OR "Multivariate analysis" OR etiolog* ) ) AND PUBYEAR > 2009 AND ( LIMIT-TO ( SUBJAREA , "PSYC" ) OR LIMIT-TO ( SUBJAREA , "SOCI" ) )

Title search results: 7

( TITLE ( adolescen* OR "Young adult*" OR youth OR "Emerging adult*" OR teen* OR "young people" OR “high school student*" OR "high school student*") AND TITLE ( "Sex difference*" OR "Gender difference*" OR "Gender-sensitiv*" OR "Gender-specific*" OR "Sex-specific*" OR "Gender-related" OR "Gender sensitiv*" OR "Gender specific*" OR "Sex specific*" OR "Gender related" OR genderrelated OR gender* ) AND TITLE ( anxiet* OR panic* OR worry* OR fear* OR "Psychological distress" OR "Psychological stress" OR "Separation anxiety" OR "Phobic disorder*" OR agoraphobia OR "Social anxiety" OR "Social phobia" OR "generali*ed anxiety disorder*" OR "Selective mutism" ) AND TITLE ( "Longitudinal study" OR "Observational study" OR "Cohort study" OR "Case control study" OR "Time series study" OR "Prospective study" OR "Retrospective study" OR "Follow-up" OR "Cross-sectional study" OR "Risk factor*" OR correlat$ OR "Causal factor*" OR associat$ OR "Birth cohort" OR "High risk" OR "At risk" OR "Resource factor*" OR predictor* OR determinant* OR "Protective factor*" OR "Predictive factor" OR correlat$ OR regression$ OR "Multivariate analysis" OR etiolog* ) ) AND PUBYEAR > 2009 AND ( LIMIT-TO ( SUBJAREA , "PSYC" ) OR LIMIT-TO ( SUBJAREA , "SOCI" ) )

# Update search: 4^th^ July 2025

## Medline

Database(s): **Ovid MEDLINE(R)**1946 to present
Search Strategy:

| **#** | **Searches** | **Results** |
| --- | --- | --- |
| 1 | Adolescent/px [Psychology] | 298 |
| 2 | Adolescen*.ab,ti. | 389227 |
| 3 | Young Adult/px [Psychology] | 58 |
| 4 | young adult*.ab,ti. | 135063 |
| 5 | youth.ab,ti. | 98969 |
| 6 | emerging adult*.ab,ti. | 4749 |
| 7 | teen*.ab,ti. | 37764 |
| 8 | young people*.ti,ab. | 43039 |
| 9 | high school student*.ti,ab. | 13777 |
| 10 | secondary school student*.ti,ab. | 3688 |
| 11 | 1 or 2 or 3 or 4 or 5 or 6 or 7 or 8 or 9 or 10 | 604547 |
| 12 | sex difference*.ti,ab. | 48964 |
| 13 | gender difference*.ti,ab. | 41358 |
| 14 | Sex Factors/ | 289390 |
| 15 | gender-sensitiv*.ti,ab. | 1472 |
| 16 | gender-specific*.ti,ab. | 16335 |
| 17 | sex-specific*.ti,ab. | 34870 |
| 18 | gender-related.ti,ab. | 6638 |
| 19 | gender sensitiv*.ti,ab. | 1472 |
| 20 | gender specific*.ti,ab. | 16335 |
| 21 | sex specific*.ti,ab. | 34870 |
| 22 | gender related.ti,ab. | 6638 |
| 23 | genderrelated.ti,ab. | 5 |
| 24 | gender gap*.ti,ab. | 2597 |
| 25 | gender-gap*.ti,ab. | 2597 |
| 26 | gender*.ti,ab. | 474838 |
| 27 | 12 or 13 or 14 or 15 or 16 or 17 or 18 or 19 or 20 or 21 or 22 or 23 or 24 or 25 or 26 | 748740 |
| 28 | exp epidemiologic studies/ | 3495985 |
| 29 | Longitudinal study.ti,ab. | 84227 |
| 30 | Observational study.ti,ab. | 147434 |
| 31 | Cohort study.ti,ab. | 355122 |
| 32 | Case control study.ti,ab. | 121369 |
| 33 | Time series study.ti,ab. | 2132 |
| 34 | Prospective study.ti,ab. | 174796 |
| 35 | Retrospective study.ti,ab. | 253224 |
| 36 | Follow-up.ti,ab. | 1342887 |
| 37 | Cross-sectional study.ti,ab. | 309966 |
| 38 | risk factors/ | 1040126 |
| 39 | risk factor*.ti,ab. | 860362 |
| 40 | protective factors/ | 6981 |
| 41 | correlat$.ab,ti. | 2508843 |
| 42 | causal factor*.ti,ab. | 6990 |
| 43 | associat$.ab,ti. | 6271872 |
| 44 | high risk.ti,ab. | 416353 |
| 45 | at risk.ti,ab. | 241250 |
| 46 | "resource factor*".ti,ab. | 218 |
| 47 | predictor*.ti,ab. | 565137 |
| 48 | determinant*.ti,ab. | 313174 |
| 49 | (protective adj (factor* or characteristic*)).ti,ab. | 35553 |
| 50 | (predictive adj (factor* or characteristic*)).ti,ab. | 40515 |
| 51 | "correlation of data"/ | 2871 |
| 52 | correlat$.ab,ti. | 2508843 |
| 53 | exp Regression Analysis/ | 476113 |
| 54 | regression$.ab,ti. | 1256379 |
| 55 | multivariate analysis/ | 133487 |
| 56 | multivariate analysis.ti,ab. | 204702 |
| 57 | etiolog*.ti,ab. | 343450 |
| 58 | 28 or 29 or 30 or 31 or 32 or 33 or 34 or 35 or 36 or 37 or 38 or 39 or 40 or 41 or 42 or 43 or 44 or 45 or 46 or 47 or 48 or 49 or 50 or 51 or 52 or 53 or 54 or 55 or 56 or 57 | 11784602 |
| 59 | anxiet$.ab,ti. | 303401 |
| 60 | panic*.ti,ab. | 25414 |
| 61 | worri*.ti,ab. | 17065 |
| 62 | worry*.ti,ab. | 20542 |
| 63 | fear*.ti,ab. | 119986 |
| 64 | exp Fear/et, px [Etiology, Psychology] | 8410 |
| 65 | Stress, Psychological/ep, et, px [Epidemiology, Etiology, Psychology] | 56324 |
| 66 | Psychological Distress/ | 6176 |
| 67 | Psychological distress.ti,ab. | 32328 |
| 68 | psychological stress*.ti,ab. | 12391 |
| 69 | Anxiety Disorders/ep, et, px [Epidemiology, Etiology, Psychology] | 23618 |
| 70 | Anxiety, Separation/ep, et, px [Epidemiology, Etiology, Psychology] | 1188 |
| 71 | Panic Disorder/ep, et, px [Epidemiology, Etiology, Psychology] | 4332 |
| 72 | Phobic Disorders/ep, et, px [Epidemiology, Etiology, Psychology] | 6415 |
| 73 | Agoraphobia/ep, et, px [Epidemiology, Etiology, Psychology] | 1720 |
| 74 | Phobia, Social/ep, et, px [Epidemiology, Etiology, Psychology] | 792 |
| 75 | separation anxiet*.ti,ab. | 1901 |
| 76 | panic disorder*.ti,ab. | 10573 |
| 77 | phobic disorder*.ti,ab. | 436 |
| 78 | phobia*.ti,ab. | 10303 |
| 79 | agoraphobi*.ti,ab. | 3750 |
| 80 | social anxiet*.ti,ab. | 8849 |
| 81 | social phobi*.ti,ab. | 4378 |
| 82 | Anxiety/ep, et, px [Epidemiology, Etiology, Psychology] | 62013 |
| 83 | generali*ed anxiety disorder*.ti,ab. | 12797 |
| 84 | selective mutism.ti,ab. | 302 |
| 85 | 59 or 60 or 62 or 64 or 65 or 66 or 67 or 68 or 69 or 70 or 71 or 72 or 73 or 74 or 75 or 76 or 77 or 78 or 79 or 80 or 81 or 82 or 83 or 84 | 428208 |
| 86 | 11 and 27 and 58 and 85 | 5217 |
| 87 | limit 86 to yr="2010 -Current" | 4178 |
| 88 | limit 87 to dt=20240229-20250704 [February 29th, 2024 to July 4th, 2025] | 595 |

## APA PsycInfo

Database(s): **APA PsycInfo**1806 to June 2025 Week 5
Search Strategy:

| **#** | **Searches** | **Results** |
| --- | --- | --- |
| 1 | Adolescen*.ab,ti. | 294179 |
| 2 | adolescent psychology/ | 6596 |
| 3 | emerging adulthood/ | 11556 |
| 4 | emerging adult*.ti,ab. | 8608 |
| 5 | young adult*.ti,ab. | 66527 |
| 6 | adolescent mental health/ | 4146 |
| 7 | young adult mental health/ | 4146 |
| 8 | teen*.ti,ab. | 25987 |
| 9 | youth*.ti,ab. | 132120 |
| 10 | young people*.ti,ab. | 42195 |
| 11 | high school student*.ti,ab. | 28887 |
| 12 | secondary school student*.ti,ab. | 6213 |
| 13 | 1 or 2 or 3 or 4 or 5 or 6 or 7 or 8 or 9 or 10 or 11 or 12 | 476289 |
| 14 | exp human sex differences/ | 133754 |
| 15 | sex difference*.ti,ab. | 29003 |
| 16 | gender difference*.ti,ab. | 38740 |
| 17 | gender-sensitiv*.ti,ab. | 1167 |
| 18 | gender-specific*.ti,ab. | 7040 |
| 19 | sex-specific*.ti,ab. | 6237 |
| 20 | gender-related.ti,ab. | 3817 |
| 21 | gender sensitiv*.ti,ab. | 1167 |
| 22 | gender specific*.ti,ab. | 7040 |
| 23 | sex specific*.ti,ab. | 6237 |
| 24 | gender related.ti,ab. | 3817 |
| 25 | genderrelated.ti,ab. | 8 |
| 26 | gender gap*.ti,ab. | 2648 |
| 27 | gender-gap*.ti,ab. | 2648 |
| 28 | gender*.ti,ab. | 280785 |
| 29 | exp gender gap/ | 1105 |
| 30 | 14 or 15 or 16 or 17 or 18 or 19 or 20 or 21 or 22 or 23 or 24 or 25 or 26 or 27 or 28 or 29 | 352606 |
| 31 | exp longitudinal studies/ | 17557 |
| 32 | Longitudinal study.ti,ab. | 51459 |
| 33 | Observational study.ti,ab. | 11046 |
| 34 | exp cohort analysis/ | 1789 |
| 35 | Cohort study.ti,ab. | 30765 |
| 36 | Case control study.ti,ab. | 8252 |
| 37 | Time series study.ti,ab. | 207 |
| 38 | exp prospective studies/ | 1373 |
| 39 | Prospective study.ti,ab. | 13283 |
| 40 | exp Retrospective Studies/ | 1076 |
| 41 | Retrospective study.ti,ab. | 7517 |
| 42 | follow-up.ti,ab. | 152877 |
| 43 | Cross-sectional study.ti,ab. | 44568 |
| 44 | exp Risk Factors/ | 109993 |
| 45 | risk factor*.ti,ab. | 116583 |
| 46 | Protective factors/ | 9627 |
| 47 | correlat$.ab,ti. | 466970 |
| 48 | exp statistical correlation/ | 11445 |
| 49 | causal factor*.ti,ab. | 3031 |
| 50 | causal analysis/ | 2670 |
| 51 | exp causality/ | 6234 |
| 52 | exp Causal Analysis/ | 8641 |
| 53 | associat$.ab,ti. | 1080134 |
| 54 | at risk populations/ | 43157 |
| 55 | high risk*.ti,ab. | 52353 |
| 56 | at risk*.ti,ab. | 72017 |
| 57 | resource factor*.ti,ab. | 186 |
| 58 | predictor*.ti,ab. | 182099 |
| 59 | determinant*.ti,ab. | 66948 |
| 60 | (protective adj (factor* or characteristic*)).ti,ab. | 22177 |
| 61 | (predictive adj (factor* or characteristic*)).ti,ab. | 3115 |
| 62 | regression*.ab,ti. | 272087 |
| 63 | exp statistical regression/ | 10561 |
| 64 | multivariate analysis.ti,ab. | 15213 |
| 65 | exp Multivariate Analysis/ | 45653 |
| 66 | etiolog*.ti,ab. | 52237 |
| 67 | 31 or 32 or 33 or 34 or 35 or 36 or 37 or 38 or 39 or 40 or 41 or 42 or 43 or 44 or 45 or 46 or 47 or 48 or 49 or 50 or 51 or 52 or 53 or 54 or 55 or 56 or 57 or 58 or 59 or 60 or 61 or 62 or 63 or 64 or 65 or 66 | 1935378 |
| 68 | anxiet*.ti,ab. | 259693 |
| 69 | anxiety disorders/ | 23269 |
| 70 | anxiety/ | 87816 |
| 71 | panic*.ti,ab. | 18647 |
| 72 | exp panic/ | 2405 |
| 73 | panic attack/ | 1015 |
| 74 | worry*.ti,ab. | 14818 |
| 75 | worri*.ti,ab. | 9053 |
| 76 | fear*.ti,ab. | 102968 |
| 77 | psychological stress/ | 10204 |
| 78 | psychological stress*.ti,ab. | 6822 |
| 79 | Psychological distress.ti,ab. | 27092 |
| 80 | separation anxiety/ | 1707 |
| 81 | panic disorder/ | 8160 |
| 82 | phobias/ | 6154 |
| 83 | agoraphobia/ | 3022 |
| 84 | social phobia/ | 5505 |
| 85 | social anxiety/ | 6762 |
| 86 | separation anxiet*.ti,ab. | 3065 |
| 87 | panic disorder*.ti,ab. | 11514 |
| 88 | phobic disorder*.ti,ab. | 499 |
| 89 | phobia*.ti,ab. | 13397 |
| 90 | agoraphobi*.ti,ab. | 5352 |
| 91 | social anxiet*.ti,ab. | 12188 |
| 92 | social phobi*.ti,ab. | 5794 |
| 93 | generali*ed anxiety disorder*.ti,ab. | 10465 |
| 94 | selective mutism.ti,ab. | 536 |
| 95 | fear/ | 22229 |
| 96 | generalized anxiety disorder/ | 4196 |
| 97 | selective mutism/ | 574 |
| 98 | 68 or 69 or 70 or 71 or 72 or 73 or 74 or 77 or 78 or 79 or 80 or 81 or 82 or 83 or 84 or 85 or 86 or 87 or 88 or 89 or 90 or 91 or 92 or 93 or 94 or 95 or 96 or 97 | 337646 |
| 99 | 13 and 30 and 67 and 98 | 4576 |
| 100 | limit 99 to yr="2010 -Current" | 3521 |
| 101 | limit 100 to up=20240229-20250704 | 520 |

## Embase

Database(s): **Embase**1974 to 2025 July 03
Search Strategy:

| **#** | **Searches** | **Results** |
| --- | --- | --- |
| 1 | Adolescen*.ab,ti. | 528042 |
| 2 | young adult*.ab,ti. | 186603 |
| 3 | youth.ab,ti. | 127648 |
| 4 | emerging adult*.ab,ti. | 5593 |
| 5 | teen*.ab,ti. | 54725 |
| 6 | young people*.ti,ab. | 61831 |
| 7 | high school student*.ti,ab. | 16581 |
| 8 | secondary school student*.ti,ab. | 4135 |
| 9 | 1 or 2 or 3 or 4 or 5 or 6 or 7 or 8 | 816219 |
| 10 | sex factor/ | 15688 |
| 11 | sex difference*.ti,ab. | 61622 |
| 12 | gender difference*.ti,ab. | 56267 |
| 13 | gender-sensitiv*.ti,ab. | 1694 |
| 14 | gender-specific*.ti,ab. | 22568 |
| 15 | sex-specific*.ti,ab. | 45102 |
| 16 | gender-related.ti,ab. | 8932 |
| 17 | gender sensitiv*.ti,ab. | 1694 |
| 18 | gender specific*.ti,ab. | 22568 |
| 19 | sex specific*.ti,ab. | 45102 |
| 20 | gender related.ti,ab. | 8932 |
| 21 | genderrelated.ti,ab. | 116 |
| 22 | gender gap*.ti,ab. | 2980 |
| 23 | gender-gap*.ti,ab. | 2980 |
| 24 | gender*.ti,ab. | 773123 |
| 25 | 10 or 11 or 12 or 13 or 14 or 15 or 16 or 17 or 18 or 19 or 20 or 21 or 22 or 23 or 24 | 870932 |
| 26 | epidemiology/ | 317591 |
| 27 | longitudinal study/ | 248457 |
| 28 | longitudinal study.ti,ab. | 109963 |
| 29 | observational study/ | 471385 |
| 30 | Observational study.ti,ab. | 261209 |
| 31 | cohort analysis/ | 1397028 |
| 32 | Cohort study.ti,ab. | 532869 |
| 33 | Case control study.ti,ab. | 163730 |
| 34 | case control study/ | 238257 |
| 35 | Time series study.ti,ab. | 2417 |
| 36 | time series analysis/ | 46700 |
| 37 | Prospective study.ti,ab. | 284080 |
| 38 | prospective study/ | 992619 |
| 39 | Retrospective study.ti,ab. | 408084 |
| 40 | retrospective study/ | 1848904 |
| 41 | follow-up.ti,ab. | 2253148 |
| 42 | follow up/ | 2465563 |
| 43 | Cross-sectional study.ti,ab. | 409630 |
| 44 | cross-sectional study/ | 741216 |
| 45 | risk factor/ | 1558253 |
| 46 | risk factor*.ti,ab. | 1302677 |
| 47 | protective factors.ti,ab. | 23534 |
| 48 | correlat$.ab,ti. | 3364802 |
| 49 | correlation analysis/ | 303013 |
| 50 | causal factor*.ti,ab. | 9357 |
| 51 | associat$.ab,ti. | 8730428 |
| 52 | high risk*.ti,ab. | 686526 |
| 53 | at risk*.ti,ab. | 371739 |
| 54 | resource factor*.ti,ab. | 274 |
| 55 | predictor*.ti,ab. | 863578 |
| 56 | determinant*.ti,ab. | 389239 |
| 57 | (protective adj (factor* or characteristic*)).ti,ab. | 46061 |
| 58 | (predictive adj (factor* or characteristic*)).ti,ab. | 66912 |
| 59 | regression analysis/ | 143608 |
| 60 | regression$.ab,ti. | 1791800 |
| 61 | multivariate analysis/ | 182695 |
| 62 | multivariate analysis.ti,ab. | 323825 |
| 63 | 26 or 27 or 28 or 29 or 30 or 31 or 32 or 33 or 34 or 35 or 36 or 37 or 38 or 39 or 40 or 41 or 42 or 43 or 44 or 45 or 46 or 47 or 48 or 49 or 50 or 51 or 52 or 53 or 54 or 55 or 56 or 57 or 58 or 59 or 60 or 61 or 62 | 16309793 |
| 64 | anxiet$.ab,ti. | 449767 |
| 65 | anxiety/ep, et [Epidemiology, Etiology] | 2869 |
| 66 | anxiety disorder/ep, et [Epidemiology, Etiology] | 7339 |
| 67 | generalized anxiety disorder/ep, et [Epidemiology, Etiology] | 791 |
| 68 | panic/ep, et [Epidemiology, Etiology] | 2265 |
| 69 | panic*.ti,ab. | 32584 |
| 70 | worry*.ti,ab. | 30592 |
| 71 | worri*.ti,ab. | 25964 |
| 72 | fear*.ti,ab. | 167681 |
| 73 | mental stress/ep, et [Epidemiology, Etiology] | 5780 |
| 74 | distress syndrome/ep, et [Epidemiology, Etiology] | 1424 |
| 75 | Psychological distress.ti,ab. | 42314 |
| 76 | psychological stress*.ti,ab. | 17583 |
| 77 | separation anxiety/ep, et [Epidemiology, Etiology] | 290 |
| 78 | separation anxiet*.ti,ab. | 2677 |
| 79 | panic disorder*.ti,ab. | 14270 |
| 80 | phobia/ep, et [Epidemiology, Etiology] | 1344 |
| 81 | phobic disorder*.ti,ab. | 569 |
| 82 | phobia*.ti,ab. | 14527 |
| 83 | agoraphobia/ep, et [Epidemiology, Etiology] | 563 |
| 84 | agoraphobi*.ti,ab. | 5093 |
| 85 | social phobia/ep, et [Epidemiology, Etiology] | 976 |
| 86 | social anxiety/ | 2496 |
| 87 | social phobi*.ti,ab. | 6202 |
| 88 | social anxiet*.ti,ab. | 11731 |
| 89 | generali*ed anxiety disorder*.ti,ab. | 18111 |
| 90 | selective mutism/ep, et [Epidemiology, Etiology] | 17 |
| 91 | selective mutism.ti,ab. | 456 |
| 92 | fear/ | 92823 |
| 93 | 64 or 65 or 66 or 67 or 68 or 69 or 70 or 71 or 72 or 73 or 74 or 75 or 76 or 77 or 78 or 79 or 80 or 81 or 82 or 83 or 84 or 85 or 86 or 87 or 88 or 89 or 90 or 91 or 92 | 701894 |
| 94 | 9 and 25 and 63 and 93 | 6357 |
| 95 | limit 94 to yr="2010 -Current" | 5560 |
| 96 | limit 95 to dd=20240229-20250704 [February 29th, 2024 to July 4th, 2025] | 976 |

## Web Of Science

<https://www.webofscience.com/wos/woscc/summary/09d7fb80-2e8e-409e-88a9-b6dc608d7d3e-016cb3b577/date-descending/1>

Abstract search results: 144

(AB=(Adolescen* OR “Young adult*” OR Youth OR “Emerging adult*” OR Teen* OR “young people” OR “high school student*” OR “high school student*”) AND AB=(“Sex difference*” OR “Gender difference*” OR “Gender-sensitiv*” OR “Gender-specific*” OR “Sex-specific*” OR “Gender-related” OR “Gender sensitiv*” OR “Gender specific*” OR “Sex specific*” OR “Gender related” OR Genderrelated OR Gender*) AND AB=(Anxiet* OR Panic* OR Worry* OR fear* OR “Psychological distress” OR “Psychological stress” OR “Separation anxiety” OR “Phobic disorder*” OR Agoraphobia OR “Social anxiety” OR “Social phobia” OR “generali*ed anxiety disorder*” OR “Selective mutism” ) AND AB=(“Longitudinal study” OR “Observational study” OR “Cohort study” OR “Case control study” OR “Time series study” OR “Prospective study” OR “Retrospective study” OR “Follow-up” OR “Cross-sectional study” OR “Risk factor*” OR Correlat$ OR “Causal factor*” OR Associat$ OR “Birth cohort” OR “High risk” OR “At risk” OR "Resource factor*" OR Predictor* OR Determinant* OR “Protective factor*” OR “Predictive factor” OR Correlat$ OR Regression$ OR “Multivariate analysis” OR Etiolog*)) AND (TASCA==("PSYCHOLOGY DEVELOPMENTAL" OR "PSYCHOLOGY MULTIDISCIPLINARY" OR "PSYCHOLOGY" OR "FAMILY STUDIES" OR "SOCIAL SCIENCES INTERDISCIPLINARY" OR "PSYCHOLOGY SOCIAL" OR "PSYCHIATRY" OR "SOCIOLOGY" OR "PSYCHOLOGY EDUCATIONAL" OR "PSYCHOLOGY APPLIED" OR "SOCIAL SCIENCES BIOMEDICAL") AND SJ==("PSYCHOLOGY" OR "PSYCHIATRY" OR "FAMILY STUDIES" OR "SOCIAL SCIENCES OTHER TOPICS" OR "SOCIAL WORK" OR "SOCIOLOGY" OR "WOMENS STUDIES"))

<https://www.webofscience.com/wos/woscc/summary/ac42e428-4391-4d63-a1f1-560144c6fc73-cfabb7cc/relevance/1>

Title search results: 2

(TI=(Adolescen* OR “Young adult*” OR Youth OR “Emerging adult*” OR Teen* OR “young people” OR “high school student*” OR “high school student*”) AND TI=(“Sex difference*” OR “Gender difference*” OR “Gender-sensitiv*” OR “Gender-specific*” OR “Sex-specific*” OR “Gender-related” OR “Gender sensitiv*” OR “Gender specific*” OR “Sex specific*” OR “Gender related” OR generelated OR Gender*) AND TI=(Anxiet* OR Panic* OR Worry* OR fear* OR “Psychological distress” OR “Psychological stress” OR “Separation anxiety” OR “Phobic disorder*” OR Agoraphobia OR “Social anxiety” OR “Social phobia” OR “generali*ed anxiety disorder*” OR “Selective mutism” ) AND TI=(“Longitudinal study” OR “Observational study” OR “Cohort study” OR “Case control study” OR “Time series study” OR “Prospective study” OR “Retrospective study” OR “Follow-up” OR “Cross-sectional study” OR “Risk factor*” OR Correlat$ OR “Causal factor*” OR Associat$ OR “Birth cohort” OR “High risk” OR “At risk” OR "Resource factor*" OR Predictor* OR Determinant* OR “Protective factor*” OR “Predictive factor” OR Correlat$ OR Regression$ OR “Multivariate analysis” OR Etiolog*)) AND (TASCA==("PSYCHOLOGY DEVELOPMENTAL" OR "PSYCHOLOGY MULTIDISCIPLINARY" OR "PSYCHOLOGY" OR "FAMILY STUDIES" OR "SOCIAL SCIENCES INTERDISCIPLINARY" OR "PSYCHOLOGY SOCIAL" OR "PSYCHIATRY" OR "SOCIOLOGY" OR "PSYCHOLOGY EDUCATIONAL" OR "PSYCHOLOGY APPLIED" OR "SOCIAL SCIENCES BIOMEDICAL") AND SJ==("PSYCHOLOGY" OR "PSYCHIATRY" OR "FAMILY STUDIES" OR "SOCIAL SCIENCES OTHER TOPICS" OR "SOCIAL WORK" OR "SOCIOLOGY" OR "WOMEN S STUDIES")) and 2024 or 2025 (Publication Years) and Psychiatry or Psychology Developmental or Psychology Multidisciplinary or Psychology or Family Studies or Social Sciences Interdisciplinary or Psychology Social or Psychology Applied or Psychology Educational or Sociology or Anthropology or Developmental Biology or Women S Studies or Social Issues or Education Educational Research or Ethnic Studies (Web of Science Categories) and Psychology or Psychiatry or Social Work or Social Sciences Other Topics or Family Studies or Sociology or Education Educational Research or Ethnic Studies or Women S Studies or Anthropology or Social Issues (Research Areas)

## Sociological abstracts: ERIC, International Bibliography of the Social Sciences (IBSS)‎

Abstract search results: 3

abstract(Adolescen* OR "Young adult*" OR Youth OR "Emerging adult*" OR Teen* OR "young people" OR "high school student*" OR "high school student*") AND abstract("Sex difference*" OR "Gender difference*" OR "Gender-sensitiv*" OR "Gender-specific*" OR "Sex-specific*" OR "Gender-related" OR "Gender sensitiv*" OR "Gender specific*" OR "Sex specific*" OR "Gender related" OR Genderrelated OR Gender*) AND abstract(Anxiet* OR Panic* OR Worry* OR fear* OR "Psychological distress" OR "Psychological stress" OR "Separation anxiety" OR "Phobic disorder*" OR Agoraphobia OR "Social anxiety" OR "Social phobia" OR "generali*ed anxiety disorder*" OR "Selective mutism") AND abstract("Longitudinal study" OR "Observational study" OR "Cohort study" OR "Case control study" OR "Time series study" OR "Prospective study" OR "Retrospective study" OR "Follow-up" OR "Cross-sectional study" OR "Risk factor*" OR Correlat OR "Causal factor*" OR Associat OR "Birth cohort" OR "High risk" OR "At risk" OR "Resource factor*" OR Predictor* OR Determinant* OR "Protective factor*" OR "Predictive factor" OR Correlat OR Regression OR "Multivariate analysis" OR Etiolog*)

Title search results: 0

title(Adolescen* OR "Young adult*" OR Youth OR "Emerging adult*" OR Teen* OR "young people" OR "high school student*" OR "high school student*") AND title("Sex difference*" OR "Gender difference*" OR "Gender-sensitiv*" OR "Gender-specific*" OR "Sex-specific*" OR "Gender-related" OR "Gender sensitiv*" OR "Gender specific*" OR "Sex specific*" OR "Gender related" OR Genderrelated OR Gender*) AND title(Anxiet* OR Panic* OR Worry* OR fear* OR "Psychological distress" OR "Psychological stress" OR "Separation anxiety" OR "Phobic disorder*" OR Agoraphobia OR "Social anxiety" OR "Social phobia" OR "generali*ed anxiety disorder*" OR "Selective mutism") AND title("Longitudinal study" OR "Observational study" OR "Cohort study" OR "Case control study" OR "Time series study" OR "Prospective study" OR "Retrospective study" OR "Follow-up" OR "Cross-sectional study" OR "Risk factor*" OR Correlat OR "Causal factor*" OR Associat OR "Birth cohort" OR "High risk" OR "At risk" OR "Resource factor*" OR Predictor* OR Determinant* OR "Protective factor*" OR "Predictive factor" OR Correlat OR Regression OR "Multivariate analysis" OR Etiolog*)

## Scopus

Abstract search results: 315

( ABS ( adolescen* OR "Young adult*" OR youth OR "Emerging adult*" OR teen* OR "young people" OR "high school student*" OR "high school student*" ) AND ABS ( "Sex difference*" OR "Gender difference*" OR "Gender-sensitiv*" OR "Gender-specific*" OR "Sex-specific*" OR "Gender-related" OR "Gender sensitiv*" OR "Gender specific*" OR "Sex specific*" OR "Gender related" OR genderrelated OR gender* ) AND ABS ( anxiet* OR panic* OR worry* OR fear* OR "Psychological distress" OR "Psychological stress" OR "Separation anxiety" OR "Phobic disorder*" OR agoraphobia OR "Social anxiety" OR "Social phobia" OR "generali*ed anxiety disorder*" OR "Selective mutism" ) AND ABS ( "Longitudinal study" OR "Observational study" OR "Cohort study" OR "Case control study" OR "Time series study" OR "Prospective study" OR "Retrospective study" OR "Follow-up" OR "Cross-sectional study" OR "Risk factor*" OR correlat$ OR "Causal factor*" OR associat$ OR "Birth cohort" OR "High risk" OR "At risk" OR "Resource factor*" OR predictor* OR determinant* OR "Protective factor*" OR "Predictive factor" OR correlat$ OR regression$ OR "Multivariate analysis" OR etiolog* ) ) AND PUBYEAR > 2023 AND ( LIMIT-TO ( SUBJAREA , "PSYC" ) OR LIMIT-TO ( SUBJAREA , "SOCI" ) )

Title search results: 7

( TITLE ( adolescen* OR "Young adult*" OR youth OR "Emerging adult*" OR teen* OR "young people" OR “high school student*" OR "high school student*") AND TITLE ( "Sex difference*" OR "Gender difference*" OR "Gender-sensitiv*" OR "Gender-specific*" OR "Sex-specific*" OR "Gender-related" OR "Gender sensitiv*" OR "Gender specific*" OR "Sex specific*" OR "Gender related" OR genderrelated OR gender* ) AND TITLE ( anxiet* OR panic* OR worry* OR fear* OR "Psychological distress" OR "Psychological stress" OR "Separation anxiety" OR "Phobic disorder*" OR agoraphobia OR "Social anxiety" OR "Social phobia" OR "generali*ed anxiety disorder*" OR "Selective mutism" ) AND TITLE ( "Longitudinal study" OR "Observational study" OR "Cohort study" OR "Case control study" OR "Time series study" OR "Prospective study" OR "Retrospective study" OR "Follow-up" OR "Cross-sectional study" OR "Risk factor*" OR correlat$ OR "Causal factor*" OR associat$ OR "Birth cohort" OR "High risk" OR "At risk" OR "Resource factor*" OR predictor* OR determinant* OR "Protective factor*" OR "Predictive factor" OR correlat$ OR regression$ OR "Multivariate analysis" OR etiolog* ) ) AND PUBYEAR > 2023 AND ( LIMIT-TO ( SUBJAREA , "PSYC" ) OR LIMIT-TO ( SUBJAREA , "SOCI" ) )
