# Supplementary material for: Exploring the role of modifiable sex/gender-specific risk and protective factors for anxiety among young people in high income countries: a systematic narrative review
Source: BMC Public Health. 2026 Feb 18;26:1292. doi: 10.1186/s12889-026-26447-9 (PMC13097721; doi:10.1186/s12889-026-26447-9)
Supplement: Supplementary file 3 — Additional file 3. Appendix 1. Included studies characteristics. Appendix 2. National Institutes of Health Quality Assessment Tool. Appendix 3. Quality assessment of included studies. Appendix 4. Certainty of evidence GRADE assessment. Appendix 5. Individual-level sex/gender results. Appendix 6. Interpersonal-level sex/gender results. Appendix 7. Local community-level sex/gender results. Appendix 8. Wider society-level sex/gender results. [file 12889_2026_26447_MOESM3_ESM.docx]

**Additional file 3: Appendices**

Table of Contents

[Appendix 1. Characteristics, aims and key findings of the studies included in the systematic review 1](#_Toc219283709)

[Appendix 2. National Institutes of Health Quality Assessment Tool for Observational Cohort and Cross-Sectional Studies 36](#_Toc219283710)

[Appendix 3. Quality assessment of included studies 37](#_Toc219283711)

[Appendix 4. Certainty of evidence GRADE assessment 41](#_Toc219283712)

[Appendix 5. Individual-level: Summary of results for sex/gender-specific modifiable factors 42](#_Toc219283713)

[Appendix 6. Interpersonal-level: Summary of results for sex/gender-specific modifiable factors 49](#_Toc219283714)

[Appendix 7. Local community-level: Summary of results for sex/gender-specific modifiable factors 52](#_Toc219283715)

[Appendix 8. Wider society-level: Summary of results for sex/gender-specific modifiable factors 53](#_Toc219283716)

# **Appendix 1. Characteristics, aims and key findings of the studies included in the systematic review**

| **Authors, year, country** | **Population**  (mean age (range), sex/gender (% female/women/girls), demographics,  Setting) | **Study design** | **Sample size** | **Aim** | **Exposure** | **Anxiety type** | **Anxiety measure** | | **Effect size** | | | **Key findings** |
| --- | --- | --- | --- | --- | --- | --- | --- | --- | --- | --- | --- | --- |
|  |  |  |  |  |  |  |  |  | **Sex/gender difference** | **Males/men/boys** | **Females/women/girls** |  |
| Altin et al. (2024) (1) | 23 (NR), 60,  University | Cross-sectional | 332 | To explore the precise link between PPU and suicidality, but also PPU’s association with loneliness, emotional states, and life satisfaction | PPU | Anxiety | Depression, Anxiety, and Stress Scale (DASS-21 | | No formal test for sex/gender difference | **Correlation coefficient = 0.19, p<0.05** | **Correlation coefficient = 0.3, p<0.001** | Positive associations between anxiety and cyber-pornography addiction and loneliness in males and females. |
|  |  |  |  |  | Loneliness |  |  |  |  | **Correlation coefficient = 0.37, p < 0.001** | **Correlation coefficient = 0.42, p<0.001** |  |
| Apsley et al. 2020 (2) USA | 18.32 (NR), 51.8, 60% families European American, 11% African American, 18% had a combination of two or more ethnicities. | Longitudinal  Baseline: 2007  Time 2:2009 Time 3:2010 Time 4:2014 | 500 families (338 fathers and 500 mothers) | To identify variables in the family system that predicted adolescents’ anxious symptoms. Adolescent sex was explored as a moderator of these relations. | Time 3 Maternal connection | Time 4 Anxiety symptoms | Centre for Epidemiological Studies Depression Scale for Children | | No formal test for sex/gender difference | **Standardised estimate = -0.21, p=0.001** | Standardised estimate = 0.11, p = 0.079 | Maternal psychological control was a risk factor of anxiety in females but not in males, whereas maternal connection was a protective factor for males (p=0.001) but not females. Authors attributed findings to theories of females being more emotionally reactive to interpersonal stress than males. |
|  |  |  |  |  | Time 3 Mother's psychological control |  |  |  |  | Standardised estimate = -0.05, p>0.05 | **Standardised estimate = 0.18, p = 0.005** |  |
| Araia et al. 2020 (3) Australia | 16 (10-19), 62, Type 1 diabetics | Cross-sectional | 477 | To explore potential risk and protective psychological correlates for disordered eating in youth with type 1 diabetes by considering a multitude of general and diabetes-specific risk factors, including diabetes distress and diabetes- related resilience. | Diabetes distress | Anxiety symptoms | Generalized Anxiety Disorder scale (GAD-7) | | No formal test for sex/gender difference | **Correlation coefficient = 0.54, p<0.0001** | **Correlation coefficient = 0.52, p<0.0001** | Diabetes distress was a risk factor for anxiety in both males and females and diabetes-related resilience was a protective factor in both males and females.  There was evidence of a positive associations between body dissatisfaction and anxiety in both males and females. |
|  |  |  |  |  | Diabetes resilience |  |  |  |  | **Correlation coefficient = -0.44, p<0.0001** | **Correlation coefficient = -0.33, p<0.0001** |  |
|  |  |  |  |  | Body Mass Index Silhouette Matching Test (BMI-SMT) Actual |  |  |  |  | Correlation coefficient = 0.14 | **Correlation coefficient = 0.27, p<0.0001** |  |
|  |  |  |  |  | BMI-SMT Ideal |  |  |  |  | Correlation coefficient = 0.12 | **Correlation coefficient = -0.15, p<0.05** |  |
|  |  |  |  |  | BMI-SMT without Diabetes |  |  |  |  | **Correlation coefficient = 0.23, p<0.001** | Correlation coefficient = 0.01 |  |
|  |  |  |  |  | BMI-SMT Actual – Ideal Difference |  |  |  |  | Correlation coefficient = 0.08 | **Correlation coefficient = 0.34, p<0.0001** |  |
|  |  |  |  |  | BMI-SMT Actual – without Diabetes Difference |  |  |  |  | Correlation coefficient = -0.10 | **Correlation coefficient = 0.24, p<0.0001** |  |
|  |  |  |  |  | BMI-SMT without Diabetes – Ideal Difference |  |  |  |  | **Correlation coefficient = 0.18, p<0.05** | **Correlation coefficient = 0.13, p<0.05** |  |
| Arsandaux et al. 2023 (4) France | 20.7 (18-30), 78.8, University | Cross-sectional | 6602 | To investigate self-esteem associated factors related to childhood/adolescence or young-adulthood in male and female college students. | Self-esteem | Anxiety symptoms | State-Trait Anxiety Inventory (STAI-S) | | No formal test for sex/gender difference | **Correlation coefficient = -0.76, p<0.0001** | **Correlation coefficient = -0.74, p<0.0001** | Self-esteem was a protective factor for anxiety in both males and females |
| Atkinson et al. 2019 (5) USA | 21.3 (18-30), 46, Community, 78% Caucasian, 14% African American, 5% Asian, 2% Hispanic, and 1% Pacific Islander | Cross-sectional | 810 | To examine sex differences in the association between alcohol problems and anxiety. | Alcohol problems | State-Trait Anxiety | Spielberger State-Trait Anxiety Inventory (STAI) | | **z = 2.04, p<0.05** | **Correlation coefficient = 0.379, p<0.0001** | **Correlation coefficient = 0.249, p<0.0001** | Lifetime alcohol problems had a stronger association with anxiety in males than in females. This sex difference was statistically significant. |
| Barcaccia et al. 2018 (6) Italy | 17 (14-19), 49.17, High School | Cross-sectional | 310 | To evaluate the influence of models offered by the mass media on adolescents’ perceptions of their own body image and on psychopathology. | Hours media watched | Anxiety symptoms | State-Trait Anxiety Inventory (STAI-S) | | No formal test for sex/gender difference | Standardised regression coefficient = 0.072 | Standardised regression coefficient = -0.046 | There was no association between anxiety and a desire to be similar to favourite media characters or the discrepancy perceived between their body image and that of the character in males or females.  Friends’ desire to physically resemble TV characters was associated with anxiety in both males and females |
|  |  |  |  |  | Idolization of characters |  |  |  |  | Standardised regression coefficient = 0.079 | Standardised regression coefficient = 0.032 |  |
|  |  |  |  |  | Desire to be similar |  |  |  |  | Standardised regression coefficient = 0.01 | Standardised regression coefficient = 0.121 |  |
|  |  |  |  |  | Discrepancy from ideal |  |  |  |  | **Standardised regression coefficient = 0.183/2.184, p<0.05** | **Standardised regression coefficient = 0.286/2.329, p<0.01** |  |
| Barry et al. 2015 (7) USA | 16.78 (16-18), 0% females.  Males attending a residential program having dropped out of school. 62.5% Caucasian, 29.1% African-American, and 3.2% other ethnicities | Cross-sectional | 251 | To consider the connection between self-compassion, narcissism, self-esteem, aggression, and internalizing problems. | Self-compassion | Anxiety symptoms | Personality Inventory for Youth (PIY) | | NA | **Correlation coefficient = -0.32, p<0.001** | NA | In this male-only study, self-esteem and self-compassion were risk factors for anxiety |
|  |  |  |  |  | Self-esteem |  |  |  |  | **Correlation coefficient = -0.43, p<0.001** |  |  |
| Baurmeister et al. 2017 (8) USA | Cisgender sexual minority males,  20.8 (18.24), 0% females.  Community | Cross-sectional study | 1484 | To examine the relationship between parental gender policing during childhood and adolescence and subsequent substance use and psychological distress. | Gender policing model | | | | | | | A statistically significant positive association was found between anxiety symptoms and participants being told to stop acting feminine when they were growing up, as well as disciplinary actions related to gender policing. |
|  |  |  |  |  | Gender policing | Anxiety symptoms | | Brief Symptom Inventory | NA | **Unstandardised regression coefficient = 0.40, Standardised regression coefficient = = 0.19, p<0.001** | NA |  |
|  |  |  |  |  | Age |  |  |  |  | **Unstandardised Regression coefficient = 0.04, Standardised regression coefficient = 0.08, p<0.05** |  |  |
|  |  |  |  |  | Completed high school |  |  |  |  | **Unstandardised Regression coefficient = 0.32, Standardised regression coefficient = 0.06, p<0.05** |  |  |
|  |  |  |  |  | Sexual identity - Bisexual |  |  |  |  | Unstandardised Regression coefficient = 0.22  Standardised = 0.04 |  |  |
|  |  |  |  |  | Sexual identity - Other |  |  |  |  | **Unstandardised Regression coefficient = 0.29, Standardised regression coefficient = 0.06, p<0.05** |  |  |
|  |  |  |  |  | Race/ethnicity - Black |  |  |  |  | Unstandardised Regression coefficient = 0.13, Standardised regression coefficient = -0.04 |  |  |
|  |  |  |  |  | Race/ethnicity - Asian/Pacific Islander |  |  |  |  | Unstandardised Regression coefficient = 0.03, Standardised regression coefficient = 0.005 |  |  |
|  |  |  |  |  | Race/ethnicity - Latino |  |  |  |  | **Unstandardised Regression coefficient = 0.17, Standardised regression coefficient = 0.06, p<0.05** |  |  |
|  |  |  |  |  | Race/ethnicity - Multiracial/other |  |  |  |  | Unstandardised Regression coefficient = -0.18, Standardised regression coefficient = -0.04 |  |  |
|  |  |  |  |  | Disciplinary actions model | | | | | | |  |
|  |  |  |  |  | Disciplinary actions | Anxiety symptoms | | Brief Symptom Inventory | NA | **Unstandardised Regression coefficient = 0.15, p<0.005  Standardised Regression coefficient = 0.13** | NA |  |
|  |  |  |  |  | Age |  |  |  |  | **Unstandardised Regression coefficient = -0.07, p<0.005  Standardised = -0.12** |  |  |
|  |  |  |  |  | Completed high school |  |  |  |  | Unstandardised Regression coefficient = 0.26  Standardised = 0.03 |  |  |
|  |  |  |  |  | Sexual identity - Bisexual |  |  |  |  | Unstandardised Regression coefficient = 0.48  Standardised = 0.08 |  |  |
|  |  |  |  |  | Sexual identity - Other |  |  |  |  | **Unstandardised Regression coefficient = 0.56, p<0.05  Standardised = 0.1** |  |  |
|  |  |  |  |  | Race/ethnicity - Black |  |  |  |  | Unstandardised Regression coefficient = -0.04  Standardised = -0.01 |  |  |
|  |  |  |  |  | Race/ethnicity - Asian/Pacific Islander |  |  |  |  | Unstandardised Regression coefficient = 0.17  Standardised = 0.04 |  |  |
|  |  |  |  |  | Race/ethnicity - Latino |  |  |  |  | Unstandardised Regression coefficient = 0.34  Standardised = 0.13 |  |  |
|  |  |  |  |  | Race/ethnicity - Multiracial/other |  |  |  |  | Unstandardised Regression coefficient = 0.03  Standardised = 0.01 |  |  |
| Bauermeister et al. 2010 (9) USA | 17.01 (15-19), 45, 42% Latino/Hispanic, 21% Black/African-American; 24% White; and 13% other race/ethnicity. The sample self-identified as ‘‘mostly gay or lesbian’’ in their Kinsey scale score. GLBT drop-in centres | Longitudinal (1 year follow-up) | 350 | To examine the association between SSR trajectories and psychological well-being over time. | Rate attraction to males/females | Anxiety symptoms | | Brief Symptom Inventory | No formal test for sex/gender difference | Regression coefficient = -0.06 | Regression coefficient = -0.02 | Social support was a protective factor for anxiety in males and females. |
|  |  |  |  |  | Out to family |  |  |  |  | Regression coefficient = -0.03 | Regression coefficient = 0.01 |  |
|  |  |  |  |  | Out to friends |  |  |  |  | Regression coefficient = 0.04 | Regression coefficient = 0.04 |  |
|  |  |  |  |  | T1 Social support |  |  |  |  | Regression coefficient = 0.08 | **Regression** **coefficient = 0.15, p≤0.10** |  |
|  |  |  |  |  | T2 Social support |  |  |  |  | **Regression coefficient = -0.22, p ≤ 0.01** | **Regression coefficient = -0.20, p ≤ 0.01** |  |
|  |  |  |  |  | Serious SSR in past year at baseline and no SSR in past year at follow-up |  |  |  |  | Regression coefficient = -0.23 | Regression coefficient = -0.18 |  |
|  |  |  |  |  | No serious SSR in past year at baseline and one or more SSR in past year at follow-up |  |  |  |  | Regression coefficient = -0.08 | Regression coefficient = 0.03 |  |
|  |  |  |  |  | One or more SSR in at baseline and follow-up |  |  |  |  | Regression coefficient = 0.06 | Regression coefficient = -0.11 |  |
|  |  |  |  |  | T1 Opposite-sex relationship |  |  |  |  | Regression coefficient = -0.09 | Regression coefficient = -0.11 |  |
|  |  |  |  |  | T2 Opposite-sex relationship |  |  |  |  | Regression coefficient = 0.35 | Regression coefficient = 0.13 |  |
|  |  |  |  |  | Age |  |  |  |  | Regression coefficient = -0.01 | Regression coefficient = 0.04 |  |
| Bekman et al 2013 (10) USA | NR (16-18), heavy episodic group (HED): 49, control group (CON): 46. Students with a history of recent heavy episodic drinking and peer controls. Colleges, university and community settings. | Longitudinal (4–6 week follow-up) | 65 | This study examined differences in mood during early abstinence among youth with recent HED compared to peer CON and affective changes over 4–6 weeks of continued abstinence. | Group (HED and CON) x sex | State-Trait Anxiety | | Spielberger State-Trait Anxiety Inventory (STAI) | Regression coefficient: 0.00, p=0.952 | NR | NR | The interaction between group (HED or CON) and anxiety after sustained abstinence were not moderated by sex |
| Benedetto et al. 2018 (11) Italy | 16 (13-18), 48.7,  High school | Cross-sectional | 191 | To assess how parenting factors may contribute to anxiety. | Maternal emotional availability | Anxiety symptoms | | Revised Children's Manifest Anxiety Scale (RCMAS) | No formal test for sex/gender difference | **Correlation coefficient = -0.21, p<0.05** | **Correlation coefficient = -0.35, p<0.01** | Paternal autonomy support was a protective factor for anxiety in males and maternal autonomy support was a protective factor in females.  While paternal and maternal emotional availability were protective factors for anxiety in males, only maternal emotional availability was a protective factor in females. |
|  |  |  |  |  | Paternal emotional availability |  |  |  |  | **Correlation coefficient = -0.30, p<0.01** | Correlation coefficient = -0.06 |  |
|  |  |  |  |  | Maternal psychological control |  |  |  |  | **Correlation coefficient = 0.25, p<0.01** | **Correlation coefficient = 0.45, p<0.01** |  |
|  |  |  |  |  | Paternal psychological control |  |  |  |  | **Correlation coefficient = 0.31, p<0.01** | **Correlation coefficient = 0.19, p<0.05** |  |
|  |  |  |  |  | Maternal autonomy support |  |  |  |  | Correlation coefficient = -0.13 | **Correlation coefficient = -0.33, p<0.01** |  |
|  |  |  |  |  | Paternal autonomy support |  |  |  |  | **Correlation coefficient = -0.31, p<0.01** | Correlation coefficient = -0.04 |  |
| Berenz et al. 2019 (12) USA | 18 (NR), 74.7  Men: White: 66.6%, Black/African American: 15.87%, Asian: 6.35%, Hispanic/Latino: 6.35%, <1 Race: 3.17%, Native Hawaiian: 0%  Women: White: 58.42%, Black/African American: 18.81%, Asian: 7.92%, Hispanic/Latino: 6.93%, <1 Race: 6.93%, Native Hawaiian: 0.50%  University | Cross-sectional | 269 | To evaluate patterns of association between age of initiation of regular alcohol use and posttraumatic stress disorder, anxiety, and depressive symptoms as a function of sex. | Family history of alcohol use disorder (AUD) | Anxiety symptoms | | Symptom Checklist-90 | No formal test for sex/gender difference | Correlation coefficient = 0.1  Step 1: Standardised regression coefficient = 0.01, 95%CI: -0.32, 0.31, p = 0.96  Step 2: Standardised regression coefficient = 0.004, 95%CI: -0.31, 0.31, p = 0.98 | **Correlation coefficient = 0.14, p<0.05**  Step 1: Standardised regression coefficient = 0.07, 95%CI: -0.06, 0.18, p = 0.28  Step 2: Standardised regression coefficient = 0.07, 95%CI: -0.06, 0.20, p = 0.27 | Lifetime traumatic events and family history of anxiety, depression or alcohol use disorder were risk factors for anxiety in females but not in males. |
|  |  |  |  |  | Family history of anxiety or depression |  |  |  |  | Correlation coefficient = 0.18 Step 1: Standardised regression coefficient = 0.18, 95%CI: -0.15, 0.46, p = 0.24  Step 2: Standardised regression coefficient = 0.17, 95%CI: -0.17, 0.43, p = 0.28 | **Correlation coefficient = 0.25, p<0.01**  **Step 1: Standardised regression coefficient = 0.18, 95%CI: 0.08, 0.27, p<0.001**  **Step 2: Standardised regression coefficient = 0.17, 95%CI: 0.08, 0.27, p = 0.001** |  |
|  |  |  |  |  | Number of lifetime traumatic events |  |  |  |  | Correlation coefficient = -0.05 Step 1: Standardised regression coefficient = 0.02, 95%CI: -0.25, 0.28, p = 0.9  Step 2: Standardised regression coefficient = 0.02, 95%CI: -0.25, 0.28, p = 0.9 | **Correlation coefficient = 0.28, p<0.01**  **Step 1: Standardised regression coefficient = 0.21, 95%CI: .07, 0.35, p = 0.002**  Step 2: **Standardised regression coefficient = 0.19, 95%CI: 0.06, 0.32, p = 0.003** |  |
|  |  |  |  |  | Age of alcohol use initiation |  |  |  |  | Correlation coefficient = -0.04  Step 2: Standardised regression coefficient = -0.11, 95%CI: -0.28, 0.07, p = 0.22 | **Correlation coefficient = -0.23, p<0.01**  Step 2: **Standardised regression coefficient = -0.15, 95%CI: -0.29, -0.02, p = 0.03** |  |
| Bernard et al. 2017 (13) USA | At wave 1:  Cohort 1: 19.12 (18–210, 53.5  Cohort 2: 18.07 (18–19), 46.50  African American students at predominantly-White university | Longitudinal (8 month follow-up) | 157 | To examine the moderating roles of racial discrimination and gender on the longitudinal association between imposter phenomenon and mental health outcomes in a sample of African American young adults. | Racial discrimination frequency x imposter phenomenon x gender | Anxiety symptoms | | Symptom Checklist-90 | Standardised Regression coefficient = -0.04 Unstandardised Regression coefficient = -0.02, p=0.77 | NR | NR | This study did not find significant evidence that gender moderated the association between racial discrimination frequency and imposter phenomenon in predicting anxiety symptoms. |
|  |  |  |  |  | Racial discrimination bother x imposter phenomenon x gender |  |  |  | Standardised Regression coefficient = 0.13 Unstandardised Regression coefficient = 0.07, p=0.21 |  |  |  |
| Bernusky et al. 2021 (14) Canada | 19.13 (18-25)  Assigned female at birth: 67.1  Gender identity (woman): 65.2  University | Cross-sectional | 1266 | To examine if anxiety symptoms mediated the association between cannabis use frequency and Psychotic-Like Experiences in Canadian emerging adult undergraduates. | Cannabis use x biological sex | Anxiety symptoms | | Generalized Anxiety Disorder scale (GAD-7) | **Unstandardised coefficient = -0.45, standardised coefficient = -0.05, p>0.05** | NR | NR | The cannabis use by sex interaction term did not predict anxiety symptoms. |
| Bieniak et al. 2022 (15) USA | 16 (NR), 70.6,  52.94% non-Hispanic white  Individuals with paediatric h hEDS | Cross-sectional | 34 | To improve understanding of the relationships between functional disability, perceived social support, and mental health in children with hEDS. | Functional disability | Anxiety symptoms | | Pediatric Reported Outcomes Measurement Information System (PROMIS) | Fisher's Z= 0.23, p=0.82 | Correlation coefficient = 0.57, 95% CI: -0.23, 0.91 | **Correlation coefficient = 0.48 , 95% CI: 0.076, 0.75** | Functional disability was a risk factor and social support was a protective factor for anxiety in females but not in males. No statistically significant gender differences were detected. |
|  |  |  |  |  | Social support |  |  |  | Fisher's Z= 1.58, p=0.11 | Correlation coefficient = -0.08, 95% CI: -0.74, 0.66 | **Correlation coefficient = -0.705, 95% CI: -0.87, -0.39** |  |
| Bilali et al. 2025 (16) Greece | 18.5 (18-19), 81.3, Online | Cross-sectional | 219 | To examine the association between TikTok use and anxiety, depression, and sleepiness in adolescents | TikTok Addiction - Mood modification (capacity to enhance one’s emotional state) | Anxiety | | Patient Health Questionnaire-4 (PHQ-4) | No formal test for sex/gender difference | **Unadjusted coefficient beta = 1.130, 95% CI = 0.418 to 1.842, p = 0.003**  **Adjusted coefficient beta = 0.760, 95% CI = 0.121 to 1.399, p-value = 0.021** | **Unadjusted coefficient beta = 0.365, 95% CI = 0.093 to 0.638 , p = 0.009**  **Adjusted coefficient beta = 0.338, 95% CI = 0.031 to 0.645, p-value = 0.031** | “The impact  of TikTok on anxiety and depression was greater among boys than girls.” |
|  |  |  |  |  | TikTok Addiction - Conflict (when TikTok interferes with daily activities) |  |  |  |  | **Unadjusted coefficient beta = 1.1392, 95% CI = 0.926 to 1.858, p<0.001 Adjusted coefficient beta = 1.236, 95% CI = 0.763 to 1.710, p-value < 0.006** | Unadjusted coefficient beta = 0.019, 95% CI =−0.235 to 0.273, p = 0.88  Adjusted coefficient beta = -0.273, 95% CI = -0.581 to 0.034, p = 0.081 |  |
|  |  |  |  |  | TikTok Addiction - Relapse (when users return to previous usage patterns after a period of abstinence) |  |  |  |  | Unadjusted coefficient beta = 0.243, 95% CI = −0.524 to 1.010, p = 0.525  Adjusted coefficient beta = -0.063, 95% CI = −0.660 to 0.533, p = 0.831 | **Unadjusted coefficient beta = 0.283, 95% CI = 0.031 to 0.534, p = 0.028**  Adjusted coefficient beta =0.304, 95% CI = -0.013 to 0.621, p = 0.060 |  |
| Bluth et al. 2017 (17) USA | NR (11-19), 53  4% Hispanic, 64% White and 21 % Black,  High school, older adolescent subgroup | Cross-sectional | 65 | To examine levels of self-compassion across adolescence in middle school and high school and their association with indicators of emotional well-being, including perceived stress, depressive symptoms, anxiety, life satisfaction, and distress intolerance. | Self-Compassion x age x gender | Anxiety symptoms | | Spielberger state–trait anxiety inventory (STAI) | **Regression coefficient = 2.21, 95% CI: 0.58 to 3.83, p = 0.008** | p=0.013 | NR | There is evidence of an interaction between sex/gender, self-compassion and age, such that for those aged 17-19 years, self-compassion was more protective against anxiety in males compared to females. |
| Brolin et al. (2024) (18) Sweden | Baseline: NR (15-16)  Follow-up: NR (20-21),  56.8, School | Longitudinal | 5537 | To investigate the relationship between exposure to parental problem drinking during adolescence and the presence of depression and anxiety symptoms in young adulthood. | Parent problem drinking at age 15-16 | Anxiety at age 20-21 years | | Patient Health Questionnaire-4 (PHQ-4): Generalised Anxiety Disorder (GAD-2) | Wald test for interaction p=0.296 | NR | NR | There was no evidence of sex moderating the positive association between parent problem drinking at age 15-16 and anxiety at age 20-21 years (interaction: p=0.296). |
| Buckner et al. (2024) (19) USA | 19.5 (18-55), 100% ‘Cis-females’, 65.6% Non‐Hispanic/Latin White, 7.7% Hispanic/Latin White, 14.8% Non‐  Hispanic/Latin African American/Black, 0.5% Hispanic/Latin African  American/Black, 3.4% Non‐Hispanic/Latin Asian/Asian American,  0.1% Hispanic/Latin Asian/Asian American, 0.1% Asian Indian, 0.2%  Non‐Hispanic/Latin American Indian/Alaska Native, 0.2 Hispanic/Latin  American Indian/Alaska Native, 4.5% multiracial, and 2.0% “race not  listed”, University | Cross-sectional | 836 | To further understand the impact of sexism on alcohol‐related outcomes in several ways. “First, we tested our hypothesis that sexism would be related to social  anxiety.” | Sexism | Social anxiety | | Social Interaction Anxiety Scale (SIAS) | NA | NA | **Correlation coefficient = 0.24, p < 0.01** | Past‐year experiences with sexism were positively correlated with  Social anxiety. |
|  |  |  |  |  | Drinking to cope |  |  |  |  |  | **Correlation coefficient = 0.38, p < 0.01** |  |
|  |  |  |  |  | Drinking to conform |  |  |  |  |  | **Correlation coefficient = 0.26, p < 0.01** |  |
|  |  |  |  |  | Drinking to be social |  |  |  |  |  | **Correlation coefficient = 0.21, p < 0.01** |  |
|  |  |  |  |  | Drinking to enhance enjoyment |  |  |  |  |  | **Correlation coefficient = 0.17, p < 0.01** |  |
|  |  |  |  |  | Alcohol-related problems |  |  |  |  |  | **Correlation coefficient = 0.17, p < 0.01** |  |
|  |  |  |  |  | Peak blood alcohol content |  |  |  |  |  | Correlation coefficient = -0.07 |  |
| Burke et al. (2023) (20)  USA | 19.9 (18-25), Black women  Community | Cross-sectional | 163 | To examine the associations between GRMS and GRMF and Black women's social anxiety and general anxiety symptoms. | GRMF | Social anxiety symptoms | | Social Interaction Anxiety Scale (SIAS) | NA | NA | Correlation coefficient = 0.1, p=0.212  **Standardised regression coefficient = -0.44, p=0.002** | GRMS was positively correlated with increased social anxiety, whereas GRMF was not.  Frequency of gendered racial microaggressions and GRMS were each positively correlated with increased general anxiety  GRMF was negatively associated with increased social anxiety, whereas GRMS was positively associated with increased social anxiety  GRMF was not associated with general anxiety whereas GRMS was positively associated with increased general anxiety. |
|  |  |  |  |  | GRMS |  |  |  |  |  | **Correlation coefficient = 0.26, p<0.01**  **Standardised regression coefficient = 0.63, p<0.001** |  |
|  |  |  |  |  | Age |  |  |  |  |  | Correlation coefficient = 0.01 |  |
|  |  |  |  |  | Income |  |  |  |  |  | Correlation coefficient = -0.17 |  |
|  |  |  |  |  | GRMF | Generalised anxiety symptoms | | Depression, Anxiety and Stress Scale-21 (DASS-21) |  |  | **Correlation coefficient = 0.44, p<0.01**  Standardised regression coefficient = 0.07, p=0.567 |  |
|  |  |  |  |  | GRMS |  |  |  |  |  | **Correlation coefficient = 0.49, p<0.01**  **Standardised regression coefficient = 0.43, p<0.01** |  |
|  |  |  |  |  | Age |  |  |  |  |  | Correlation coefficient = -0.02 |  |
|  |  |  |  |  | Income |  |  |  |  |  | Correlation coefficient = -0.12 |  |
| Cano et al. (2021) (21)  USA | 21.3 (18-25), 50  Hispanic young adults Community | Cross-sectional study | 200 | To examine associations of exposure to ethnic discrimination in social media with symptoms of depression and generalized anxiety and the extent to which gender moderates these respective associations. | Social media ethnic discrimination | Generalised anxiety symptoms | | State-Trait Anxiety Inventory (STAI-S) | **Standardised regression coefficient = −0.33, p=0.01** | **Standardised regression coefficient = 0.28, p≤0.001** | Standardised regression coefficient = −0.05, p = 0.58 | Gender had a statistically significant interaction with exposure to social media ethnic discrimination in predicting generalized anxiety symptoms; increasing levels of ethnic discrimination in social media was associated with higher symptoms of generalized anxiety among males but not females.  Authors suggested that this is due to males being more likely to be depicted in, or exposed to, racist or discriminatory content on social media and to respond and engage with the perpetrators, whereas females are more avoidant or seek social support in response. |
| Carcedo et al. (2020) (22) Spain | 19.92 (18-29), 55.9  Secondary school and university | Cross-sectional | 1682 | To study differences in sexual satisfaction and mental health (anxiety and depression) based on romantic relationship status (having a partner vs. not having one) and gender | Sexual satisfaction × Gender | Anxiety symptoms | | Symptom Checklist-90 | Regression coefficient = -0.01, 95% CI: -0.10, 0,07 | NR | NR | No significant effects were observed for the interaction between gender and relationship status.  No interaction between gender and relationship status with respect to anxiety was found. |
|  |  |  |  |  | Relationship status x Gender |  |  |  | “*No significant effects were observed for gender nor were they observed for the interaction between gender and relationship status*” (not reported) | NR | NR |  |
| Carlberg et al. (2024) (23) Sweden | 16.70 (12-22), “overrepresentation of female respondents”, Child and Adolescent Psychiatric Clinic | Cross-sectional | 574 | To measure the associations between psychiatric symptoms and exposure to UOSS, offline sexual harassment, cyberbullying, and offline bullying, and to analyse possible differences between boys and girls. | UOSS | Anxiety | | The revised child anxiety and depression scale (RCADS) | **Interaction: Standardised beta coefficients**  **= 0.33, p = <0.001** | NR | NR | Boys exposed to UOSS had higher levels of symptoms than girls exposed to UOSS. |
|  |  |  |  |  | Offline sexual harassment |  |  |  | Interaction: Standardised beta coefficients  = 0.05, p = 0.291 | NR | NR |  |
|  |  |  |  |  | Cyberbullying |  |  |  | Interaction: Standardised beta coefficients = −0.06, p = 0.200 | NR | NR |  |
|  |  |  |  |  | Offline bullying |  |  |  | Interaction: Standardised beta coefficients = −0.03, p = 0.546 | NR | NR |  |
| Carollo et al. (2024) (24) Italy | 20.84 (18-29), 75.41, University | Cross-sectional | 370 | To examine i) the relationship between demographic variables and key features  of anxiety disorders (i.e., worry and anxiety symptoms); and ii) the predictive association between early parental  bonding and anxiety-related features. | Parental care | Worry | | Penn State Worry Questionnaire (PSWQ) |  | **Standardised regression coefficient** **= −3.642, p=0.002** | Standardised regression coefficient = -0.920, p=0.153 | “The predictive relationship between parental care and parental  overprotection exhibited a gender-specific effect: lower care was a more  stable predictor of anxiety features in males, while higher over-  protection was more significant for female participants.” |
|  |  |  |  |  | Parental overprotection |  |  |  |  |  | Standardised regression coefficient **= 1.677, p=0.008** |  |
|  |  |  |  |  | Parental care | Anxiety | | Beck Anxiety Inventory (BAI) |  | Standardised regression coefficient **= -3.462, p < 0.001** | **Standardised regression coefficient = -1.249, p=0.044** |  |
|  |  |  |  |  | Parental overprotection |  |  |  |  |  | **Standardised regression coefficient = 2.204, p < 0.001** |  |
| Chen et al. (2021) (25)  Taiwan | 24.63 (NR), 50,  Lesbian, gay, and bisexual (LGB) young adults | Cross-sectional | 1000 | To examine the association between sexual orientation microaggression and anxiety and depression among young adult lesbian, gay, and bisexual individuals in Taiwan, as well as and the moderating effect of gender. | Age (years) | Anxiety symptoms | | State-Trait Anxiety Inventory (STAI-S) | NR | Correlation coefficient = 0.02, p = 0.62 | Correlation coefficient = 0.03, p = 0.57 | Sexual orientation microaggression and self-identity disturbance were risk factors for anxiety in males and females. No evidence of gender difference were found. |
|  |  |  |  |  | Age of identifying sexual orientation (years) |  |  |  | NR | Correlation coefficient = 0.01, p = 0.89 | Correlation coefficient = 0.77, p = 0.09 |  |
|  |  |  |  |  | Sexual orientation microaggression |  |  |  | χ2 = 0.48, p = 0.49 | **Correlation coefficient = 0.19, p<0.001**  **Standardised regression coefficient = 0.096, p<0.05** | **Correlation coefficient = 0.35, p<0.001 Standardised regression coefficient = 0.235, p<0.001** |  |
|  |  |  |  |  | Self-identity disturbance |  |  |  | χ2 = 0.29, p=0.59 | **Correlation coefficient = 0.55, p<0.001**  **Standardised regression coefficient = 0.597, p<0.001** | **Correlation coefficient = 0.55, p<0.001 Standardised regression coefficient = 0.535, p<0.001** |  |
| Christiansen et al. 2021 (26) Denmark | 22.6 (NR), 51.6  Population-based: Danish registration System | Cross-sectional | 19,890 | To investigate whether loneliness and social isolation are associated with poor physical and mental health among adolescents and young adults, and whether age and gender play a role in the associations of loneliness and social isolation with mental and physical health. | Loneliness × Gender | Anxiety symptoms | | Questionnaire | Adjusted OR = 0.96, 95%CI: 0.88–1.04 | NR | NR | Gender did not moderate the associations between anxiety and loneliness and social isolation. |
|  |  |  |  |  | Social isolation× Gender |  |  |  | Adjusted OR = 1.06, 95%CI: 0.93–1.22 | NR | NR |  |
| Davila et al. 2020 (27) USA | 16.33 (12-18), 28  Non-Hispanic White: 25.4% Hispanic: 44.9% African-American: 20.6%  Other: 9.1%  Adolescents receiving substance abuse treatment services | Cross-sectional (from longitudinal study) | 394 | To examine the co-patterning of childhood maltreatment experiences and serious internalizing problems (for past- year anxiety and depression symptoms and current distress) and gender differences in these relations. | Sexual abuse | Anxiety symptoms | | Composite International Diagnostic Interview (CIDI) | No formal test for sex/gender difference | Correlation coefficient = 0.12 | Correlation coefficient = -0.03 | No evidence was found of statistically significant associations between sexual abuse and punishment and anxiety in males or females.  Neglect was a risk factor for anxiety in males but not in females, however no formal test for gender difference was conducted.  Alcohol and drug abuse or dependence symptoms were risk factors of anxiety in both males and females. |
|  |  |  |  |  | Punishment |  |  |  |  | Correlation coefficient = 0.11 | Correlation coefficient = -0.01 |  |
|  |  |  |  |  | Neglect |  |  |  |  | **Correlation coefficient = 0.20, p<0.01** | Correlation coefficient = 0.12 |  |
|  |  |  |  |  | Alcohol abuse or dependence symptoms |  |  |  |  | **Correlation coefficient = 0.15, p<0.05** | **Correlation coefficient = 0.23, p<0.05** |  |
|  |  |  |  |  | Drug abuse or dependence symptoms |  |  |  |  | **Correlation coefficient = 0.26, p<0.01** | **Correlation coefficient = 0.24, p<0.01** |  |
| Davis et al. 2022 (28) USA | 18.3 (17-24), 54.3  Hispanic: 45.4%  Non-Hispanic white: 20.5%  Non-Hispanic Black: 2.24%  Asian: 20.2%  Multiracial/Other: 11.4%  High school and University | Longitudinal (annual follow-up) | 2,995 | To better understand directionality between anxiety symptomology and cannabis use among young adults and how this may vary between men and women. | Cannabis use (Proportional coupling parameters) | Anxiety symptoms | | Generalized Anxiety Disorder scale (GAD-7) | No formal test for sex/gender difference | **Unstandardised regression coefficient = 0.06, p < 0.05** | **Unstandardised regression coefficient = 0.06, p < 0.05** | Cannabis use was positively associated with anxiety in both men and women.  For men, in support of a substance-induced pathway, results revealed prior levels of cannabis use (i.e., higher use) are associated with greater subsequent increases in anxiety.  For women, results support both symptom-driven and substance-induced models such that prior levels of cannabis use are associated with greater subsequent increases in anxiety, and also prior anxiety is associated with increases in cannabis use. |
|  |  |  |  |  | Cannabis use (Dynamic coupling parameters) |  |  |  |  | Unstandardised regression coefficient = 0.31 | **Unstandardised regression coefficient = -0.11, p < 0.05** |  |
| De France et al. 2022 (29) | Follow-up: 16.21 (NR), 53.7 | Longitudinal (2 year follow-up) | Baseline: 184  Follow-up: 136 | To identify whether individual differences in the extent to which participants showed deviations from their personal symptom trajectories could be explained by specific elements of the pandemic. To examine whether our patterns of results were consistent between male and female participants. | Health impacts | Anxiety symptoms | | Multidimensional Anxiety Scale for Children (MASC) | No significant gender difference | Standardised path loadings = 0.06 | Standardised path loadings = 0.09, p = 0.001 | The association between Fear of the Coronavirus and anxiety was significantly higher for males than females. |
|  |  |  |  |  | Financial impacts |  |  |  |  | Standardised path loadings = 0.05 | Standardised path loadings = 0.01 |  |
|  |  |  |  |  | Lifestyle impacts |  |  |  |  | Standardised path loadings = 0.01 | Standardised path loadings = 0.12 |  |
|  |  |  |  |  | Coronavirus fear |  |  |  | Test for sex/ gender difference p=0.001 | Standardised path loadings = 0.22, p = 0.001 | Standardised path loadings = -0.12 |  |
| Di Blasi et al. (30) 2015 | 16.39 (14-19), 51.4  Caucasian: 100%  High school | Cross-sectional | 1305 | To examine potential gender differences in associations between self-image dimensions and social anxiety. | Impulse Control | Social anxiety symptoms | | Social Interaction Anxiety Scale (SIAS) | No formal test for sex/gender difference | OR = 1.027, p=0.350, 95%CI: 0.97-1.09 | OR = 0.980, p=0.418, 95%CI: 0.93-1.03 | The Social Attitude scale and Psychological Health scale were positively associated with social anxiety in both males and females.  The Family Relationships subscale was positively associated with social anxiety in females but not in males.  The External Mastery (a state of adjustment between the subject and his or her environment that is favourable to emotional growth) subscale was positively associated with social anxiety in females but not in males.  The Emotional Tone subscales was negatively associated with social anxiety in males but not in females  Because the confidence intervals overlap, and no formal test for a gender difference was conducted, it cannot be concluded whether gender differences are present. |
|  |  |  |  |  | Emotional Tone |  |  |  |  | **OR = 0.883, p=0.001, 95%CI: 0.82-0.95** | OR = 0.999, p=0.968, 95%CI: 0.94-1.06 |  |
|  |  |  |  |  | Body and Self-Image |  |  |  |  | OR = 0.986, p=0.645, 95%CI: 0.93-1.05 | OR = 0.983, p=0.515, 95%CI: 0.93-1.03 |  |
|  |  |  |  |  | Social Attitudes |  |  |  |  | **OR = 0.923, p=0.007, 95%CI: 0.87-0.98** | **OR = 0.823, p=0.000, 95%CI: 0.78-0.87** |  |
|  |  |  |  |  | Vocational and Educational Goals |  |  |  |  | OR = 1.036, p=0.194, 95%CI: 0.98-1.09 | OR = 1.047, p=0.123, 95%CI: 0.99-1.11 |  |
|  |  |  |  |  | Sexual Attitudes |  |  |  |  | OR = 0.980, p=0.451, 95%CI: 0.93-1.03 | OR = 0.973, p=0.280, 95%CI: 0.92-1.02 |  |
|  |  |  |  |  | Family Relationships |  |  |  |  | OR = 1.057, p=0.065, 95%CI: 0.99-1.12 | **OR = 1.070, p=0.007, 95%CI: 1.02-1.12** |  |
|  |  |  |  |  | External Mastery |  |  |  |  | OR = 0.985, p=0.649, 95%CI: 0.92-1.05 | **OR = 0.92, p=0.003, 95%CI: 0.87-0.97** |  |
|  |  |  |  |  | Psychological Health |  |  |  |  | **OR = 0.907, p=0.007, 95%CI: 0.84-0.97** | **OR = 0.89, p=0.000, 95%CI: 0.84-0.95** |  |
|  |  |  |  |  | Superior Adjustment |  |  |  |  | OR = 0.980, p=0.492, 95%CI: 0.92-1.04 | OR = 0.996, p=0.879, 95%CI: 0.84-0.95 |  |
| Duncan et al. (2019) (31) Australia | 17.9 (12.7-24.3), High school: 52 University: 64  High school and university students | Cross-sectional | 769 | To isolate the effects of sexual harassment on emotional maladjustment.  To consider whether there is a gender difference in this relationship. | Global self-worth | Social anxiety symptoms | | Social Anxiety Scale for Adolescents (SAS-A) | NR | **Correlation coefficient = -0.55, p<0.001** | **Correlation coefficient = -0.57, p<0.001** | There were positive associations between victimisation and sexual harassment and social anxiety in males and females.  There was a negative association between self-worth and anxiety in males and females.  The interaction between sexual harassment, gender and age in predicting social anxiety was significant; there was a positive association between sexual harassment and social anxiety in younger (age 18 and under) females and older (age 18 or older) males, but not in older females and younger males.  This contradicted the authors’ hypothesis that older females would be most affected by sexual harassment, and was attributed to older females having high levels of anxiety regardless of sexual harassment experience. |
|  |  |  |  |  | Sexual harassment |  |  |  | NR | **Correlation coefficient = 0.20, p<0.001** | **Correlation coefficient = 0.12, p<0.01** |  |
|  |  |  |  |  | Appearance-related victimization (general and online) |  |  |  | NR | **Correlation coefficient = 0.40, p<0.001** | **Correlation coefficient = 0.27, p<0.001** |  |
|  |  |  |  |  | Social media victimization |  |  |  | NR | **Correlation coefficient = 0.24, p<0.001** | **Correlation coefficient = 0.26, p<0.001** |  |
|  |  |  |  |  | Age (years) |  |  |  | NR | Correlation coefficient = 0.03 | **Correlation coefficient = 0.09, p<0.05** |  |
|  |  |  |  |  | Sexual harassment × gender × age |  |  |  | **Regression coefficient = −0.36, p = 0.006** | NR | NR |  |
| Durham et al. (2025) (32) USA | 19.27 (12-27), 70.95,  Hispanic or Latino: 11.10%  Non-Hispanic or Latino: 85.30%, Community | Cross-sectional study | 1384 | To examine the relationship between  multiple aspects of emotion regulation  and trait anxiety  in a sample spanning adolescence through young adulthood. | Difficulty with Goal-Directed Behaviour x Negative Life Events x Sex | Trait Anxiety | | State-Trait Anxiety Inventory (STAI) | **Standardised regression coefficient=-0.05, p=0.025** | NR | NR | For males, there was a positive association between negative life events and trait anxiety for the upper and middle terciles (those who have more difficulty engaging in goal-directed behaviour), but a slightly negative relationship for the lower tercile (those with less difficulty engaging in goal-directed behaviour). For females there was a positive association between negative life events and trait anxiety regardless of difficulty engaging in goal-directed behaviour. |
| El-Sheikh et al. (2022) (33)  USA | 17.27 (NR), 49,  White/European American: 59%, Black/African American: 41%  Schools | Cross-sectional | 272 | To examine multiple actigraphy-based sleep parameters as moderators of associations between experiences of general and racial discrimination and adolescent internalizing symptoms (anxiety, depression). Adolescent sex and race were examined as additional moderators. | General discrimination x sleep minutes x sex | Anxiety symptoms | | Revised Children's Manifest Anxiety Scale (RCMAS) | **Unstandardised regression coefficient = -0.74, Standardised regression coefficient = -0.27, p<0.01** | Positive association between general discrimination and anxiety symptoms among males with greater sleep minutes (p<0.001) | Positive association between general discrimination and anxiety symptoms among females with fewer sleep minutes (p<0.001) | Sex/gender moderation occurred such that sleeping habits exacerbated the relationship between discrimination and anxiety in females particularly. |
|  |  |  |  |  | General discrimination x sleep efficiency x sex |  |  |  | Unstandardised regression coefficient = 0.07, Standardised regression coefficient = 0.15 | NR | NR |  |
|  |  |  |  |  | General discrimination x sleep variability in minutes x sex |  |  |  | Unstandardised regression coefficient = -0.02, Standardised regression coefficient = -0.05 | NR | NR |  |
|  |  |  |  |  | Racial discrimination x sleep minutes x sex |  |  |  | **Unstandardised regression coefficient = 0.26, Standardised regression coefficient = 0.19, p<0.05** | Positive association between racial discrimination  and anxiety among males with longer sleep (p<0.01) | Positive association between racial discrimination  and anxiety among females with shorter sleep (p<0.001) |  |
|  |  |  |  |  | Racial discrimination x sleep efficiency x sex |  |  |  | **Unstandardised regression coefficient = 0.04, Standardised regression coefficient = 0.18, p<0.01** | Positive association between racial discrimination  and anxiety among males with lower (p<0.05) and higher sleep efficiency (p<0.001). | Positive association between racial discrimination  and anxiety among females with lower sleep efficiency (p<0.001) |  |
|  |  |  |  |  | Racial discrimination x sleep variability in minutes x sex |  |  |  | **Unstandardised regression coefficient = -0.01, Standardised regression coefficient = -0.08, p<0.05** | Positive association between racial discrimination  and anxiety among  males with less variability in sleep minutes (p<0.001) | Positive association between racial discrimination  and anxiety among females with more (p<0.05) and less variability in sleep minutes (p<0.001) |  |
| Ferro et al. 2012 (34) Canada | 23.5 (15-30), 48 | Cross-sectional | 5947 | To examine the sex-specific prevalence of lifetime mental disorder in an epidemiological sample of emerging adults aged 15–30 years with and without chronic health conditions; quantify the association between chronic health conditions and mental disorder, adjusting for sociodemographic and health factors; and, examine potential moderating and mediating effects of sex, level of disability and pain. | Respiratory | Generalised Anxiety disorder | | World Health Organization (WHO) version of the Composite International Diagnostic Interview 3.0 (WHO-CIDI) | OR=0.85, 95%CI 0.24-2.93 | NR | NR | There was no evidence of sex moderating the positive associations between chronic health conditions and anxiety. |
|  |  |  |  |  | Musculoskeletal/connective tissue |  |  |  | OR=0.72, 95%CI 0.28-1.87 | NR | NR |  |
|  |  |  |  |  | Cardiovascular |  |  |  | OR=1.57, 95%CI 0.19-12.95 | NR | NR |  |
|  |  |  |  |  | Neurological |  |  |  | OR=1.78, 95%CI 0.60-5.29 | NR | NR |  |
|  |  |  |  |  | Endocrine/digestive |  |  |  | OR=0.76, 95%CI 0.14-4.26 | NR | NR |  |
| Fitzsimmons et al. 2012 (35) USA | African American/Black women: 19.04 (18-28)  Caucasian women: 18.58 (18-27)  Baseline: African American/Black: 35%  Caucasian non-Hispanic/White: 65%  Follow-up:  African American: 31%  Caucasian: 69% | Longitudinal (5 month follow-up) | Baseline: 276  Follow-up: 226 | To extend existing empirical research on body surveillance, the indicator of self-objectification, and its relation to body dissatisfaction by using a longitudinal design and a racially/ethnically diverse sample and by examining the role of trait anxiety in this relation. | Baseline: BMI | Baseline: Anxiety symptoms | | Spielberger State-Trait Anxiety Inventory (STAI) | NA | NA | **African American women: Correlation coefficient = 0.26, p<0.05**  Caucasian women: Correlation coefficient = 0.08 | There was evidence of positive associations between anxiety and body surveillance and body dissatisfaction in both Caucasian and African American young women.  No association was found between BMI and anxiety, except in African American young women at baseline only. |
|  |  |  |  |  | Baseline: Body Surveillance |  |  |  | NA | NA | **African American women: Correlation coefficient = 0.60, p<0.001**  **Caucasian women:**  **Correlation coefficient = 0.49, p<0.001** |  |
|  |  |  |  |  | Baseline: Body Dissatisfaction |  |  |  | NA | NA | **African American women: Correlation coefficient = 0.58, p<0.001**  **Caucasian women:**  **Correlation coefficient = 0.55, p<0.001** |  |
|  |  |  |  |  | Follow-up:  BMI | Follow-up:  Anxiety symptoms | |  | NA | NA | African American women: Correlation coefficient = 0.22  Caucasian women: Correlation coefficient = 0.11 |  |
|  |  |  |  |  | Follow-up:  Body Surveillance |  |  |  | NA | NA | **African American women: Correlation coefficient = 0.58, p<0.001**  **Caucasian women: Correlation coefficient = 0.51, p<0.001** |  |
|  |  |  |  |  | Follow-up:  Body Dissatisfaction |  |  |  | NA | NA | **African American women: Correlation coefficient = 0.54, p<0.001**  **Caucasian women: Correlation coefficient = 0.58, p<0.001** |  |
| Fontaine et al. 2019 (36) Canada | 17 (NR), 52.1, “Caucasian majority”  Quebec Birth Registry | Longitudinal (2 year follow-up) | 1515 | To examine the cross-lagged associations between delinquency (nonviolent and violent), depression and anxiety symptoms in a sample of adolescents from a population-based sample and the moderating effect of sex and family SES. | Family SES at 10–12 years | Anxiety symptoms | | Mental Health and Social Inadaptation Assessment | No formal test for sex/gender difference | Correlation coefficient = 0.01 | Correlation coefficient = -0.02 | Nonviolent and violent delinquency at age 15 years was positively associated with anxiety symptoms at age 17 years in males and females, respectively, with no statistically significant sex difference.  Nonviolent and violent delinquency at age 17 years were also positively associated with anxiety symptoms at age 17 years in males and females with no statistically significant sex difference. |
|  |  |  |  |  | Nonviolent delinquency at 15 years |  |  |  | Δχ2=32.22, p =0.65 | **Correlation coefficient = 0.18, p ≤ .001** | **Correlation coefficient = 0.09, p ≤ .05** |  |
|  |  |  |  |  | Nonviolent delinquency at 17 years |  |  |  |  | **Correlation coefficient = 0.18, p ≤ .001** | **Correlation coefficient = 0.19, p ≤ .001** |  |
|  |  |  |  |  | Violent delinquency at 15 years |  |  |  | Δχ2=37.32, p =0.41 | **Correlation coefficient = 0.17, p ≤ .001** | **Correlation coefficient = 0.11, p ≤ .01** |  |
|  |  |  |  |  | Violent delinquency at 17 years |  |  |  |  | **Correlation coefficient = 0.24, p ≤ .001** | **Correlation coefficient = 0.22, p ≤ .001** |  |
| Fortier et al. (2025) (37) Canada | 17 (NR), 52, 94% White, Community | Cross-sectional | 1252 | To examine (1) the bidirectional associations between physical activity and  depressive and anxious symptoms during adolescence while controlling for screen time and sleep, (2) whether these associations are mediated by screen time and sleep, and (3) sex differences. | Physical activity | Anxious symptoms | | Social Behaviour Questionnaire | NR | NR | NR | Physical activity and screen time did not predict later  anxious symptoms in either boys or girls. |
|  |  |  |  |  | Screen time |  |  |  | Sex difference (p≤0.05) | Standardised regression coefficient=-0.092 | Standardised regression coefficient=0.072 |  |
| Gassó et al. (2020) (38) Spain | 21.4 (18-64), 76.3,  University | Cross-sectional | 1358 | To analyse sexting and psychopathology correlates by sex. | Creating and sending nude or sexual imagery of oneself | Anxiety symptoms | | Brief Symptom Checklist | No formal test for sex/gender difference | OR = 1.24, p = 0.345 | OR = 1.12, p = 0.381 | There was a statistically significant positive association between anxiety and sexting behaviours in the female sample, but not the male sample.  These findings suggest that for women receiving unwanted and unsolicited sexual content might be a distress-generating factor, as it is possible that they perceive this action as a form of indirect sexual harassment  These results could be explained by victimizing behaviours triggering greater psychopathological symptomatology or by women who suffer from psychopathology, anxiety, or depression being more vulnerable to being pressured to sext and to different forms of online sexual victimization. |
|  |  |  |  |  | Receiving sexts |  |  |  |  | OR = 0.97, p = 0.907 | **OR = 1.53, p = 0.001** |  |
|  |  |  |  |  | Being a victim of nonconsensual dissemination |  |  |  |  | OR = 2.36, p = 0.206 | **OR = 2.20, p = 0.038** |  |
|  |  |  |  |  | Being pressured to sext |  |  |  |  | OR = 1.37, p = 0.256 | **OR = 1.55, p = 0.001** |  |
|  |  |  |  |  | Being threatened to sext |  |  |  |  | OR = 1.95, p = 0.580 | **OR = 2.12, p = 0.021** |  |
|  |  |  |  |  | Active-passive sexting overlap |  |  |  |  | OR = 1.05, p = 0.837 | OR = 1.51, p = 0.085 |  |
|  |  |  |  |  | Any sexting |  |  |  |  | OR = 1.55, p = 0.075 | **OR = 1.45, p = 0.010** |  |
| Giannotta et al. (2024) (39) Sweden | Time 1: 13 Time 2: 16 Time 3: 19 Time 4: 22  56% Female, Community | Longitudinal  (Follow-up: 3, 6, 9 years) | Time 1: 902 Time 2: 774 Time 3: 608 Time 4: 522 | To examine the bidirectional associations among anxiety symptoms, sleep problems, and physical activity, while also exploring gender differences. | Physical activity | Anxiety symptoms | | Generalised Anxiety Disorder subscale of Spence Children’s Anxiety Scale (SCAS) | NR | NR | NR | “No difference in the model between girls and boys were found.” |
|  |  |  |  |  | Sleep problems |  |  |  | NR | NR | NR |  |
| Gillespie et al. (2023) (40)  North America | Resettled Somali young adults,  21.01 (18-30), 45.4% females  at Time 1 | Longitudinal  Time 1 (May 2013–January 2014), Time 2 (January 2014–August 2015), and Time 3 (December 2016–February 2018) | 185 | To examine whether two acculturation styles (operationalized as cultural assimilation and cultural integration) longitudinally mediate the relation. To examine whether the strength of the indirect effect of discrimination on anxiety through assimilation was moderated by gender. | Age | Anxiety symptoms (Time 3) | | Hopkins Symptom Checklist 25 (HSCL-25) | No formal test for sex/gender difference | Correlation coefficient = -0.014 | Correlation coefficient = -0.217, p<0.1 | A significant positive association was found between assimilation in anxiety in males, not females.  Integration was not significantly associated with anxiety in males or females.  Assimilation mediated the positive association between discrimination and anxiety symptoms in males not females.  The results show that the mediation through assimilation is significant, albeit weak, only among males, while a moderate direct effect explains the association for females. |
|  |  |  |  |  | Daily discrimination |  |  |  |  | **Correlation coefficient = 0.226, p<0.05** | **Correlation coefficient = 0.473, p<0.01** |  |
|  |  |  |  |  | Assimilation (Time 2) |  |  |  |  | **Correlation coefficient = 0.259, p<0.01** | Correlation coefficient = 0.169 |  |
|  |  |  |  |  | Integration (Time 2) |  |  |  |  | Correlation coefficient = -0.014 | Correlation coefficient = -0.117 |  |
|  |  |  |  |  | Direct effect of discrimination |  |  |  |  | Unstandardised regression coefficient: 0.15 [CI: −0.01, 0.32] | **Unstandardised regression coefficient: 0.45 [0.27, 0.62]** |  |
|  |  |  |  |  | Indirect effect of discrimination through assimilation |  |  |  |  | **Unstandardised regression coefficient: 0.04 [CI: 0.002, 0.13]** | Unstandardised regression coefficient: 0.01 [CI: −0.01, 0.07] |  |
| González-Díez et al. (2017) (41) Spain | 16.97 (16-19), 55.8  High school | Longitudinal (Follow-up: 6 months and 1 year) | Baseline: 573  1 year follow-up: 550 | To analyse the role of social looming cognitive style as a mediator between parents’ emotional abuse and peer victimization and social anxiety symptoms. | Baseline Peer victimisation | 1 year follow-up: Social anxiety symptoms | | Social Anxiety Questionnaire for Adults (SAQ-A30) | No formal test for sex/gender difference | Unstandardised regression coefficient = 0.09, p = 0.05 | Unstandardised regression coefficient = -0.11, p = 0.07 | The pattern of predictive associations among variables was similar for males and females, however the associations were not statistically significant. |
|  |  |  |  |  | Baseline Parents' emotional abuse |  |  |  |  | Unstandardised regression coefficient = -0.12, p = 0.07 | Unstandardised regression coefficient = 0.10, p = 0.08 |  |
| Grapin et al. (2024) (42) USA | NR (NR), 78, Black university students | Cross-sectional | 278 | To explore the relations between ORD and mental health among Black undergraduates. To investigate gender differences in these relations. | ORD | Generalised anxiety | | Screen for Adult Anxiety Related Disorders (SCAARED) | Unstandardised regression coefficient = -0.108, Standardised regression coefficient = -0.097, p=0.145 | NR | NR | ORD was significantly related to social anxiety for women, but not for men. |
|  |  |  |  |  |  | Social anxiety | |  | **Unstandardised regression coefficient = -0.184, Standardised regression coefficient = -0.157, p=0.019** | Unstandardised regression coefficient = −0.093, p = .177, 95% CI: −0.229 to 0.042 | **Unstandardised regression coefficient = 0.091, p = 0.014, 95% CI: 0.019 to 0.163** |  |
| Hawes et al. (2020) (43) Australia | 17.7 (12-25), 59  High school and University  Caucasian/White Australians: 80%  Asian Australian: 15%  Australian first peoples/Torres Strait Islander/Pacific Islander: <1%)  Mix of other backgrounds: 5% | Cross-sectional | 763 | To test the notion that Appearance related social media preoccupation would be uniquely associated with more elevated symptoms of depression and social anxiety. To explore whether associations of Appearance related social media preoccupation with depression and social anxiety symptoms and appearance sensitivities are stronger in young women or in young men. | Appearance rejection sensitivity | Social anxiety symptoms | | Social Anxiety Scale for Adolescents (SAS-A) | “No gender differences” | **Correlation coefficient = 0.60, p<0.01** | **Correlation coefficient = 0.67, p<0.01** | Time spent on social media and social media intensity (emotional connectedness towards social media use were both positively associated with social anxiety symptoms in females but not in males, however the gender difference was only statistically significant for time spent on social media. |
|  |  |  |  |  | Appearance related Preoccupation |  |  |  | “No gender differences” | **Correlation coefficient = 0.49, p<0.01** | **Correlation coefficient = 0.56, p<0.01** |  |
|  |  |  |  |  | Social media time spent |  |  |  | **Test for sex/gender difference:**  **z = -2.07, p = .04** | Correlation coefficient = 0.03 | **Correlation coefficient = 0.18, p<0.01** |  |
|  |  |  |  |  | Social media intensity |  |  |  | “No gender differences” | Correlation coefficient = 0.06 | **Correlation coefficient = 0.20, p<0.01** |  |
|  |  |  |  |  | General social media preoccupation |  |  |  | “No gender differences” | **Correlation coefficient = 0.38, p<0.01** | **Correlation coefficient = 0.47, p<0.01** |  |
|  |  |  |  |  | Appearance related social media preoccupation (centred) × gender |  |  |  | “No interaction effects of AR preoccupation × gender  were significant” | NR | NR |  |
| Hellemans et al. (2019) (44) Canada | 20 (NR), 62.5,  University,  White: 57.4% Black: 9.7%  Arab/West Asian: 9.4%  Asian: 8.6%  South Asian: 4.9%  Indigenous: 2.3% South East Asian: 2.0%  Latin American/ Hispanic: 1.4%  Other: 4.2% | Cross-sectional | 1043 | To assess whether perceptions of risk associated with cannabis relates to cannabis use among young adults. Moreover, we explored whether current symptoms of anxiety were also related to problematic use, and the role of gender in this relationship. | Problematic cannabis use | Anxiety symptoms | | Beck Anxiety Inventory | **Test for sex/gender difference:**  **F (1, 1036)=6.99, p<0.01** | **Regression coefficient = 0.24, 95% CI: 0.33 to 0.44), p<0.05** | **Regression coefficient = 0.61, 95% CI: 4.19 to 8.05, p<0.001** | Gender moderated the positive association between problematic cannabis use and anxiety, such that the association was stronger for females, compared to males. |
| Herring et al. (2021) (45) Ireland | 23.2 (NR), 63.4 | Cross-sectional | 470 | To quantify the associations between physical activity and subclinical, or analogue  Generalized Anxiety Disorder, Social Anxiety Disorder and Panic Disorder symptoms and status. To explore potential  sex-related differences. | Physical activity | Generalised Anxiety disorder | | Psychiatric Diagnostic Screening Questionnaire (PDSQ) | No formal test for sex/gender difference | **Standardised regression coefficient = -0.708, 95%CI: -1.074 to -0.341, p<0.001** | Standardised regression coefficient = -0.058, 95%CI -0.909 to 0.793, p=0.894 | Physical activity was a protective factor for generalised anxiety disorder in males, but not females/  Physical activity was a protective factor for social anxiety disorder in girls but not boys. |
|  |  |  |  |  |  | Social anxiety disorder | | Psychiatric Diagnostic Screening Questionnaire (PDSQ) |  | Standardised regression coefficient = -0.226, 95%CI -1.117 to 0.665, p=0.619 | **Standardised regression coefficient = -0.689, 95%CI -1.078 to 0.300, p=0.001** |  |
|  |  |  |  |  |  | Panic disorder | | Psychiatric Diagnostic Screening Questionnaire (PDSQ) |  | Standardised regression coefficient = -0.443, 95%CI -1.206 to 0.320, p=0.255 | Standardised regression coefficient = -0.414, 95%CI -0.930 to 0.102, p=0.116 |  |
| Johannessen et al. (2017) (46) Norway | NR (16-18), 50,  Secondary school | Cross-sectional | 6238 | To examine the relationship between anxiety and depression symptoms and alcohol consumption in a representative population of Norwegian adolescents. To investigate possible sex differences regarding this relationship. | Early onset alcohol consumption x gender | Anxiety symptom level: 2^nd^ quartile | | Hopkins Symptom Checklist 25 (HSCL-25) | Interaction: p=0.01 | Reference | **OR: 1.2 (95%CI: 1.0–1.5)** | “The associations between symptoms of anxiety and depression and early onset of alcohol consumption were stronger for girls than for boys.” |
|  |  |  |  |  |  | Anxiety symptom level: 3^rd^ quartile | |  |  | OR = 1.0 (95%CI: 0.9–1.3) | **OR: 1.4 (95%CI: 1.1–1.8)** |  |
|  |  |  |  |  |  | Anxiety symptom level: 4^th^ quartile | |  |  | OR = 1.1 (95%CI: 0.9–1.3) | **OR: 1.6 (95%CI: 1.3–2.0)** |  |
| Kaltschik et al. (2022) (47) Austria | Current study: 16.65 (14-20), 77.4, School  Previous study: 16.5 (14-21), 70.1, School | Cross-sectional | Current study (Time 2): 616  Previous study (Time 1: 3052 | To study the relationship between smartphone usage and mental health scores and physical activity and mental health scores. | Time 2: Smartphone use | Generalised Anxiety disorder symptoms | | Generalized Anxiety Disorder scale (GAD-7) | No formal test for sex/gender difference | Correlation coefficient = 0.160 | **Correlation coefficient = 0.135, p<0.01** | Smartphone usage was a risk factor for anxiety in girls but not in boys. |
|  |  |  |  |  | Time 2: Physical activity |  |  |  |  | **Correlation coefficient = -0.213, p<0.05** | **Correlation coefficient = -0.192, p<0.01** |  |
|  |  |  |  |  | Time 1: Smartphone use |  |  |  |  | **Correlation coefficient = 0.416, p<0.01** | **Correlation coefficient = 0.236, p<0.01** |  |
|  |  |  |  |  | Time 1: Physical activity |  |  |  |  | **Correlation coefficient = -0.303, p<0.01** | **Correlation coefficient = -0.169, p<0.01** |  |
|  |  |  |  |  | Smartphone use: difference between Time 1 and Time 2 |  |  |  |  | **Z = 2.152, p=0.016** | Z = 1.615, p=0.053 |  |
|  |  |  |  |  | Physical activity: difference between Time 1 and Time 2 |  |  |  |  | Z = -0.738, p=0.23 | Z = 0.367, p=0.357 |  |
| Kim et al. (2021) (48)  USA | 22.1 (18.25), 51.9,  Community | Cross-sectional | 345 | To test whether positive and negative family factors moderate associations between discrimination and mental and physical health outcomes in a diverse, urban sample of emerging adults. | Discrimination x positive family environment x gender | Anxiety symptoms | | Generalized Anxiety Disorder scale (GAD-7) | “No moderation effect” | NR | NR | Gender moderated the interaction between discrimination and family hostility in predicting anxiety, such that family hostility buffered the association between discrimination and anxiety in women but not in men. |
|  |  |  |  |  | Discrimination x family hostility x gender |  |  |  | **Standardised Regression coefficient = -0.15 Unstandardised Regression coefficient = -0.27, p<0.01** | Regression coefficient =0.10, p>0>.10 | Regression coefficient= −0.45, p<0<.01 |  |
| Kornilaki et al. (2022) (49) Greece | 21.5 (NR), 83.5  University | Cross-sectional | 1018 | To examine the effect of quarantine on young adults by assessing depression, anxiety, stress and the experience of positive and negative affect. | Life disruption due to Covid-19 x gender | Anxiety symptoms | | Depression, Anxiety and Stress Scale-21 (DASS-21) | Unstandardised regression coefficient = 0.501, standardised regression coefficient = 0.090, p = 0.010 | NR | “The effect of  life disruption on anxiety was greater for women” | Sex/gender moderated the association between anxiety and life disruption due to Covid-19, such that the association was stronger in females. |
| Kouros et al. (2017) (50) USA | 19.82 (18-25), 83.1,  European American: 57%  Hispanic/Latino/Mexican American: 36.5%  African American: 0.8%, Asian: 2.5%  University | Cross-sectional | 118 | To examine sex differences in the relation between helicopter parenting and autonomy support on college students’ mental health and well-being | Helicopter Parenting x sex | Social anxiety symptoms | | Inventory for Depression and Anxiety Symptoms (IDAS) | Standardised Regression coefficient = -0.23 Unstandardised Regression coefficient = -0.11 | NR | NR | Sex/gender moderated the negative association between autonomy support and social anxiety, such that autonomy was a protective factor against social anxiety in males but not in females. |
|  |  |  |  |  | Autonomy support × sex |  |  |  | **Standardised Regression coefficient = -0.81 Unstandardised Regression coefficient = -0.63, p=0.019** | Negative association in males (p=0.002) | No association in females (p=0.1) |  |
| Laftman et al. (2024) (51) Sweden | T1: 15-16  T2: 17-18  T3: 20-21  58.4% females  Secondary school | Longitudinal | 2323 | To examine whether being bullied in grade 9 or 11 is associated with depression and anxiety symptoms at age 20–21. Potential differences by gender were investigated throughout. | Bullied neither in grade 9 nor 11 (reference) | Anxiety symptoms (age 20-21) | | Patient Health Questionnaire-4 (PHQ-4) | NA | OR = 1.00 | OR = 1.00 | Gender did not moderate the association between bullying and anxiety. |
|  |  |  |  |  | Bullied in grade 9 (age 15-16) only |  |  |  |  | **OR = 2.07, 95%CI: 1.01 to 4.24, p<0.05** | **OR = 1.64, 95%CI: 1.01 to 2.66, p<0.05** |  |
|  |  |  |  |  | Bullied in grade 11 (age 17-18) only |  |  |  |  | OR = 1.32, 95%CI 0.53 to 3.29 | OR = 1.50, 95%CI: 0.80 to 2.82 |  |
|  |  |  |  |  | Bullied in both grade 9 and 11 |  |  |  |  | OR = 0.37, 95%CI 0.05 to 2.85 | **OR = 2.37, 95%CI: 1.18 to 4.77, p<0.05** |  |
|  |  |  |  |  | Family type: 2 original parents (reference) |  |  |  |  | OR = 1.00 | OR = 1.00 |  |
|  |  |  |  |  | Family type: one parent |  |  |  |  | **OR = 1.98, 95%CI: 1.17 to 3.36, p<0.05** | OR = 1.32, 95%CI: 0.94 to 1.87 |  |
|  |  |  |  |  | Family type: shared residence |  |  |  |  | OR = 1.48, 95%CI: 0.90 to 2.43 | OR = 1.32, 95%CI: 0.94 to 1.84 |  |
|  |  |  |  |  | Family type: Other |  |  |  |  | OR = 0.67, 95%CI 0.16 to 2.71 | **OR = 2.04, 95%CI: 1.02 to 4.08, p<0.05** |  |
|  |  |  |  |  | Parental education: ≤ 2 years secondary or less |  |  |  |  | OR = 1.07, 95%CI: 0.58 to 1.97 | OR = 1.28, 95%CI: 0.84 to 1.94 |  |
|  |  |  |  |  | Parental education: ≥ 3 years secondary (reference) |  |  |  |  | OR = 1.00 | OR = 1.00 |  |
|  |  |  |  |  | Parental education: Tertiary |  |  |  |  | OR = 1.02, 95%CI: 0.65 to 1.58 | OR = 1.07, 95%CI: 0.78 to 1.47 |  |
|  |  |  |  |  | Parental country of birth: At least one in Sweden (reference) |  |  |  |  | OR = 1.00 | OR = 1.00 |  |
|  |  |  |  |  | Parental country of birth: Two parents outside Sweden |  |  |  |  | **OR = 2.20, 95%CI: 1.37 to 3.53, p<0.01** | OR = 1.18, 95%CI: 0.84 to 1.66 |  |
|  |  |  |  |  | Bullying x gender |  |  |  | χ2 = 2.99, p = 0.393 | NR | NR |  |
| Larsen et al. (2025)(52) Australia | Time 1: 12 Time 2: 15 Time: 17 Time: 19  50.6% females  School | Longitudinal (Follow-up: 3, 2, 2 years) | 245 | To investigate whether residential greenness  was associated with adolescent trajectories of anxiety, depression,  and externalizing behaviour in an Australian cohort. | Residential greenness x age x sex | Anxiety symptoms | | Beck Anxiety Inventory (BAI) | Unadjusted regression coefficient = 1.90, 95% CI: -1.93 to 5.73 Adjusted regression coefficient = 1.16, 95% CI: -2.75 to 5.06 | NR | NR | No evidence of an interaction between sex, age and residential greenness in predicting anxiety. |
| Leadbeater et al. (2014) (53) Canada | Baseline: 15.52 (NR), 51.66  T5: 24.18 (20-27), 70 | Longitudinal Baseline: 2003  Time 2:2005  Time 3: 2007  Time 4: 2009  Time 5: 2011 | Baseline: 662  Time 5: 465 | To assess concurrent and prospective associations between internalizing symptoms and peer victimization from adolescence to young adulthood. | Time 5: Relational victimization | Time 5: Anxiety symptoms | | Brief Child and Family Phone Interview (BCFPI) | “Sex differences …were not significant” | **Significant**  **(value not reported)** | **Significant**  **(value not reported)** | Relational victimization was a risk factor in females but not in males. This sex/gender difference was statistically significant. |
|  |  |  |  |  | Time 5: Physical victimization |  |  |  |  | Not significant (value not reported) | Not significant (value not reported) |  |
|  |  |  |  |  | Time 3: Relational victimization |  |  |  | Test for sex/difference:  χ2 = 723.14, p<0.001 | Not significant (value not reported) | **Standardised regression coefficient = 0.13, p < 0.01** |  |
|  |  |  |  |  | Time 3: Physical victimization |  |  |  | NR | Not significant (value not reported) | Not significant (value not reported) |  |
| Lee et al. (2022) (54) Taiwan | 24.6 (20-30), 50,  Taiwanese young adults | Cross-sectional | 100 | To examine the relationships of individual factors (gender, age, education level, sexual orientation, and age of identification of sexual orientation) and perceived family support with internalised sexual stigma and the moderating effects of gender. | Internalized Sexual Stigma: Social discomfort x gender | Anxiety symptoms | | State-Trait Anxiety Inventory (STAI-S) | **Interaction:**  **Regression coefficient = -0.414, p<0.05** | Standardised regression coefficient = 0.152, p = 0.252 | **Regression coefficient = 0.708, p < 0.001** | Sex/gender moderated the positive association between anxiety and internalised sexual stigma in gay and bisexual young people, such that internalised sexual stigma was a risk factor in females, but not in males. |
|  |  |  |  |  | Internalized Sexual Stigma: Sexuality x gender |  |  |  | Interaction:  Regression coefficient = 0.265 | NR | NR |  |
| Lin et al. (2020) (55)  Hong Kong | 20.27 (18-30),  53.9, University | Cross-sectional | 707 | To examine the relationships between perceived weight stigma, eating disturbances, and emotional distress across individuals with different self-perceived weight statuses and genders in Asian samples across two areas (Hong Kong and Taiwan). | Perceived weight stigma | Anxiety symptoms | | Hospital anxiety and depression scale (HADS) | No formal test for sex/gender difference | **Regression coefficient = 0.133, p<0.05** | **Regression coefficient = 0.217, p<0.001** | Positive association between anxiety and perceived weight stigma in males and females. |
| Maciel et al. (2020) (56) Australia | 18.87 (18-20), 63.05,  Ethnic minority: 15.42% Ethnic majority: 84.58% | Cross-sectional | 617 | To evaluate the impact of child sexual abuse on mental health and deviant behaviour in early adulthood. | Child Sexual Abuse - Multiple perpetrators | Anxiety symptoms | | Depression, Anxiety and Stress Scale-21 (DASS-21) | No formal test for sex/gender difference | Correlation coefficient = 0.01 | Correlation coefficient = -0.03 | Child sexual abuse where victims had family ties with the perpetrator was a risk factor of anxiety in females but not in males, suggesting that intrafamilial sexual abuse is more damaging to victims’ mental health than extrafamilial sexual abuse. |
|  |  |  |  |  | Child Sexual Abuse - Victim-Perpetrator relationship (familial/non-familial) |  |  |  |  | Correlation coefficient = -0.01 | **Correlation coefficient = 0.33, p<0.05** |  |
|  |  |  |  |  | Child Sexual Abuse - Oral, vaginal or anal penetration |  |  |  |  | Correlation coefficient = -0.08 | Correlation coefficient = 0.02 |  |
|  |  |  |  |  | Child Sexual Abuse - Forced |  |  |  |  | Correlation coefficient = -0.11 | Correlation coefficient = 0.28 |  |
|  |  |  |  |  | Child Sexual Abuse - Age of onset (Adolescence/Childhood) |  |  |  |  | Correlation coefficient = 0.30 | Correlation coefficient = 0.19 |  |
| Mar et al. (2024) (57) Spain | 15.57 (1-30), 48.7  19-24 subgroup | Cross-sectional | 609381 | To assess socioeconomic and gender mental health inequalities in adolescents and young adults using a population-based registry. | Medium-to-high SES | Anxiety diagnosis | | Registry information | NA | **Adjusted OR: 0.52, 95%CI: 0.50–0.54, p<0.01** | Reference | Compared to medium-to-high socioeconomic status females, medium-to-high SES males had a lower likelihood of anxiety, as did low SES males. |
|  |  |  |  |  | Low SES |  |  |  |  | **Adjusted OR: 0.85, 95%CI: 0.78–0.93, p<0.05** | **Adjusted OR: 1.62, 95%CI: 1.51–1.74, p<0.01** |  |
| Marshal et al. (2011) (58) USA | 17.2 (16-19), 100,  African American: 55%  European American: 39%  Multiracial or other race: 6% | Cross-sectional | 2064 | To examine substance use and mental health disparities among sexual minority girls and to determine whether disparities were larger for African American compared with European American girls. | Sexual minority | Anxiety symptoms | | Childhood Anxiety Related Disorders | NA | MA | **Standardised regression coefficient = 0.05, p<0.05** | There was a negative association between anxiety and age, and a positive association between sexual minority status and anxiety. |
|  |  |  |  |  | Age |  |  |  |  |  | **Standardised regression coefficient = -0.05, p<0.05** |  |
|  |  |  |  |  | Minority race |  |  |  |  |  | Standardised regression coefficient = 0.03 |  |
|  |  |  |  |  | Low parent education |  |  |  |  |  | **Standardised regression coefficient = 0.07, p<0.01** |  |
| Mayorga et al. (2018) (59) USA | 20.67 (18-25), 78.3  Latino university students | Cross-sectional | 448 | To examine difficulties in emotion regulation as an explanatory factor in the relation of acculturative stress with symptoms of depression, suicidality, social anxiety, and anxious arousal. | Age | Social anxiety symptoms | | Inventory for Depression and Anxiety Symptoms (IDAS) | No formal tests for sex/gender difference | Correlation coefficient = -0.125 | Correlation coefficient = 0.002 | Being a sexual minority, greater levels of financial strain are each associated with social anxiety symptoms in males and females. |
|  |  |  |  |  | Sexual minority status |  |  |  |  | **Correlation coefficient = 0.264, p<0.01** | **Correlation coefficient = 0.111, p<0.01** |  |
|  |  |  |  |  | Financial strain |  |  |  |  | **Correlation coefficient = 0.237, p<0.01** | **Correlation coefficient = 0.132, p<0.01** |  |
| McKinney et al. (2011) (60) USA | 19.22 (18-22), 69,  University  Caucasian: 76.2%  Hispanic: 9.7%  African American: 6.5%  Asian: 1.9%  Other: 3.6% | Cross-sectional | 526 | To investigate the relationships among parenting styles, discipline strategies and emerging adult emotional adjustment (e.g., self-esteem, depression, and anxiety). | Maternal bonding - care | Anxiety symptoms | | Manifest Anxiety Scale (MAS) | No formal tests for sex/gender difference | Correlation coefficient = -0.21 | **Correlation coefficient = 0.30, p<0.01** | For females, both paternal and maternal caring were protective factors for anxiety, whereas for males only paternal caring was a protective factor.  Maternal and paternal authoritative parenting styles were protective factors for anxiety in females, but not in males.  Both paternal and maternal overprotection was a risk factor for anxiety in females, whereas for males only paternal overprotection was a risk factor.  Maternal permissive parenting styles was a risk factor for anxiety in both males and females.  Maternal and paternal authoritarian parenting styles and non-violent, psychological aggression, corporeal punishment and severe assault discipline strategies were also risk factors for anxiety in males and females. |
|  |  |  |  |  | Maternal bonding - overprotection |  |  |  |  | Correlation coefficient = 0.17 | **Correlation coefficient = 0.27, p<0.01** |  |
|  |  |  |  |  | Maternal Parenting style - authoritative |  |  |  |  | Correlation coefficient = -0.12 | **Correlation coefficient = 0.23, p<0.01** |  |
|  |  |  |  |  | Maternal Parenting style - authoritarian |  |  |  |  | **Correlation coefficient = 0.22, p<0.01** | **Correlation coefficient = 0.29, p<0.01** |  |
|  |  |  |  |  | Maternal Parenting style - permissive |  |  |  |  | **Correlation coefficient = 0.35, p<0.01** | **Correlation coefficient = 0.26, p<0.01** |  |
|  |  |  |  |  | Maternal Parent discipline practice - nonviolent |  |  |  |  | **Correlation coefficient = 0.25, p<0.01** | **Correlation coefficient = 0.28, p<0.01** |  |
|  |  |  |  |  | Maternal Parent discipline practice - psychological aggression |  |  |  |  | **Correlation coefficient = 0.35, p<0.01** | **Correlation coefficient = 0.35, p<0.01** |  |
|  |  |  |  |  | Maternal Parent discipline practice - corporeal punishment |  |  |  |  | **Correlation coefficient = 0.33, p<0.01** | **Correlation coefficient = 0.18, p<0.01** |  |
|  |  |  |  |  | Maternal Parent discipline practice - severe assault |  |  |  |  | **Correlation coefficient = 0.25, p<0.01** | **Correlation coefficient = 0.16, p<0.01** |  |
|  |  |  |  |  | Paternal bonding - care |  |  |  |  | **Correlation coefficient = -0.25, p<0.01** | **Correlation coefficient = -0.25, p<0.01** |  |
|  |  |  |  |  | Paternal bonding - overprotection |  |  |  |  | **Correlation coefficient = 0.22, p<0.01** | **Correlation coefficient = 0.21, p<0.01** |  |
|  |  |  |  |  | Paternal Parenting style - authoritative |  |  |  |  | Correlation coefficient = -0.10 | **Correlation coefficient = -0.15, p<0.01** |  |
|  |  |  |  |  | Paternal Parenting style - authoritarian |  |  |  |  | **Correlation coefficient = 0.23, p<0.01** | **Correlation coefficient = 0.22, p<0.01** |  |
|  |  |  |  |  | Paternal Parenting style - permissive |  |  |  |  | Correlation coefficient = -0.08 | Correlation coefficient = -0.09 |  |
|  |  |  |  |  | Paternal Parent discipline practice - nonviolent |  |  |  |  | **Correlation coefficient = 0.21, p<0.01** | **Correlation coefficient = 0.23, p<0.01** |  |
|  |  |  |  |  | Paternal Parent discipline practice - psychological aggression |  |  |  |  | **Correlation coefficient = 0.27, p<0.01** | **Correlation coefficient = 0.26, p<0.01** |  |
|  |  |  |  |  | Paternal Parent discipline practice - corporeal punishment |  |  |  |  | **Correlation coefficient = 0.35, p<0.01** | **Correlation coefficient = 0.18, p<0.01** |  |
|  |  |  |  |  | Paternal Parent discipline practice - severe assault |  |  |  |  | **Correlation coefficient = 0.30, p<0.01** | **Correlation coefficient = 0.16, p<0.01** |  |
|  |  |  |  |  | Self-esteem |  |  |  |  | **Correlation coefficient = -0.66, p<0.01** | **Correlation coefficient = -0.57, p<0.01** |  |
| Minhas et al. (2013) (61) Canada | 23.84 (19-23),  Assigned female at birth: 58.4.  Gender: Cisgender-male: 41.4; Cisgender-Female: 56.9; Other: 1.69%  Ethnicity: White: 70.4% | Longitudinal (Follow-up: 2 weeks) | 473 | To examine changes in drinking and common mental health symptoms longitudinally. | Time x sex  (Changes in anxiety pre-COVID to intra-COVID) | Anxiety symptoms score | | Generalized Anxiety Disorder scale (GAD-7) | F/(χ2) = 6.71, p = 0.00988 | NR | **p<0.001** | Sex/gender moderated the association and time, such that there was an increase in anxiety in females, but not in males. |
|  |  |  |  |  |  | Anxiety threshold | |  | F/(χ2) = 1.78, p = 0.18 | NR | NR |  |
| Neblett et al. (2016) (62)  USA | 18.4 (18-19), 69, African American university students | Cross-sectional | 171 | To investigate the influence of gender and SES on the association between racial discrimination and mental health in a sample of African American young adults. | Racial discrimination frequency x maternal educational attainment x gender | Anxiety symptoms | | Symptom Checklist-90 | **F(1, 159) = 4.91, p = 0.028** | **Lower SES backgrounds: p<0.001** | **Higher SES backgrounds: p=0.002** | Evidence of sex/gender moderation was found, such that males from lower SES backgrounds and females from higher SES backgrounds were at greater risk of the anxiety associated with racial discrimination. |
| Pachankis et al. (2012) (63) USA | 20.56 (NR), 71.3,  Ethnicity:  White: 71.3%; Asian: 8.8%; Latino/Hispanic: 7.4%; Black/African American: 5.1%.  Gay and heterosexual men at university | Cross-sectional | 195 | To propose a path model investigating the  association of boyhood gender nonconformity  and parental disapproval of self-expression with  cognitive, affective, and behavioural experiences  of anxiety across the daily lives of young gay  men. | Gender nonconformity | Anxiety symptoms | | Positive and Negative Affect Scale (PANAS) | NA | Correlation coefficient = -0.02 | N/A | No evidence of an association between gender-nonconformity and anxiety.  Evidence of positive associations between anxiety and sexual orientation concealment, public self-consciousness and parent disapproval. |
|  |  |  |  |  | Parental disapproval |  |  |  |  | **Correlation coefficient = 0.21, p<0.05** |  |  |
|  |  |  |  |  | Sexual orientation concealment |  |  |  |  | **Correlation coefficient = 0.18, p<0.05** |  |  |
|  |  |  |  |  | Public self-consciousness |  |  |  |  | **Correlation coefficient = 0.28, p<0.01** |  |  |
|  |  |  |  |  | Direct path: Parental disapproval |  |  |  |  | **“Parental disapproval exhibited statistically significant direct paths to daily anxiety”** |  |  |
|  |  |  |  |  | Indirect path: Parental disapproval to anxiety through daily public self- consciousness and covering |  |  |  |  | **Regression coefficient = 0.02 (95% CI: 0.01 to 0.13)** |  |  |
|  |  |  |  |  | Indirect path: Public self-consciousness to anxiety through covering |  |  |  |  | **Regression coefficient = 0.07 (95% CI = 0.02 to 0.15)** |  |  |
| Perkins et al. (2022) (64)  USA | 19.59, 71% females. Black first year university students | Cross-sectional | 353 | To examine the moderating role of racial identity in the association between online and offline racial discrimination and psychological adjustment. | Offline discrimination x public regard x gender | Anxiety symptoms | | Generalized Anxiety Disorder Screener–Symptoms Scale | **Interaction:**  **Standardised regression coefficient = -0.24, Unstandardised regression coefficient = -0.21, p<0.01** | NR | **Regression coefficient = 0.28, p<0.001** | Evidence of sex/gender moderation was found such that public regard increased the association between offline discrimination and anxiety in black males but reduced the association in black females.  Sex/gender moderated the interaction between online discrimination and public regard in predicting anxiety, such that public regard was a protective factor against anxiety associated with the online discrimination in black females only. |
|  |  |  |  |  | Online discrimination x public regard x gender |  |  |  | **Interaction: Standardised regression coefficient = -0.27, Unstandardised regression coefficient = -0.97, p<0.05** | **Regression coefficient = 0.33, p < 0.001** | **Regression coefficient = 0.88, p=.001** |  |
| Poteat et al. (2014) (65) USA | High school students  Whole sample: 15.8 (NR), 55  Whole sample ethnicity:  White: 89.1%; Hispanic/ Latino: 2.9 %; Asian American: 2.5 %; African American: 2.1 %; ‘‘Other’’: 1.6 % | Longitudinal  Time 2: 7 months after baseline | Whole sample Baseline: 618  Heterosexual students: Baseline: 572  Time 2: 456 | To identify the short-term effects of homophobic victimization for heterosexual youth. | Baseline General victimisation | Baseline Anxiety symptoms | | Depression, Anxiety and Stress Scale-21 (DASS-21) | NA | **Correlation coefficient = 0.40, p<0.001** | **Correlation coefficient= 0.40, p<0.001** | Sex/gender moderation was found such that homophobic victimisation was a risk factor for males but not females. |
|  |  |  |  |  | Baseline Homophobic victimisation |  |  |  |  | **Correlation coefficient = 0.35, p<0.001** | **Correlation coefficient= 0.35, p<0.001** |  |
|  |  |  |  |  | Time 2 General victimisation |  |  |  |  | **Correlation coefficient= 0.32, p<0.001** | **Correlation coefficient= 0.32, p<0.001** |  |
|  |  |  |  |  | Time 2 Homophobic victimisation |  |  |  |  | Correlation coefficient= -0.04 | Correlation coefficient= -0.04 |  |
|  |  |  |  |  | Baseline: General victimisation | Time 2 Anxiety symptoms | | Depression, Anxiety and Stress Scale-21 (DASS-21) |  | **Correlation coefficient= 0.26, p<0.001** | **Correlation coefficient= 0.26, p<0.001** |  |
|  |  |  |  |  | Baseline: Homophobic victimisation |  |  |  |  | **Correlation coefficient= 0.31, p<0.001** | **Correlation coefficient= 0.31, p<0.001** |  |
|  |  |  |  |  | Time 2 General victimisation |  |  |  |  | **Correlation coefficient= 0.28, p<0.001** | **Correlation coefficient= 0.28, p<0.001** |  |
|  |  |  |  |  | Time 2 Homophobic victimisation |  |  |  |  | **Correlation coefficient= 0.25, p<0.001** | **Correlation coefficient= 0.25, p<0.001** |  |
|  |  |  |  |  | Baseline General victimisation x gender | Baseline: Anxiety symptoms | | Depression, Anxiety and Stress Scale-21 (DASS-21) | “There were no significant interaction effects” | NR | NR |  |
|  |  |  |  |  | Baseline: Homophobic victimisation x gender |  |  |  |  | NR | NR |  |
|  |  |  |  |  | Baseline: General victimisation x gender | Time 2 Anxiety symptoms | | Depression, Anxiety and Stress Scale-21 (DASS-21) | **Interaction:**  **Regression coefficient =0.35, p<0.01** | Regression coefficient = -0.05, p = 0.49) | Regression coefficient =0.06,p=0.27 |  |
|  |  |  |  |  | Baseline: Homophobic victimisation x gender |  |  |  | **Interaction:**  **Regression coefficient =-0.28, p<0.05** | **Regression coefficient = 0.24, p<0.001** | Regression coefficient = -0.05, p = 0.28 |  |
| Ranta et al. (2013) (66) Finland | Baseline: 15.5 (15-16) 49.1  Time2: 17.6 (17-18)  Secondary school students. | Longitudinal (Time 2: 2 years after baseline) | Baseline: 3278  Time 2: 2070 | To examine longitudinal associations between direct victimization and relational peer victimization and social phobia among adolescents from 15 to 17 years of age. | Crude association: Baseline Direct victimization | Time 2 Social Phobia | | Social Phobia Inventory (SPIN) | No formal tests for sex/gender difference or moderation | **OR = 2.8, p<0.05** | **OR = 2.8, p<0.05** | Relational victimisation at age 15 was a risk factor for social phobia at age 17 in females, but not in males.  Direct victimisation at age 15 was a risk factor for social phobia at age 17 in males, but not in females. |
|  |  |  |  |  | Crude association: Baseline Relational victimization |  |  |  |  | OR = 1.0 | **OR = 6.0, p<0.001** |  |
|  |  |  |  |  | Logistic regression model 1: Baseline Direct victimization |  |  |  |  | **OR = 3.0, p<0.01** | OR = 2.4 |  |
|  |  |  |  |  | Logistic regression model 1: Baseline Relational victimization |  |  |  |  | OR = 0.5 | **OR = 5.5, p<0.001** |  |
|  |  |  |  |  | Logistic regression model 2 (Social phobia and depression controlled for): Baseline Direct victimization |  |  |  |  | **OR = 2.8, p<0.05** | OR = 1.4 |  |
|  |  |  |  |  | Logistic regression model 2: Baseline Relational victimization |  |  |  |  | OR = 0.4 | OR = 2.3 |  |
|  |  |  |  |  | Logistic regression model 3 (Family moving and parental unemployment additionally controlled for): Baseline Direct victimization |  |  |  |  | **OR = 2.6, p<0.05** | OR = 1.2 |  |
|  |  |  |  |  | Logistic regression model 3: Baseline Relational victimization |  |  |  |  | OR = 0.3 | **OR = 2.8, p<0.05** |  |
|  |  |  |  |  | Time 2 family moved within 12 months |  |  |  |  | OR = 1.3 | OR = 1.4 |  |
|  |  |  |  |  | Time 2 parental unemployment in last 12 months |  |  |  |  | **OR = 2.2, p<0.05** | OR = 0.5 |  |
| Reed et al. (2019) (67) USA | 17 (15-19), 100, health clinic located in a low-income, urban neighbourhood of San Diego County | Cross-sectional | 159 | To assess the prevalence, perpetrators, and consequences of CSH among adolescent female | CSH | Anxiety | | Single item asking participants how often they felt worried, tense or anxious in the past 30 days on a Likert-type scale | NA | NA | **Unadjusted OR=6.5, 95% CI: 3.0 to 14.3, p<0.0001; Adjusted OR=5.3, 95%CI: 2.1 to 13.0, p<0.001** | Participants who reported CSH had a higher odds of feeling anxious (past 30 days). |
| Rieselbach et al. (2014) (68) USA | Baseline: 16.6 (13-18)  Time 2: 22.8 (19-33) | Longitudinal | 2845 | To clarify evidence for anxiety-specific associations with substance use, examining sex and developmental period (adolescence vs. adulthood) as potential moderators that may help explain conflicting results in the literature. | General substance use  Ages 16-18 subgroup | Anxiety symptoms | | Diagnostic Interview Schedule IV (DIS-IV) | NA | Regression coefficient = -0.04 | **Regression coefficient = -0.15, p<0.05** | Substance use was a protective factor against anxiety in adolescent females, but not in males. |
|  |  |  |  |  | General substance use  Ages 21-23 subgroup |  |  |  |  | Regression coefficient = 0.02 | **Regression coefficient = 0.13, p<0.05** |  |
|  |  |  |  |  | **Adolescent subgroup: 16.6 (13-18) years** | | | | | | |  |
|  |  |  |  |  | General substance use | Anxiety symptoms | | Diagnostic Interview Schedule IV (DIS-IV) | NA | Regression coefficient = -0.02 | **Regression coefficient = -0.15, p<0.05** |  |
|  |  |  |  |  | Past year tobacco use |  |  |  |  | Regression coefficient = 0.00, 95%CI: -0.07 to 0.07 | **Regression coefficient= -0.16, 95%CI:** -0.24 to -0.09, **p<0.05** |  |
|  |  |  |  |  | Past year alcohol use |  |  |  |  | Regression coefficient =-0.04, 95%CI: -0.10 to 0.02 | **Regression coefficient= -0.12, 95%CI:** -0.18 to -0.06, **p<0.05,** |  |
|  |  |  |  |  | Past year cannabis use |  |  |  |  | Regression coefficient = -0.05, 95%CI: -0.14 to 0.04 | **Regression coefficient= -0.12, 95%CI:** -0.21 to -0.04, **p<0.05** |  |
|  |  |  |  |  | Past year other use |  |  |  |  | Regression coefficient = 0.08, 95%CI: -0.03 to 0.18 | Regression coefficient= -0.03, 95%CI: -0.14 to 0.09 |  |
|  |  |  |  |  | Substance use x sex |  |  |  | **Test for sex/gender difference:**  **χ2(1) = 4.15, p < 0.05** | NR | NR |  |
|  |  |  |  |  | **Adult subgroup: 22.8 (19-33) years** | | | | | | |  |
|  |  |  |  |  | General substance use | Anxiety symptoms | | Diagnostic Interview Schedule IV (DIS-IV) | NA | Regression coefficient = 0.05 | **Regression coefficient = 0.12, p<0.05** |  |
|  |  |  |  |  | Past year tobacco use |  |  |  |  | **Regression coefficient =.08, 95%CI: 0**.02 to 0.14, **p<0.05** | **Regression coefficient = 0.06, 95%CI: 0**.01 to 12, **p<0.05,** |  |
|  |  |  |  |  | Past year alcohol use |  |  |  |  | Regression coefficient = -0.03, **95%CI:** -0.10 to 0.04 | Regression coefficient= 0.00, **95%CI**:-0.05 to 0.06 |  |
|  |  |  |  |  | Past year cannabis use |  |  |  |  | Regression coefficient =.05, **95%CI:** -0.01 to 0.12 | **Regression coefficient= 0.10, 95%CI**: 0.03 to 0.16, **p<0.05** |  |
|  |  |  |  |  | Past year other use |  |  |  |  | Regression coefficient =.01, **95%CI:** -0.05 to 0.09 | **Regression coefficient= 0.16, 95%CI: 0**.08 to 0.22, **p<0.05** |  |
|  |  |  |  |  | Substance use x sex |  |  |  | Test for sex/gender difference:  χ2(1) = 0.65, p = 0.42 |  |  |  |
|  |  |  |  |  | Adolescent substance use |  |  |  | NR | Regression coefficient: 0**.18, 95%CI: 0**.06 to 0.30, p<0.05 | Regression coefficient: 0.26, **95%CI: 0**.16 to 0.36, p<0.05 |  |
| Sares-Jaske et al. (2023) (69) Finland | 16.2 (NR), 50.9, Secondary school | Cross-sectional | 152880 | To explore whether being bullied at school is associated with several poor mental health and wellbeing outcomes in different transgender and cisgender groups. | **Gender stratified logistic regression -** *Reference group: no experiences of being bullied* | | | | | | | In the gender stratified logistic regression bullying had a greater positive association with anxiety in cisgender boys. The 95% confidence interval for the association for cisgender boys did not overlap of those for other gender groups, indicating a statistically significant gender difference at the 5% level.  In the gender interaction logistic regression bullying had a greater positive association with social anxiety in transmasculine young people, when compared to. The 95% confidence intervals for the transmasculine young people did not overlap of those for other gender groups, indicating evidence at the 5% level of a gender difference. |
|  |  |  |  |  | No experience of being bullied | Generalised Anxiety disorder symptoms | | Generalized Anxiety Disorder scale (GAD-7) |  | Cisboys: 1 (ref.)  Transmasculine youth**:** 1 (ref.) | Cisgirls: 1 (ref.)  Transfeminine youth: 1 (ref.) |  |
|  |  |  |  |  | Experience of being bullied: less frequently |  |  |  |  | **Cisboys: OR = 2.97 (95% CI: 2.75 to 3.20)**  **Transmasculine youth: OR = 1.85 (95% CI: 1.61 to 2.12)** | **Cisgirls: OR = 2.39 (95% CI: 2.29 to 2.49)**  **Transfeminine youth: OR = 2.16 (95% CI: 1.57 to 2.98)** |  |
|  |  |  |  |  | Experience of being bullied: At least once a week |  |  |  |  | **Cisboys: OR = 6.47 (95% CI: 3.77 to 4.46)**  **Transmasculine youth: OR = 2.84 (95% CI: 2.26 to 3.57)** | **Cisgirls: OR = 4.10 (95% CI: 3.77 to 4.46)**  **Transfeminine youth: OR = 3.60 (95% CI: 2.54 to 5.11)** |  |
|  |  |  |  |  | No experience of being bullied | Social anxiety | | Social Phobia Inventory (Mini-SPIN) |  | Cisboys: 1 (ref.)  Transmasculine youth**:** 1 (ref.) | Cisgirls: 1 (ref.)  Transfeminine youth: 1 (ref.) |  |
|  |  |  |  |  | Experience of being bullied: less frequently |  |  |  |  | **Cisboys: OR = 2.24 (95% CI: 2.13 to 2.36)**  **Transmasculine youth: OR = 1.41 (95% CI: 1.22 to 1.64)** | **Cisgirls: OR = 1.78 (95% CI: 1.71 to 1.85)**  **Transfeminine youth: OR = 1.55 (95% CI: 1.16 to 2.06)** |  |
|  |  |  |  |  | Experience of being bullied: At least once a week |  |  |  |  | **Cisboys: OR = 3.26 (95% CI: 2.98 to 3.57)**  **Transmasculine youth: OR = 1.31 (95% CI: 1.04 to 1.65)** | **Cisgirls: OR = 2.59 (95% CI: 2.37 to 2.82)**  **Transfeminine youth: OR = 2.04 (95% CI: 1.48 to 2.80)** |  |
|  |  |  |  |  | **Gender interaction logistic regression** | | | | | | |  |
|  |  |  |  |  | *Reference group: cisboys with no bullying experiences* | | | | | | |  |
|  |  |  |  |  | No experience of being bullied | Generalised Anxiety disorder symptoms | | Generalized Anxiety Disorder scale (GAD-7) | NA | Cisboys: 1 (ref.)  **Transmasculine youth: OR = 17.2 (95% CI: 15.9 to 18.5)** | **Cisgirls: OR = 6.01 (95% CI: 5.76 to 6.26)**  **Transfeminine youth: OR = 4.65 (95% CI: 3.90 to 5.54)** |  |
|  |  |  |  |  | Experience of being bullied: less frequently |  |  |  |  | **Cisboys: OR = 2.82 (95% CI: 2.61 to 3.04)**  **Transmasculine youth: OR = 32.7 (95% CI: 29.0 to 37.0)** | **Cisgirls: OR = 14.5 (95% CI: 13.7-15.3)**  **Transfeminine youth: OR = 9.49 (95% CI: 7.31 to 12.3)** |  |
|  |  |  |  |  | Experience of being bullied: At least once a week |  |  |  |  | **Cisboys: OR = 6.08 (95% CI: 5.46 to 6.77)**  **Transmasculine youth: OR = 50.8 (95% CI: 40.8 to 63.3)** | **Cisgirls: OR = 25.0 (95% CI: 22.9 to 27.4)**  **Transfeminine youth: OR = 15.9 (95% CI: 12.0 to 21.1)** |  |
|  |  |  |  |  | No experience of being bullied | Social anxiety | | Social Phobia Inventory (Mini-SPIN) | NA | **Cisboys: 1 (ref.)**  **Transmasculine youth: OR = 8.81 (95% CI: 8.19 to 9.47)** | **Cisgirls: OR = 3.28 (95% CI: 3.20-3.37)**  **Transfeminine youth: OR = 2.84 (95% CI: 2.47 to 3.27)** |  |
|  |  |  |  |  | Experience of being bullied: less frequently |  |  |  |  | **Cisboys: OR = 2.15 (95% CI: 2.04 to 2.26)**  **Transmasculine youth: OR = 13.0 (95% CI: 11.5 to 14.8)** | **Cisgirls: OR = 5.96 (95% CI: 5.71-6.11)**  **Transfeminine youth: OR = 4.05 (95% CI: 3.18 to 5.15)** |  |
|  |  |  |  |  | Experience of being bullied: At least once a week |  |  |  |  | **Cisboys: OR = 3.11 (95% CI: 2.84-3.40)**  **Transmasculine youth: OR = 12.2 (95% CI: 9.86 to 15.2)** | **Cisgirls: OR = 8.74 (95% CI: 8.00-9.54)**  **Transfeminine youth: OR = 5.25 (95% CI: 3.98 to 6.91)** |  |
|  |  |  |  |  | *Reference group: cisboys with less frequent bullying experiences* | | | | | | |  |
|  |  |  |  |  | Experience of being bullied: less frequently | Generalised Anxiety disorder symptoms | | Generalized Anxiety Disorder scale (GAD-7) |  | Cisboys: 1 (ref.)  **Transmasculine youth: OR = 11.6 (95% CI: 10.2 to 13.3)** | **Cisgirls: OR = 5.15 (95% CI: 4.78-5.54)**  Transfeminine youth: OR = 3.37 (95% CI: 2.58 to 4.40) |  |
|  |  |  |  |  | Experience of being bullied: less frequently | Social anxiety | | Social Phobia Inventory (Mini-SPIN) |  | Cisboys: 1 (ref.)  **Transmasculine youth: OR = 6.07 (95% CI: 5.30 to 6.94)** | **Cisgirls: OR = 2.78 (95% CI: 2.62 to 2.95) Transfeminine youth: OR = 1.88 (95% CI: 1.48 to 2.41)** |  |
|  |  |  |  |  | Reference group: cisboys with bullying experiences at least once a  week | | | | | | |  |
|  |  |  |  |  | Experience of being bullied: At least once a week | Generalised Anxiety disorder symptoms | | Generalized Anxiety Disorder scale (GAD-7) |  | Cisboys: 1 (ref.)  **Transmasculine youth: OR = 8.36 (95% CI: 6.59 to 10.6)** | **Cisgirls: OR = 4.12 (95% CI: 3.61 to 4.69)**  **Transfeminine youth: OR = 2.61 (95% CI: 1.94 to 3.52)** |  |
|  |  |  |  |  | Experience of being bullied: At least once a week | Social anxiety | | Social Phobia Inventory (Mini-SPIN) |  | **Cisboys: 1 (ref.)**  **Transmasculine youth: OR = 3.94 (95% CI: 3.13 to 4.98)** | **Cisgirls: OR = 2.81 (95% CI: 2.49 to 3.18)**  **Transfeminine youth: OR = 1.69 (95% CI: 1.27 to 2.25)** |  |
| Schiffrin et al. (2019) (70) USA | 19.59 (18-25), 73.1, University | Cross-sectional | 446 | To investigate whether parents’ and children’s gender affected how helicopter parenting impacted emerging adults’ well-being. To examine whether there were gender differences in the effects of helicopter parenting on emerging adults’ well-being. | Indirect effect of maternal  helicopter parenting  on anxiety through emerging adults’ autonomy  (direct effect not reported) | Anxiety symptoms | | Hospital anxiety and depression scale (HADS) | No formal test for sex/gender difference | **Regression coefficient = 0.03, 95%CI: 0.006 to 0.05, p=0.02** | **Regression coefficient = 0.04, 95%CI: 0.02 to 0.06, p<0.001** | Maternal helicopter parenting was a risk factor for anxiety in males and females. |
|  |  |  |  |  | Indirect effect of paternal  helicopter parenting  on anxiety through emerging adults’ autonomy  (direct effect not reported) |  |  |  |  | Regression coefficient = 0.001, 95%CI: -0.006 to 0.01, p=0.76 | Regression coefficient = <0.01, 95%CI: -0.003 to 0.002, p=0.92 |  |
| Sebokova et al. (2016) (71) Slovakia | 17.35 (14-21), 63.6,  High schools and secondary schools | Cross-sectional | 294 | To examine the association between self-consciousness and internalizing problems in adolescents, and to analyse moderating effects of family dimension. | Loneliness | Social anxiety symptoms | | The Scale of Classical Fears and Stage Fright, Social situational fears (KSAT) | No formal test for sex/gender difference | Correlation coefficient = 0.16 | Correlation coefficient = 0.09 | In the ‘boys’ subgroup, family cohesion moderated the association between with self-consciousness and social anxiety such that high levels of cohesion increased the association between public self-consciousness and social anxiety. In the ‘girls subgroup’, family adaptability (ability to respond to stressful situations) moderated the association between public self-consciousness and social anxiety, such that adaptability reduced the association between self-consciousness and social anxiety.  In the ‘girls subgroup’, communication moderated the association between public self-consciousness and social anxiety, such that communication buffers the association between self-consciousness and social anxiety. |
|  |  |  |  |  | Communication |  |  |  |  | Correlation coefficient = 0.04 | Correlation coefficient = -0.08 |  |
|  |  |  |  |  | Cohesion |  |  |  |  | Correlation coefficient = -0.16 | Correlation coefficient = -0.03 |  |
|  |  |  |  |  | Adaptability |  |  |  |  | Correlation coefficient = -0.06 | Correlation coefficient = -0.06 |  |
|  |  |  |  |  | Private self-consciousness |  |  |  |  | Correlation coefficient = 0.06 | Correlation coefficient = 0.09 |  |
|  |  |  |  |  | Public self-consciousness |  |  |  |  | Correlation coefficient = 0.15 | Correlation coefficient = 0.14 |  |
|  |  |  |  |  | Age |  |  |  |  | Correlation coefficient = -0.08 | Correlation coefficient = -0.07 |  |
|  |  |  |  |  | Adaptability x Public self-consciousness |  |  |  |  | Standardised regression coefficient = 0.20 | **Interaction: Standardised regression coefficient = -0.15, p<0.05**  **Low levels of**  **family adaptability: Regression coefficient = 0.26, *p*<0.05)**  Medium levels of  family adaptability:  Regression coefficient = 0.11, *p*>0.05  High levels of  family adaptability:  Regression coefficient =  -0.03, *p*>0.05 |  |
|  |  |  |  |  | Communication x Public self-consciousness |  |  |  |  | Standardised regression coefficient = -0.04 | **Interaction: Standardised regression coefficient = -0.18, p<0.05**  **Low levels of communication:**  **Regression coefficient = 0.26, *p* < .01**  Medium levels of communication:  Regression coefficient = 0.10, *p* > 0.05  High levels of communication:  Regression coefficient = -0.05, *p*>0.05 |  |
|  |  |  |  |  | Cohesion x Public self-consciousness |  |  |  |  | **Interaction:**  **Standardised regression coefficient = 0.23, p<0.05**  **High levels of family cohesion:**  **Regression coefficient**  **= 0.36, *p*<0.05)**  Medium levels of family cohesion:  Regression coefficient  = 0.15, *p* >.05  Low levels of family cohesion:  Regression coefficient  = -0.06, *p* >.05 | Standardised regression coefficient = -0.10 |  |
| Shorey et al. (2015) (72) USA | 21.56 (18-25), 34.5,  Non-Hispanic Caucasian: 96%  African American: 2.7%  Hispanic: 1.4%  Residential substance use treatment centre | Cross-sectional | 148 | The present study examined the relation between the moment-to-moment attention facet of mindfulness and anxiety among young adults in a residential substance use treatment program. | Alcohol use | Generalised Anxiety disorder | | Psychiatric Diagnostic Screening Questionnaire (PDSQ) | No formal test for sex/gender difference | Correlation coefficient = 0.15 | Correlation coefficient = 0.08 | No evidence of an association with anxiety. |
|  |  |  |  |  | Drug Use Disorders |  |  |  |  | Correlation coefficient = 0.18 | Correlation coefficient = 0.22 |  |
|  |  |  |  |  | Age |  |  |  |  | Correlation coefficient = 0.11 | Correlation coefficient = 0.22 |  |
|  |  |  |  |  | Education |  |  |  |  | Correlation coefficient = -0.07 | Correlation coefficient = -0.00 |  |
| Skogen et al. (2021) (73) Norway | 17.1 (NR), 42  High school | Cross-sectional | 513 | To investigate the association between focus on social media self-presentation and mental health and quality of life among adolescents. | Focus on self-presentation on social media | Anxiety symptoms | | Generalized Anxiety Disorder scale (GAD-7) | No formal test for sex/gender difference | **Regression coefficient: 0.35, p < 0.001** | **Regression coefficient: 0.38, p < 0.001** | Focus on self-presentation on social media was positively associated with anxiety in males and females. |
| Skogen et al. (2014) (74) Norway | 17.9 (17-19), 54.3  High school | Cross-sectional | 9203 | To investigate the cross-sectional relationship between alcohol and illicit drug use and alcohol and drug problems and mental health among adolescents. We also investigated any interactions with age and gender on the associations of interest. | Illicit drug use | Anxiety symptoms | | Screen for Child Anxiety Related Emotional Disorders (SCARED) | **Interaction: p=0.021** | **OR=2.44, (CI 95%: 1.57 to 3.78)** | OR=1.30 (CI 95%: 0.98 to 1.73) | Illicit drugs and excessive alcohol consumption were associated with increased symptoms of anxiety for boys but not for girls. |
|  |  |  |  |  | Excessive Alcohol consumption |  |  |  | **Interaction: p<0.001** | **OR=4.36 (CI 95%: 2.52 to 7.57)** | OR=1.23 (CI 95%: 0.87 to 1.73) |  |
| Stearns et al. (2021) (75) USA | 19.82 (18-25), 67.7,  University  Ethnicity:  White: 66.7%  Black: 24.74%  Latino: 1.8%  Asian: 2.6%  Other: 2.5% | Cross-sectional | 1002 | To examine if personal religiosity moderated the relationship between parent and emerging adult offspring anxiety symptoms. | Religiosity - Emerging adult private (e.g., I try to live my life according to my religious beliefs) | Anxiety symptoms | | Adult Self Report and Adult Behaviour Checklist | “No significant gender differences were found  between sons and daughters when examining the relationship between parental and emerging adult anxiety symptoms or emerging adult anxiety symptoms and religiosity" | Correlation coefficient = 0.01 | Correlation coefficient = 0.03 | Social Support and conservatism were protective factors for anxiety in females, but not in males.  There was evidence of sex/gender differences between males and females regarding the interaction between perceived maternal anxiety symptoms and religiosity in predicting anxiety. This interaction was statistically significant in females, such that religiosity increased the positive association between maternal and daughters’ anxiety in females. |
|  |  |  |  |  | Religiosity - Emerging adult coping (e.g., I find comfort in my religion or spirituality) |  |  |  |  | Correlation coefficient = 0.03 | Correlation coefficient = -0.01 |  |
|  |  |  |  |  | Religiosity - Emerging adult social support (e.g., I consider myself active in my faith or church) |  |  |  |  | Correlation coefficient = 0.02 | **Correlation coefficient = -0.13, p<0.01** |  |
|  |  |  |  |  | Religiosity - Emerging adult conviction (e.g., I will always believe in a divine being/God) |  |  |  |  | Correlation coefficient = 0.05 | Correlation coefficient = 0.01 |  |
|  |  |  |  |  | Religiosity - Emerging adult conservatism (e.g., I strictly follow my religious beliefs in regard to my appearance) |  |  |  |  | Correlation coefficient = 0.02 | **Correlation coefficient = -0.17, p<0.01** |  |
|  |  |  |  |  | Maternal anxiety |  |  |  |  | **Correlation coefficient = 0.35, p<0.01** | **Correlation coefficient = 0.42, p<0.01** |  |
|  |  |  |  |  | Paternal anxiety |  |  |  |  | **Correlation coefficient = 0.34, p<0.01** | **Correlation coefficient = 0.45, p<0.01** |  |
|  |  |  |  |  | Maternal anxiety x Emerging adult religiosity |  |  |  | **Test for sex/gender difference:**  **Z = 3.24, p<0.001** | Interaction: Standardised coefficient = 0.05  **Low emerging adult religiosity:**  **t(322) = −3.06, p = 0.002**  **High emerging adult religiosity:**  **t(322) = 4.28, p = 0.001** | **Interaction:**  **Standardised coefficient = 0.10, p<0.05**  **Low emerging adult religiosity:**  **t(676) = 3.50, p = 0.001**  High emerging adult religiosity:  *t*(676) = −1.30, *p* = 0.195 |  |
|  |  |  |  |  | Paternal anxiety x Emerging adult religiosity |  |  |  | NR | Interaction**:** Standardised coefficient = 0.11  Low emerging adult religiosity**:**  t(322) = −1.61, p = 0.108  **High emerging adult religiosity:**  **t(322) = 2.04, p = 0.042** | **Interaction:**  **Standardised coefficient = -0.18, p<0.05**  **Low emerging adult religiosity:**  **t(676) = 3.08, p = 0.002**  **High emerging adult religiosity:**  **t(676) = −2.55, p = 0.011** |  |
| Surprenant et al. (2025) (76) Canada | 17.1 (16-21), 59,  Caucasian:  Women: 71.8% Men: 76.6%  African, Afro-American: Women: 9.8% Men: 8.6%  Asian:  Women: 4.6% Men: 3.5%  Hispanic, Latino-American: Women: 3.2% Men: 3.0%  Other:  Women: 10.6% Men: 8.4%  Colleges | Cross-sectional study | 2165 | To estimate associations between lifestyle habits, sociodemographic characteristics,  health status, and positive mental health (i.e., flourishing, languishing, moderate) and anxiety and depression  symptoms in postsecondary students. | Screen time (weekdays) | Anxiety symptoms | | Hospital Anxiety and Depression Scale (HADS) | No formal tests for sex/gender difference | OR = 0.98, 95%CI: 0.89 to 1.09 | OR = 1.06, 95%CI: 0.99 to 1.13 | Evidence of:  A positive association between time doing homework and anxiety in females (p<0.001), but not males (p-value not reported).  A positive association between a perception of sufficient income and anxiety in females (p<0.05), but not males (p-value not reported).  A positive association between a very poor/poor perceived of sufficient income and anxiety in females (p<0.01), and males (p<0.001).  A positive association between having a disability or health problem and anxiety in females (p<0.001), and males (p<0.05). |
|  |  |  |  |  | Screen time (weekend) |  |  |  |  | OR = 1.02, 95%CI: 0.93 to 1.12 | OR = 0.99, 95%CI: 0.93 to 1.05 |  |
|  |  |  |  |  | Physical activity (Moderate to vigorous) |  |  |  |  | OR = 1.00, 95%CI: 1.00 to 1.00 | OR = 1.00, 95%CI: 1.00 to 1.00 |  |
|  |  |  |  |  | In-person social interaction (Never) |  |  |  |  | OR = 0.74, 95%CI: 0.25 to 2.17 | OR = 1.78, 95%CI: 0.79 to 4.00 |  |
|  |  |  |  |  | In-person social interaction (Rarely (1 time/month)) |  |  |  |  | OR = 1.63, 95%CI: 0.86 to 3.08 | OR = 0.97, 95%CI: 0.65 to 1.50 |  |
|  |  |  |  |  | In-person social interaction (Occasionally (1 time/week)) |  |  |  |  | OR = 1.19, 95%CI: 0.73 to 1.95 | OR = 0.94, 95%CI: 0.71 to 1.25 |  |
|  |  |  |  |  | In-person social interaction (Frequently (>2 times/week)) |  |  |  |  | Reference | Reference |  |
|  |  |  |  |  | Homework |  |  |  |  | OR = 1.01, 95%CI: 0.98 to 1.04 | **OR = 1.04, 95%CI: 1.02 to 1.05, p<0.001** |  |
|  |  |  |  |  | Perceived socioeconomic status (Very poor/poor) |  |  |  |  | **OR = 2.85, 95%CI: 1.58 to 5.15, p<0.001** | **OR = 1.85, 95%CI: 1.20 to 2.87), p<0.01** |  |
|  |  |  |  |  | Perceived socioeconomic status (Sufficient income) |  |  |  |  | OR = 1.21, 95%CI: 0.73 to 1.97 | **OR = 1.41, 95%CI: 1.07 to 1.84), p<0.05** |  |
|  |  |  |  |  | Perceived socioeconomic status (Affluent) |  |  |  |  | Reference | Reference |  |
|  |  |  |  |  | Health status: Disability or health problem (No) |  |  |  |  | Reference | Reference |  |
|  |  |  |  |  | Health status: Disability or health problem (Yes) |  |  |  |  | **OR = 2.21, 95%CI: 1.09 to 4.45, p<0.05** | **OR = 2.23, 95%CI: 1.47 to 3.36, p<0.001** |  |
| Tao et al. (2025) (77) USA | 20.2 (18-24), 100, Individuals assigned female at birth who self-identified as Asian, Black, Hispanic or Latina.  32.74% Asian, 33.92% Black, and 33.33% Hispanic or Latina; 68.14% identified as straight or heterosexual  Community | Cross-sectional | 339 | To extend understanding of the role of close friendships in the lives of young women of colour by examining associations among friendship communications  specific to gendered racism and the relationship between  exposure to in-person and social media gendered racial  discrimination and mental health. | Exposure to discrimination | Anxiety symptoms | | Generalized Anxiety Disorder Screener (GAD-7) | NA |  | **Correlation coefficient = 0.21, p < 0.001** | Evidence of the indirect effect of exposure to discrimination on anxiety symptoms via co-rumination with friends and the direct effect exposure to discrimination to anxiety symptoms, indicating that gendered racism co-rumination with friends partially mediated the association between exposure to discrimination and anxiety symptoms. Moderation analyses found no evidence that pride and empowerment socialization, oppression awareness socialization, friendship intimacy or friendship support moderated the association between to gendered racial discrimination and anxiety. |
|  |  |  |  |  | Substance use as coping |  |  |  |  |  | **Correlation coefficient = 0.26, p < 0.001** |  |
|  |  |  |  |  | Co-rumination |  |  |  |  |  | **Correlation coefficient = 0.17, p < 0.001** |  |
|  |  |  |  |  | Friendship intimacy |  |  |  |  |  | Correlation coefficient = 0.06 |  |
|  |  |  |  |  | Friendship support |  |  |  |  |  | Correlation coefficient = 0.09 |  |
|  |  |  |  |  | Pride and empowerment socialization |  |  |  |  |  | Correlation coefficient = 0.04 |  |
|  |  |  |  |  | Oppression awareness socialization |  |  |  |  |  | **Correlation coefficient = 0.12, p<0.05** |  |
|  |  |  |  |  | Identity centrality |  |  |  |  |  | Correlation coefficient = 0.07 |  |
|  |  |  |  |  | Identity exploration |  |  |  |  |  | Correlation coefficient = 0.09 |  |
|  |  |  |  |  | Identity commitment |  |  |  |  |  | Correlation coefficient = 0.08 |  |
|  |  |  |  |  | Education level |  |  |  |  |  | Correlation coefficient = -0.06 |  |
|  |  |  |  |  | Financial insecurity |  |  |  |  |  | **Correlation coefficient = 0.23, p < 0.001** |  |
|  |  |  |  |  | Self-reported social status |  |  |  |  |  | **Correlation coefficient = -0.22, p < 0.001** |  |
|  |  |  |  |  | Time online discussing racism |  |  |  |  |  | Correlation coefficient = -0.03 |  |
|  |  |  |  |  | Time online empowerment |  |  |  |  |  | Correlation coefficient = 0.04 |  |
|  |  |  |  |  | Time online oppression awareness |  |  |  |  |  | Correlation coefficient = 0.03 |  |
|  |  |  |  |  | Time online co-rumination |  |  |  |  |  | Correlation coefficient = 0.01 |  |
|  |  |  |  |  | Indirect effect: discrimination to anxiety via co-rumination with friends |  |  |  |  |  | **Standardised regression coefficient = 0.043, 95% CI: 0.001 to 0.091, no p-values reported** |  |
|  |  |  |  |  | Direct effect: discrimination to anxiety symptoms |  |  |  |  |  | **Standardised regression coefficient = 0.16, 95% CI: 0.036 to 0.25, no p-values reported** |  |
|  |  |  |  |  | Discrimination x Pride and Empowerment |  |  |  |  |  | Standardised regression coefficient= -0.03, 95% CI: -0.32, 0.27 |  |
|  |  |  |  |  | Discrimination x Oppression Awareness |  |  |  |  |  | Standardised regression coefficient= -0.05, 95% CI: -0.23, 0.35 |  |
|  |  |  |  |  | Discrimination x Friendship Intimacy |  |  |  |  |  | Standardised regression coefficient= -0.05, 95% CI: -0.30, 0.22 |  |
|  |  |  |  |  | Discrimination x Friendship support |  |  |  |  |  | Standardised regression coefficient= -0.08, 95% CI: -0.34, 0.22 |  |
| Thornhill et al. (2021) (78) USA | 21 (18-25), 51  Latinx emerging adults | Cross-sectional | 200 | To analyse the extent to which ethnic identity components and gender moderate the associations between accusations of assimilation and anxiety. | Ethnic identity exploration x gender x intracultural accusations of assimilation | Generalised Anxiety disorder symptoms | | Generalized Anxiety Disorder scale (GAD-7) | Standardised regression coefficient = −0.18, p > 0.05 | NR | NR | There was no evidence of interactions between ethnic identity exploration/commitment, gender, and intracultural accusations of assimilation ethnic identity in predicting anxiety. |
|  |  |  |  |  | Ethnic identity commitment x gender x intracultural accusations of assimilation |  |  |  | Standardised regression coefficient = −0.05, p > 0.05 | NR | NR |  |
| van Beusekom et al. (2015) (79) Netherlands | 16.6 (15-18), 51.9  Secondary school | Cross-sectional | 1121 | To assess, separately for boys and girls, the moderating effects of mother/father acceptance in the relationship of same-sex attraction and gender nonconformity with psychological distress and social anxiety. | SSA | Social anxiety symptoms | | Social Interaction Anxiety Scale (SIAS) | No formal tests for sex/gender difference | **Correlation coefficient = 0.29, p<0.01** | **Correlation coefficient = 0.14, p<0.01** | In the ‘girls’ subgroup, mother acceptance moderated the association between same-sex attraction with social anxiety; mother acceptance reduced the association between same-sex attraction with social anxiety.  In the ‘boys’ subgroup, father acceptance moderated the association between gender nonconformity and social anxiety; Father acceptance reduced the association between gender nonconformity and social anxiety. |
|  |  |  |  |  | GNC |  |  |  |  | **Correlation coefficient = 0.31, p<0.01** | **Correlation coefficient = 0.25, p<0.01** |  |
|  |  |  |  |  | Mother acceptance |  |  |  |  | **Correlation coefficient = -0.27, p<0.01** | **Correlation coefficient = -0.30, p<0.01** |  |
|  |  |  |  |  | Father acceptance |  |  |  |  | **Correlation coefficient = -0.29, p<0.01** | **Correlation coefficient = -0.23, p<0.01** |  |
|  |  |  |  |  | SSA X mother acceptance |  |  |  |  | Interaction:  Standardised regression coefficient = 0.00, Unstandardised regression coefficient = 0.00, p=0.989 | **Interaction:**  **Standardised regression coefficient = -0.15, Unstandardised regression coefficient = -0.13, p=0.003**  **Low level of mother acceptance:**  **Regression coefficient = 0.14, p=0.003**  Mean level of mother acceptance:  Regression coefficient = 0.03, p=0.367  High level of mother acceptance:  Regression coefficient = -0.07, p=0.200 |  |
|  |  |  |  |  | SSA X father acceptance |  |  |  |  | **Interaction**:  **Standardised regression coefficient = 0.13, Unstandardised regression coefficient = 0.13, p=0.019**  Low levels of father acceptance: Regression coefficient = 0.09, p=0.081  **Mean levels of father acceptance: Regression coefficient = 0.19, p<0.001**  High levels of father acceptance:  Regression coefficient: 0.29, p<0.001  Further examination with ANOVAs: “father acceptance does not influence social anxiety for boys with high levels of SSA” | Interaction:  Standardised regression coefficient = 0.05, Unstandardised regression coefficient = 0.04, p=0.379 |  |
|  |  |  |  |  | GNC X mother acceptance |  |  |  |  | Interaction:  Standardised regression coefficient = -0.01, Unstandardised regression coefficient = -0.01, p=0.825 | Interaction:  Standardised regression coefficient = -0.09, Unstandardised regression coefficient = -0.08, p=0.051 |  |
|  |  |  |  |  | GNC X father acceptance |  |  |  |  | Interaction:  **Standardised regression coefficient = -0.20, Unstandardised regression coefficient = -0.19, p<0.001**  **Low levels of father acceptance: regression coefficient = 0.26, p<0.001**  **Mean levels of father acceptance: regression coefficient = 0.12, p<0.001**  High levels of father acceptance:  Regression coefficient: -0.02, p=0.661 | Interaction:  Standardised regression coefficient = 0.01, Unstandardised regression coefficient = 0.01, p=0.861 |  |
| Vannucci et al. (2017) (80) USA | 20 (18-22), 50.2  Ethnicity:  Non-Hispanic White: 63.3%  African American: 13.5%  Hispanic/Latino: 12.1%, Asian: 7.6%  Other: 3.5% | Cross-sectional | 563 | To examine the impact of time spent using social media on anxiety symptoms and severity in emerging adults. | Social media x gender | Dispositional anxiety symptoms | | Beck Anxiety Inventory-Trait (BAI-T) and Overall Anxiety Severity and Impairment Scale (OASIS) | p=0.35 | NR | NR | There were no  significant interactions in relation to dispositional anxiety symptoms or recent anxiety-related impairment. |
|  |  |  |  |  | Social media x gender | Recent anxiety-related impairment | |  | p=0.90 | NR | NR |  |
| Whitton et al. (2020) (81) USA | NR (18-20), 100 | Cross-sectional | 222 | To examine whether romantic relationship involvement, a well-established protective factor against mental health problems among heterosexual adults, is also protective for sexual and gender minority emerging adults assigned female at birth | Relationship involvement x gender identity | Anxiety symptoms | | Patient-Reported Outcomes Measurement Information System (PROMIS) Anxiety Short Form | NA | NA | **F(1,213) = 6.51, p = 0.01.** | The negative association between relationship involvement and anxiety was moderated by gender identity, sexual identity, and partner gender. Cisgender and lesbian participants who were in a relationship reported lower levels of anxiety than single cisgender and lesbian females respectively. |
|  |  |  |  |  | Relationship involvement x sexual identity |  |  |  |  |  | **F(2,212) = 3.49, p = 0.03** |  |
|  |  |  |  |  | Relationship involvement x Partner gender |  |  |  |  |  | **F(3, 212) = 5.67, p < .01** |  |
|  |  |  |  |  | Relationship involvement x Race |  |  |  |  |  | p>0.05 |  |
| Wijsbroek et al. (2011) (82) Netherlands | Total sample: Baseline: 16.7 (NR), 51  Late adolescent subgroup: 57% female  Ethnicity:  Non-Western ethnic groups: 15.1% | Longitudinal  (Follow-up: 3 x 2-year intervals)  Time 2: Two years after baseline  Time 3: Four years after baseline: | Baseline: 1313  Time 2: 1,293  Time 3: 1,275 | To examine the direction of effects and age and sex differences between adolescents’ perceptions of parental behavioural and psychological control and adolescents’ self-reports of generalized anxiety disorder and separation anxiety disorder symptoms. | Baseline: behavioural control | Baseline: generalized anxiety disorder | | Screen for Child Anxiety Related Emotional Disorders (SCARED) | No formal test for sex/gender difference | **Correlation coefficient = 0.16, p<0.05** | Correlation coefficient = 0.02 | For males, parental psychological and behavioural control were risk factors for anxiety at baseline and two years later in males. However an association between psychological control two years after baseline and anxiety four years after baseline was found in females but not in males. |
|  |  |  |  |  | Baseline: psychological control | Baseline: generalized anxiety disorder | |  |  | **Correlation coefficient = 0.24, p<0.001** | Correlation coefficient = 0.11 |  |
|  |  |  |  |  | Baseline: behavioural control | Time 2: generalized anxiety disorder | |  |  | **Correlation coefficient = 0.21, p<0.001** | Correlation coefficient = 0.02 |  |
|  |  |  |  |  | Time 2: behavioural control | Time 3: generalized anxiety disorder | |  |  | Correlation coefficient = 0.12 | Correlation coefficient = 0.11 |  |
|  |  |  |  |  | Baseline: psychological control | Time 2: generalized anxiety disorder | |  |  | Correlation coefficient = 0.22, p<0.001 | Correlation coefficient = 0.06 |  |
|  |  |  |  |  | Time 2: psychological control | Time 3: generalized anxiety disorder | |  |  | Correlation coefficient = 0.14 | **Correlation coefficient = 0.23, p<0.001** |  |
|  |  |  |  |  | Baseline: behavioural control | Time 2: generalized anxiety disorder | |  |  | **Standardised regression coefficient = 0.13, p<0.05** | Standardised regression coefficient = 0.00 |  |
| Wise et al. (2023) (83) USA | 16.1 (NR), 52 | Cross-sectional | 4874 | The purpose of this study was to determine whether socioeconomic status, race, or gender moderate the benefits of returning to sports during COVID-19. | Returning to sport after Covid restrictions x sex | Anxiety symptoms | | Generalized Anxiety Disorder scale (GAD-7) | Interaction:  Regression coefficient = -0.16, p = 0.76 | NR | NR | No sex/gender differences were identified. The benefits of returning to sports for female athletes were similar to male athletes with respect to anxiety |
| Woodward et al. (2025) (84) USA | 19.1 (NR), 77.7, 80.1% Caucasian, 11.7% African American, 5.2% Hispanic or Latinx, 4.2% Asian American, 4.2% another ethnicity, Sexual Orientation ‘Straight’: 78.8%, University | Cross-sectional | 575 | To examine associations between time spent on multiple popular social media platforms and a variety of mental health-related outcomes in a sample of young adults. To examine findings separately among men and  women to examine whether the influence of specific social  media platforms on mental health varied across genders. | Facebook | Trait anxiety | | State-Trait Anxiety Inventory (STAI) | No formal tests for sex/gender difference | Unstandardised regression coefficient = -0.87 | Unstandardised regression coefficient = 1.04 | Evidence of a positive association between anxiety and use of TikTok in females (p=0.001) but not in males (p-value not reported). YouTube was positively associated with trait anxiety in males (p=0.03) and females (p=0.003). |
|  |  |  |  |  | Instagram |  |  |  |  | Unstandardised regression coefficient = -0.21 | Unstandardised regression coefficient = -0.96 |  |
|  |  |  |  |  | Reddit |  |  |  |  | Unstandardised regression coefficient = 6.31 | Unstandardised regression coefficient = 3.19 |  |
|  |  |  |  |  | Twitter |  |  |  |  | Unstandardised regression coefficient = 0.24 | Unstandardised regression coefficient = -0.64 |  |
|  |  |  |  |  | TikTok |  |  |  |  | Unstandardised regression coefficient = 0.23 | **Unstandardised regression coefficient = 1.23, p<0.01** |  |
|  |  |  |  |  | Snapchat |  |  |  |  | Unstandardised regression coefficient = -0.57 | Unstandardised regression coefficient = -0.59 |  |
|  |  |  |  |  | YouTube |  |  |  |  | **Unstandardised regression coefficient = 1.34, p<0.05** | **Unstandardised regression coefficient = 1.08, p<0.01** |  |
| Zvolensky et al. (2019) (85) USA | 21 (18-25), 83 | Cross-sectional | 401 | To empirically examine worry as an explanatory factor in relation to pain intensity, pain disability, pain-related anxiety, and perceived health among Latinx young adults. | Physical functioning | Worry | | Penn State Worry Questionnaire (PSWQ) | No formal tests for sex/gender difference | Correlation coefficient = -0.04 | Correlation coefficient = -0.06 | Pain intensity was a risk factor for worry in females but not in males.  Subjective social status was a protective factor for worry in females but not in males. |
|  |  |  |  |  | Subjective social status |  |  |  |  | Correlation coefficient = -0.05 | **Correlation coefficient = -0.11, p<0.05** |  |
|  |  |  |  |  | Pain intensity |  |  |  |  | Correlation coefficient = 0.14 | **Correlation coefficient = 0.20, p<0.001** |  |
|  |  |  |  |  | Pain disability |  |  |  |  | Correlation coefficient = 0.07 | Correlation coefficient = 0.10 |  |
|  |  |  |  |  | Perceived health |  |  |  |  | **Correlation coefficient = -0.27, p<0.05** | **Correlation coefficient = -0.27, p<0.001** |  |

**Abbreviations**: Body Mass Index (BMI); Body Mass Index Silhouette Matching Test (BMI-SMT); Confidence interval (CI); Control group (CON); Cyber sexual harassment (CSH); Gay, Lesbian, Bisexual, and Transgender (GLBT); Gender non-conformity (GNC); Gendered racial microaggressions frequency (GRMF); Gendered racial microaggressions stress (GRMS); Heavy episodic group (HED); Hypermobile Ehlers–Danlos syndrome (hEDS); Not reported (NR); Not applicable (NA); Odds ratio (OR); Online racial discrimination (ORD); Problematic Pornography Use (PPU); Same-sex attraction (SSA); same-sex relationship (SSR); Socioeconomic status (SES); Unwanted online sexual solicitation (UOSS); World Health Organization (WHO).

# **Appendix 2. National Institutes of Health Quality Assessment Tool for Observational Cohort and Cross-Sectional Studies**

| **Rating** | **Meaning** | **Criterion developed by Tu et al.** (86)  (The items are listed below) |
| --- | --- | --- |
| Good | The least risk of bias | “Yes” (+) responses to questions 3, 6, 7, 12 and 14; If a “no” response to question 13, a description and implication of the loss-follow-up population are required |
| Fair | Susceptible to some bias |  |
| Poor | Significant risks of bias | “Fatal flaws” indicated by a “no” (-) response to questions 1, 2, 4, 9, or 11 |

Quality Assessment Tool for Observational Cohort and Cross-Sectional Studies, developed by Tu et al. (86)

1. Was the research question or objective in this paper clearly stated?
2. Was the study population clearly specified and defined?
3. Was the participation rate of eligible persons at least 50%?
4. Were all the subjects selected or recruited from the same or similar population (including the same time period)? Were inclusion and exclusion criteria for being in the study prespecified and applied uniformly to all participants?
5. Was a sample size justification, power description, or variance and effect estimates provided?
6. For the analyses in this paper, were the exposure(s) of interest measured prior to the outcome(s) being measured?
7. Was the timeframe sufficient so that one could reasonably expect to see an association between exposure and outcome if it existed?
8. For exposures that can vary in amount or level, did the study examine different levels of the exposure as related to the outcome?
9. Were the exposure measures clearly defined, valid, reliable, and implemented consistently across all study participants?
10. Was the exposure(s) assessed more than once over time?
11. Were the outcome measures clearly defined, valid, reliable, and implemented consistently across all study participants?
12. Were the outcome assessors blinded to the exposure status of participants?
13. Was loss to follow-up after baseline 20% or less?
14. Were key potential confounding variables measured and adjusted statistically for their impact on the relationship between exposure(s) and outcome(s)

# **Appendix 3. Quality assessment of included studies**

| **First author (Year)** | **1** | **2** | **3** | **4** | **5** | **6** | **7** | **8** | **9** | **10** | **11** | **12** | **13** | **14** | **Rating** |
| --- | --- | --- | --- | --- | --- | --- | --- | --- | --- | --- | --- | --- | --- | --- | --- |
| Altin et al. (2024) (1) | + | + | NR | + | + | - | - | + | + | - | + | - | NR | - | Fair |
| Apsley et al. (2020) (2) | + | + | NR | + | - | + | + | + | + | + | + | - | NR | + | Fair |
| Araia et al. (2020) (3) | + | + | - | + | - | - | - | + | + | - | + | - | NR | - | Fair |
| Arsandaux et al. (2023) (4) | + | + | - | + | - | - | - | + | + | - | + | - | NR | + | Fair |
| Atkinson et al. (2019) (5) | + | + | NR | + | - | - | - | + | + | - | + | - | NR | + | Fair |
| Barcaccia et al. (2018) (6) | + | + | NR | + | - | - | - | + | - | + | + | - | NR | - | Fair/Poor  Constructed questionnaire |
| Barry et al. (2015) (7) | + | + | NR | + | - | - | - | + | + | - | + | - | NR | - | Fair |
| Bauermeister et al. (2017) (8) | + | + | NR | + | - | - | - | + | + | - | + | - | NR | + | Fair |
| Bauermeister et al. (2010) (9) | + | + | NR | + | - | + | + | + | + | + | + | - | + | + | Fair |
| Bekman et al. (2013) (10) | + | + | NR | + | - | + | - | + | + | + | + | - | + | + | Fair |
| Benedetto et al. (2018) (11) | + | + | NR | + | - | - | - | + | + | - | + | - | NR | - | Fair |
| Berenz et al. (2019) (12) | + | + | - | + | - | - | - | + | + | - | + | - | NR | + | Fair |
| Bernard et al. (2017) (13) | + | + | NR | + | - | + | + | + | + | + | + | - | + | + | Good/Fair |
| Bernusky et al. (2021) (14) | + | + | NR | + | + | - | - | + | + | - | + | - | NR | - | Fair |
| Bieniak et al. (2022) (15) | + | + | NR | + | + | - | - | + | + | - | + | - | NR | - | Fair |
| Bilali et al. (2025) (16) | + | + | + | + | + | - | - | + | + | - | + | - | NR | + | Fair |
| Bluth et al. (2017) (17) | + | + | + | + | + | - | - | + | + | - | + | - | NR | + | Fair |
| Brolin et al. (2024) (18) | + | + | NR | + | - | + | + | + | + | - | + | - | - | + | Fair |
| Buckner et al. (2024) (19) | + | + | + | + | - | - | - | + | + | - | + | - | NR | - | Fair |
| Burke et al. (2023) (20) | + | + | NR | + | - | - | - | + | + | - | + | - | NR | + | Fair |
| Cano et al. (2021) (21) | + | + | NR | + | - | - | - | + | + | - | + | - | NR | + | Fair |
| Carcedo et al. (2020) (22) | + | + | NR | + | - | - | - | + | + | - | + | - | NR | - | Fair |
| Carlberg et al. (2024) (23) | + | + | - | + | - | - | - | + | - | - | + | - | NR | - | Fair/Poor  Constructed questionnaire |
| Carollo et al. (2024) (24) | + | + | NR | + | + | - | - | + | + | - | + | - | NR | - | Fair |
| Chen et al. (2021) (25) | + | + | NR | + | - | - | - | + | + | - | + | - | NR | + | Fair |
| Christiansen et al. (2021) (26) | + | + | NR | + | - | - | - | + | + | - | - | - | NR | + | Poor  Constructed anxiety questionnaire |
| Davila et al. (2020) (27) | + | + | NR | + | - | - | - | + | + | - | + | - | NR | - | Fair |
| Davis et al. (2022) (28) | + | + | NR | + | - | + | + | + | + | + | + | - | + | + | Good/Fair |
| De France et al. (2022) (29) | + | + | NR | + | - | + | + | + | + | + | + | - | + | + | Good/Fair |
| Di Blasi et al. (2015) (30) | + | + | NR | + | - | - | - | + | + | - | + | - | NR | + | Fair |
| Duncan et al. (2019) (31) | + | + | NR | + | - | - | - | + | + | - | + | - | NR | - | Fair |
| Durham et al. (2025) (32) | + | + | NR | + | - | - | - | + | + | - | + | - | NR | + | Fair |
| El-Sheikh et al. (2022) (33) | + | + | NR | + | - | - | - | + | + | - | + | - | NR | + | Fair |
| Ferro et al. (2016) (34) | + | + | NR | + | - | - | - | + | + | - | + | - | NR | + | Fair |
| Fitzsimmons et al. 2012 (35) | + | + | NR | + | - | + | + | + | + | + | + | - | NR | + | Good/Fair |
| Fontaine et al. 2019 (36) | + | + | + | + | - | + | + | + | + | + | + | - | + | + | Good |
| Fortier et al. (2025) (37) | + | + | + | + | - | + | + | + | + | + | - | - | + | + | Fair/Poor  Constructed questionnaire |
| Gassó et al. (2020) (38) | + | + | NR | + | - | - | - | + | + | - | + | - | NR | - | Fair |
| Giannotta et al. (2024) (39) | + | + | - | + | - | + | + | + | + | + | + | - | - | + | Fair |
| Gillespie et al. (2023) (40) | + | + | NR | + | - | + | + | + | + | + | + | - | - | - | Good/Fair |
| González-Díez et al. (2017) (41) | + | + | NR | + | - | + | + | + | + | + | + | - | - | - | Good/Fair |
| Grapin et al. (2024) (42) | + | + | NR | + | - | - | - | + | + | - | + | - | NR | + | Fair |
| Hawes et al. (2020) (43) | + | + | NR | + | - | - | - | + | + | - | + | - | NR | + | Fair |
| Hellemans et al. (2019) (44) | + | + | NR | + | - | - | - | + | + | - | + | - | NR | - | Fair |
| Herring et al. (2021) (45) | + | + | NR | + | - | - | - | + | + | - | + | - | NR | - | Fair |
| Johannessen et al. (2017) (46) | + | + | + | + | - | - | - | + | - | - | + | - | NR | + | Poor  Constructed dichotomous alcohol variables |
| Kaltschik et al. (2022) (47) | + | + | NR | + | - | - | - | + | + | - | + | - | NR | - | Fair |
| Kim et al. (2021) (48) | + | + | NR | + | - | - | - | + | + | - | + | - | NR | - | Fair |
| Kornilaki et al. (2022) (49) | + | + | NR | + | - | - | - | + | - | - | + | - | NR | - | Fair/Poor  Constructed questionnaire |
| Kouros et al. (2017) (50) | + | + | NR | + | - | - | - | + | + | - | + | - | NR | + | Fair |
| Laftman et al. (2024) (51) | + | + | NR | + | - | + | + | - | - | + | + | - | NR | + | Fair/Poor  Crude dichotomous bullying  variables |
| Larsen et al. (2025) (52) | + | + | + | + | + | + | + | + | + | + | + | - | + | + | Good/Fair |
| Leadbeater et al. (2014) (53) | + | + | NR | + | - | + | + | + | + | + | + | - | + | + | Fair |
| Lee et al. (2022) (54) | + | + | NR | + | - | - | - | + | + | - | + | - | NR | + | Fair |
| Lin et al. (2020) (55) | + | + | NR | + | - | - | - | + | + | - | + | - | NR | + | Fair |
| Maciel et al. (2020) (56) | + | + | NR | + | - | - | - | + | + | - | + | - | NR | - | Fair |
| Mar et al. (2024) (57) | + | + | + | + | - | - | - | - | + | + | + | - | NR | + | Fair  dichotomous SES variables: “low SES”, “medium-to-high SES” |
| Marshal et al. (2011) (58) | + | + | + | + | - | - | - | - | + | - | + | - | NR | + | Fair  dichotomous sexual orientation variable |
| Mayorga et al. (2018) (59) | + | + | + | + | - | - | - | + | + | - | + | - | NR | - | Fair |
| McKinney et al. (2011) (60) | + | + | NR | + | + | - | - | + | + | - | + | - | NR | - | Fair |
| Minhas et al. (2013) (61) | + | + | + | + | - | + | + | + | + | - | + | - | NR | - | Fair |
| Neblett et al. (2016) (62) | + | + | NR | + | - | - | - | + | + | - | + | - | NR | + | Fair |
| Pachankis et al. (2012) (63) | + | + | NR | + | - | - | - | + | + | - | + | - | NR | - | Fair |
| Perkins et al. (2022) (64) | + | + | NR | + | - | - | - | + | - | - | + | - | NR | + | Fair/Poor  Constricted racial discrimination variable |
| Poteat et al. (2014) (65) | + | + | NR | + | - | + | + | + | + | + | + | - | + | + | Good/Fair |
| Ranta et al. (2013) (66) | + | + | NR | + | - | + | + | - | + | + | + | - | - | + | Fair  Dichotomous bullying variable |
| Reed et al. (2019) (67) | + | + | NR | + | - | - | - | - | - | - | + | - | NR | + | Fair/Poor  Dichotomous and constructed variable CSH |
| Rieselbach et al. (2014) (68) | + | + | + | + | - | + | + | + | + | + | + | - | + | + | Good/Fair |
| Sares-Jaske et al. (2023) (69) | + | + | + | + | - | - | - | + | - | - | + | - | NR | + | Fair/Poor  Constructed bullying variable |
| Schiffrin et al. (2019) (70) | + | + | NR | + | - | - | - | + | + | - | + | - | NR | - | Fair |
| Sebokova et al. (2016) (71) | + | + | NR | + | - | - | - | + | + | - | + | - | NR | - | Fair |
| Shorey et al. (2015) (72) | + | + | + | + | - | - | - | + | + | - | + | - | NR | - | Fair |
| Skogen et al. (2021) (73) | + | + | + | + | - | - | - | + | - | - | + | - | NR | - | Fair/Poor  self-presentation on social media questions generated using an inductive approach based on qualitative interview |
| Skogen et al. (2014) (74) | + | + | + | + | - | - | - | + | + | - | + | - | NR | - | Fair |
| Stearns et al. (2021) (75) | + | + | NR | + | - | - | - | + | + | - | + | - | NR | - | Fair |
| Surprenant et al. (2025) (76) | + | + | + | + | - | - | - | - | - | - | + | - | NR | - | Poor  Constructed questionnaire, dichotomous variables |
| Tao et al. (2025) (77) | + | + | + | + | + | - | - | + | + | - | + | - | NR | + | Fair |
| Thornhill et al. (2021) (78) | + | + | + | + | - | - | - | + | + | - | + | - | NR | + | Fair |
| van Beusekom et al. (2015) (79) | + | + | NR | + | - | - | - | + | + | - | + | - | NR | + | Fair |
| Vannucci et al. (2017) (80) | + | + | NR | + | - | - | - | + | + | - | + | - | NR | - | Fair |
| Whitton et al. (2020) (81) | + | + | + | + | - | - | - | + | + | - | + | - | NR | - | Fair |
| Wijsbroek et al. (2011) (82) | + | + | + | + | - | - | - | + | + | - | + | + | + | - | Fair |
| Wise et al. (2023) (83) | + | + | NR | + | - | - | - | - | + | - | + | - | NR | + | Fair/Poor  Dichotomous SES variables |
| Woodward et al. (2025) (84) | + | + | + | + | - | - | - | + | - | - | + | - | NR | + | Fair/Poor  Constructed questionnaire |
| Zvolensky et al. (2019) (85) | + | + | NR | + | - | - | - | + | + | - | + | - | NR | - | Fair |

# **Appendix 4. Certainty of evidence GRADE assessment**

| **GRADE domain*** | **Judgement** | **Concerns about certainty domains** | **Certainty in the evidence** |
| --- | --- | --- | --- |
| Methodological limitations of the studies | Nine studies justified their sample size by conducting a power calculation (1, 14-17, 24, 52, 60, 77). 62 studies were cross-sectional studies and therefore did not meet the following criteria: exposures being measured prior to the outcome; a sufficient timeframe; exposures being assessed more than once over time, and the loss to follow-up after baseline being 20% or less. Furthermore, only one study reported that outcome assessors were blinded to the exposure status of participants (82). Six studies did not meet the criteria for examining different levels of the exposure as related to the outcome and instead had dichotomous exposure variables (51, 57, 58, 66, 67, 76, 83). All exposure measures were clearly defined, but 11 studies constructed their own exposure (6, 23, 37, 46, 49, 51, 67, 69, 73, 76, 87), and outcome (26) variables which were not validated. Therefore, we judged the studies included in this review to have serious methodological limitations. | Serious | Very low  ⊕OOO  (due to serious risk of bias, inconsistency and imprecision) |
| Indirectness | The studies all provide direct evidence to the research question of this review, however the approach to sex/gender analysis varied in this review from exploring the moderating role of sex/gender to reporting sex/gender stratified associations between anxiety and the risk or protective factors. Of the studies reporting sex/gender stratified results, 43 studies had no formal tests for sex/gender differences between results. | Not serious, borderline |  |
| Imprecision | The total number of participants involved in each study ranged from 34 (15) to 609,381 (57). There was also a sex/gender imbalance, as 32 studies had more than 60% of one gender (3, 4, 12-16, 20, 23, 24, 27, 31, 32, 38, 42, 44, 45, 47, 49, 50, 56, 59, 60, 62, 64, 65, 70-72, 75, 85, 87). For two of the smaller studies (50, 62) the overrepresentation of females in the sample, introduces a risk of studies detecting an association between anxiety and the risk factor in the female subgroup, but not in the male subgroup, due to its smaller sample size and therefore limited precision. This is only an issue for studies with small sample sizes, for example Kouros et al.’s small sample size of 116 and its 83.1% female majority limits its precision, and the sex/gender difference reported in this study could be due to the analysis having insufficient power to detect an association in males (50). | Not serious, borderline |  |
| Inconsistency | This review has conflicting findings, indicating inconsistency. | Serious |  |
| Publication bias | Publication bias is not suspected of the included studies due to their conflicting findings. | Not suspected |  |

*Following Murad et al.’s (88) guidance on using GRADE to rate the certainty of evidence when narrative synthesis is conducted instead of meta-analysis.

# **Appendix 5. Individual-level: Summary of results for sex/gender-specific modifiable factors**

|  |  | **Males/men/boys** | **Females/women/girls** | **No sex/gender difference** |
| --- | --- | --- | --- | --- |
| **Individual negative life events and family adversity** | **Negative life events x ‘Difficulty with goal-directed behaviour’ on the emotional regulation scale** | Sex/gender moderation (interaction: p=0.025). Risk factor for males in upper and middle terciles (those who have more difficulty engaging in goal-directed behaviour (p-values not reported) (32). |  |  |
|  | **Sexual harassment x age** | Sex/gender moderation (interaction p = 0.006). Risk factor for males aged 18 (p=0.002) and 20 years (p=0.001) (31). | Sex/gender moderation (interaction p = 0.006).  Risk factor for females aged 18 (p=0.013) and 16 years (p=0.001) (31). |  |
|  | **Unwanted online sexual solicitation** | Sex/gender moderation (interaction: p<0.001). Greater risk factor for anxiety in males (no p-values reported) (23). |  |  |
|  | **Child sexual abuse, where victims had family ties with the perpetrator** |  | Risk factor (p<0.05) (56) |  |
|  | **Sexual abuse** |  |  | No association in males or females (p values not reported) (27) |
|  | **Online sexual harassment** |  | Female-only: risk factor (p<0.001) (67) |  |
|  |  |  | Risk factor (p<0.01) (38) |  |
|  | **Childhood maltreatment** | Risk factor (p<0.01) (27) |  |  |
|  | **Parents' emotional abuse** |  |  | No association in males (p=0.07) and females (p=0.08) (41) |
|  | **Family history of alcohol use disorder** |  | Risk factor p<0.05 (12) |  |
|  | **Family history of anxiety or depression** |  | Risk factor p=0.001 (12) |  |
|  | **Parent problem drinking** |  |  | No sex moderation (interaction: p=0.296) (18) |
|  | **Perceived maternal and paternal anxiety** |  |  | Risk factors in both males and females (all p<0.01) (75) |
|  | **Number of lifetime traumatic events** |  | Risk factor p=0.003 (12) |  |
| **Self-esteem, identity and related factors** | **Self-compassion** | Male-only study: risk factor (p<0.001) (7) | Sex/gender moderation (interaction p = 0.008):  Risk factor (p = 0.013) (17) |  |
|  | **Self-image subscale: Positive perceptions of family relationships** |  | Risk factor (p=0.007) (30) |  |
|  | **Subscales of self-image: External Mastery** |  | Protective factor (p=0.003) (30) |  |
|  | **Subscales of self-image: Emotional Tone** | Risk factor (p=0.001) (30) |  |  |
|  | **Self-esteem** | Male-only study: risk factor (p<0.001) (7). |  | Risk factors in both males and females (all p<0.0001) (4) |
|  |  |  |  | Risk factor in both males and females (all p<0.01) (60) |
|  | **Self-identity disturbance** |  |  | Risk factor in both males and females (all p<0.0001) (25) |
|  | **Self-** **consciousness** |  |  | No association in males or females (p-values not reported) (71) |
|  | **Centrality** |  |  | No sex/gender moderation (p-values not reported) (64) |
|  | **Identity centrality, commitment or exploration** |  | Female-only study:  No associations (p-values not reported) (77) |  |
|  | **Imposter phenomenon** |  |  | No sex/gender moderation (p=0.21) (13) |
|  | **Self-worth** |  |  | Risk factor in both males and females (all p<0.0001) (31) |
| **Health behaviours** | **Early onset of alcohol consumption** |  | Sex/gender moderation (interaction p = 0.01): Stronger risk factor in females (p-value not reported) (46) |  |
|  |  |  | Risk factor (p = 0.03) (12) |  |
|  | **Excessive Alcohol consumption/alcohol problems/Alcohol abuse or dependence symptoms** | Sex/gender difference (p<0.05): Stronger risk factor in males (5) | Sex/gender moderation (interaction p<0.001):  Risk factor in females (p-value not reported) (74) | Risk factors in males and females (both p<0.05) (27) |
|  |  |  | Sex/gender difference (p<0.05): Stronger risk factor in females (5) |  |
|  | **Alcohol use** |  |  | No association in males or females (no p-values reported)(72) |
|  |  |  |  | No sex/gender moderation (p=0.952) (10) |
|  | **Drinking to cope/conform/to be social/to enhance enjoyment** |  | Female-only study:  Risk factors (p<0.01) (19) |  |
|  | **Alcohol-related problems** |  | Female-only study:  Risk factor (p<0.01) (19) |  |
|  | **Substance use** |  | Sex/gender difference (p<0.05):  Protective factor (68) |  |
|  | **Substance use as coping** |  | Female-only study:  Risk factor (p<0.001) (77) |  |
|  | **Problematic cannabis use** |  | Sex/gender moderation (interaction p<0.01):  Stronger risk factor in females (p<0.001) (44). |  |
|  | **Illicit drug use** | Sex/gender moderation (interaction p=0.021): Risk factors in males (p-value not reported) (74). |  |  |
|  | **Drug abuse or dependence symptoms** |  |  | Risk factors in males and females (both p<0.01) (27) |
|  | **Cannabis use** |  |  | Risk factors in males and females (both p<0.05) (28) |
|  |  |  |  | No evidence of sex/gender moderation (p>0.05) (14) |
|  | **Drug Use Disorders** |  |  | No association in males or females (no p-values reported) (72) |
|  | **Physical activity** | Risk factor (p<0.001) (45) |  | Risk factors in males and females (both p<0.01) (47) |
|  |  |  |  | No association in males or females (37) |
|  | **Vigorous physical activity** |  |  | No evidence of sex/gender moderation (interaction: non-significant p-value, not reported) (39). |
|  | **Returning to sport during Covid** |  |  | No evidence of sex/gender moderation (p = 0.76) (83) |
|  | **Sleep problems** |  |  | No evidence of sex/gender moderation (interaction: non-significant p-value, not reported) (39). |
| **Physical health** | **Chronic health conditions** |  |  | No evidence of sex/gender moderation (34) |
|  | **Physical functioning** |  |  | No association in males or females (85) |
|  | **Pain intensity** |  | Risk factor (p<0.001) (85) |  |
|  | **Pain disability** |  |  | No association in males or females (85) |
|  | **Perceived health** |  |  | Protective factor in males (p<0.05) and females (p<0.001) (85) |
|  | **Functional disability** |  |  | No sex/gender difference (p=0.82) (15) |
|  | **Disability or health problem** |  |  | Risk factor in males (p<0.05) and females (p<0.001) (76) |
|  | **Diabetes distress** |  |  | Risk factor in males and females (both p<0.0001) (3) |
|  | **Diabetes resilience** |  |  | Protective factor in males and females (both p<0.0001) (3) |
|  | **Body image** |  |  | Risk factors in males and females (both p<0.0001)(3) |
|  | **Body surveillance and body dissatisfaction** |  | Female only study: Risk factor in Caucasian (p<0.001) and African American (p<0.001) young women (35). |  |
|  | **BMI** |  | Female only study: Risk factor in African American young women (p<0.05) (35). |  |
| **Socioeconomic variables** | **SES x racial discrimination** | Sex/gender moderation (interaction p=0.028): young men from lower SES backgrounds (p<0.001) (62). | Sex/gender moderation (interaction p=0.028): young women from higher SES backgrounds (p=0.002) (62). |  |
|  | **Subjective social status** |  | Protective factor (p<0.05) (85) |  |
|  | **SES** |  | Greater risk factor in females (medium-to-high SES: p<0.01 and low SES: p<0.05) (57) | No association in males or females (36) |
|  |  |  | Female-only study:  Protective factor (p<0.01) (77) |  |
|  | **Low parent education** |  | Female-only study:  Risk factor (p<0.01) (58). |  |
|  | **Parental education** |  |  | No association in males or females (51) |
| **Education** |  | Male only study: Risk factor (p<0.05) (8). | Female-only study:  No associations (no p-value reported) (77) | No association in males or females (72) |
| **Income** | **Familial/household income** |  |  | No association in males or females (20) |
|  | **Very poor/poor perception of income** |  |  | Risk factor in males (p<0.001) and females (p<0.01) (76) |
|  | **Personal financial strain** |  |  | Risk factor in males and females (both p<0.01) (59) |
|  | **Financial insecurity** |  | Female-only study:  Risk factor (p<0.001) (77) |  |
| **Family type and parental country of origin** | **Living with one parent** | Risk factor (p<0.05) (51) |  |  |
|  | **Living in an “other” family type (not with parents or in a shared residence)** |  | Risk factor (p<0.05) (51) |  |
|  | **Having two parents born outside the country of residence** | Risk factor (p<0.05) (51) |  |  |
| **Parental unemployment** |  | Risk factor (p<0.05) (66) |  |  |
| **Social media and screen time** | **Daily social media use** |  | Sex/gender difference (p = 0.04): Risk factor in females (p<0.01) (43). | No sex/gender moderation (80) |
|  | **Smartphone usage** |  | Risk factor (p < 0.01) (47) |  |
|  | **Screen time** |  |  | No association in males or females (37) |
|  | **Hours media watched; Idolization of characters; Desire to be similar** |  |  | No association in males or females (6) |
|  | **Discrepancy from ideals seen in media** |  |  | Risk factor in males (p<0.05) and females (p<0.01) (6) |
|  | **Social media intensity** |  |  | “No gender differences” (43) |
|  | **Social media time spent** |  | Sex/gender difference (p = 0.04): Risk factor in females (p<0.01) (43). |  |
|  | **General social media preoccupation** |  |  | “No gender differences” (43) |
|  | **Focus on self-presentation on social media** |  |  | Risk factor in males and females (both p<0.001) (73) |
|  | **Social media use** |  |  | No sex/gender moderation (Dispositional anxiety symptoms: p=0.35; Recent anxiety-related impairment: p=0.90) (80). |
|  | **TikTok addiction scale: TikTok’s capacity to enhance one’s emotional state** |  |  | Risk factor for anxiety in boys (p=0.021) and girls (p=0.031) (16). |
|  | **TikTok addiction scale: TikTok interferes with daily activities** | Risk factor in males (p<0.006) but not females (p=0.081) (16). |  |  |
|  | **TikTok use** |  | Risk factor (p=0.001) (87) |  |
|  | **YouTube use** |  |  | Risk factor in males (p=0.03) and females (p=0.003) (87) |
|  | **Cyber-pornography addiction** |  |  | Risk factor in in males (p < 0.05) and females (p < 0.001) (1). |
| **Religion** | **Religious traits assessment: Social Support** |  | Protective factor (p<0.01) (75) |  |
|  | **Religious traits assessment: conservatism** |  | Protective factor (p<0.01) (75) |  |
|  | **Religiosity x maternal anxiety** |  | Sex/gender difference between interactions (p<0.001)  Female interaction: (p<0.05), religiosity exacerbated the positive association between maternal and daughters’ anxiety (p=0.001) (75). |  |

# **Appendix 6. Interpersonal-level: Summary of results for sex/gender-specific modifiable factors**

|  |  | **Males/men/boys** | **Females/women/girls** | **No sex/gender difference** |
| --- | --- | --- | --- | --- |
| **Social support** | **General social support** |  |  | Protective factor in males and females (p≤0.01) (9) |
|  |  |  |  | No evidence of sex/gender difference (p=0.11) (15) |
|  | **Friendship intimacy** |  | Female-only study:  No associations (no p-values reported) (77) |  |
|  | **Friendship support** |  |  |  |
| **Bullying and victimisation** | **General victimisation** |  |  | Risk factor for males and females (all p<0.001) (65) |
|  | **Bullying** |  |  | No evidence of sex/gender difference (p=0.393) (51) |
|  |  | Cisgender and transmasculine greater positive association (p-values not reported, 95% confidence intervals did not overlap with other gender groups, indicating a statistically significant gender difference) (69). |  |  |
|  | **Relational victimization** |  | **Sex/gender moderation** (interaction p<0.001): Risk factor in females (p<0.01) (53). |  |
|  |  |  | Risk factor (p<0.05) (66) |  |
|  | **Physical/direct victimization** | Risk factor (p<0.05) (66) |  | No association (p-values not reported) (53) |
|  | **Peer victimisation** |  |  | Risk factor in males (p=0.05) and females (p=0.07) (41) |
|  | **Appearance-related victimization (general and online)** |  |  | Risk factor in males and females (both p<0.001) (31) |
|  | **Social media victimization** |  |  | No association in males and females (both p<0.001) (31) |
| **Romantic relationships and sexual behaviours** | **Relationship involvement** |  | Female-only study: Risk factor (p=0.01) (81) |  |
|  | **Same-sex relationship involvement** |  |  | No association (p values not reported) (9) |
|  | **Relationship status** |  |  | No evidence of sex/gender moderation (p-values not reported) (22) |
| **Autonomy support** | **Paternal autonomy support** | Protective factor (p<0.01) (11) |  |  |
|  | **Maternal autonomy support** |  | Protective factor (p<0.01) (11) |  |
|  | **Autonomy support** | Sex/gender moderation (interaction p=0.019): Protective factor (p=0.002) (50). |  |  |
| **Positive parental relationships** | **Paternal emotional availability** | Protective factor (p<0.05) (11) |  |  |
|  | **Maternal emotional availability** |  |  | Protective factor in males and females (both p<0.01) (11) |
|  | **Mother acceptance** |  |  | Protective factor in males and females (both p<0.01)(79) |
|  | **Father acceptance** |  |  | Protective factor in males and females (both p<0.01)(79) |
|  | **Paternal caring** |  |  | Protective factor in males and females (both p<0.01)(60) |
|  | **Maternal caring** |  | Protective factor (p<0.01) (60) |  |
|  | **Parental care** | Protective factor against anxiety in males (p=0.002) but not females (p=0.153).  Protective factor against worry in males (p<0.001) but not females (p=0.044) (24). |  |  |
|  | **Maternal connection** | Protective factor (p=0.001) (2) |  |  |
|  | **Positive family environment** |  |  | No evidence sex/gender moderation (48) |
| **Parenting styles** | **Maternal authoritative parenting styles** |  | Protective factor (p<0.01) (60) |  |
|  | **Paternal authoritative parenting styles** |  | Protective factor (p<0.01) (60) |  |
|  | **Paternal overprotection** |  |  | Risk factor in males and females (both p<0.01) (60) |
|  | **Maternal overprotection** |  | Risk factor (p < 0.01) (60) |  |
|  | **Parental overprotection** |  | Risk factor for anxiety (p=0.008) (24) |  |
|  |  |  | Protective factor against worry (p<0.001)(24) |  |
|  | **Maternal permissive parenting** |  |  | Risk factor in males females (both p<0.01) (60) |
|  | **Authoritarian parenting styles, non-violent, psychological aggression, corporeal punishment and severe assault discipline strategies** |  |  | Risk factor in males and females (all p<0.01) (60) |
|  | **Maternal helicopter parenting** |  |  | Risk factor in males (p=0.02) and females (p<0.001) (70) |
|  | **Helicopter parenting** |  |  | No evidence sex/gender moderation (50) |
| **Negative parental behaviours** | **Maternal psychological control** |  | Risk factor (p=0.005) (2) |  |
|  | **Parental psychological control** | Risk factor at baseline (p<0.001) and two years after baseline (p<0.05) (82) | Risk factor four years after baseline (p<0.001) (82) |  |
|  | **Parental behavioural control** | Risk factor at baseline (p<0.05,) and two years after baseline (p<0.001) (82) |  |  |
|  | **Family hostility** |  |  | No evidence of sex/gender moderation (48) |
|  | **Intracultural family accusations of assimilation** | Sex/gender moderation  (interaction p< 0.01)**:**  Risk factor (p < 0.001) (78) |  |  |
|  | **Parental disapproval** | Male-only study:  Risk factor (p<0.05) (63) |  |  |

# **Appendix 7. Local community-level: Summary of results for sex/gender-specific modifiable factors**

|  |  | **Males/men/boys** | **Females/women/girls** | **No sex/gender difference** |
| --- | --- | --- | --- | --- |
| **Social isolation and loneliness** | |  |  | No association (71) |
|  |  |  |  | No sex/gender moderation (26) |
|  | |  |  | Risk factor in males and females (both p<0.001) (1) |
| **Residential Greenness x age** | |  |  | No evidence of sex/gender moderation (interaction: non-significant p-value, not reported) (52). |
| **School setting** | **Homework** |  | Risk factor (p<0.001) (76) |  |
| **Community networks, engagement and inclusivity** | **Assimilation (resettled Somalian young people)** | Risk factor (p<0.01) (78) |  |  |
| **Safety, crime and violence** | **Non-violent and violent delinquency** |  |  | No sex/gender difference (36) |

# **Appendix 8. Wider society-level: Summary of results for sex/gender-specific modifiable factors**

|  |  | **Males/men/boys** | **Females/women/girls** | **No sex/gender difference** |
| --- | --- | --- | --- | --- |
| **Experience of discrimination, stigma and prejudice** | **Social media discrimination** | Sex/gender moderation (interaction p=0.01): Risk factor for anxiety in males (p≤0.001) (21) |  |  |
|  | **Internalised sexual stigma** |  | Sex/gender moderation (interaction p<0.05):  Risk factor (p<0.001)(54) |  |
|  | **Discrimination x family hostility** |  | Sex/gender moderation (interaction p<0.01): Family hostility reduces the association between discrimination and anxiety in women only (p<0.01) (48) |  |
|  | **Discrimination x sleeping habits** |  | Sex/gender moderation (interaction p<0.05):  Poor sleeping exacerbated association between discrimination and anxiety in females (33) |  |
|  | **Racial discrimination x SES** | Sex/gender moderation (interaction p = 0.028): Lower SES males (p<0.001) at greater risk of the anxiety associated with racial discrimination (62) | Sex/gender moderation (interaction p = 0.028):  Higher SES Females (p=0.002) at greater risk of anxiety associated with racial discrimination (62) |  |
|  | **Offline discrimination x public regard** | Sex/gender moderation (interaction p<0.01): Black males with higher public regard at greater risk of anxiety associated with offline discrimination (p<0.001) (62) | Sex/gender moderation (interaction p<0.01):  Higher public regard reduced the association offline discrimination and anxiety in black females (p<0.001) (62) |  |
|  | **Online discrimination x public regard** |  | Sex/gender moderation (interaction p<0.05):  Higher public regard reduced the association between online discrimination and anxiety in black females (p= 0.001) (62) |  |
|  | **Online racial discrimination** |  | Gender moderation (interaction: p=0.019): risk factor in women (p=0.014) but not in men (p=0.177) (42) |  |
|  | **Discrimination** | Assimilation mediated the positive association between discrimination and anxiety symptoms in males only (40) |  | Risk factor in males (p<0.05) and females (p<0.01) (40) |
|  | **Perceived weight stigma** |  |  | Risk factor in males (p<0.05) and females (p<0.001)(55) |
|  | **Sexual orientation microaggression** |  |  | No sex/gender difference (p=0.49) (25) |
|  | **Homophobic victimisation** | Sex/gender moderation (interaction p<0.05): Risk factor (p<0.001) (65). |  |  |
|  | **Gendered racial microaggression stress** |  | Female-only study: risk factor of social anxiety (p<0.001) and generalised anxiety (p<0.01) (20) |  |
|  | **Gendered racial discrimination** |  | Female-only study:  Risk factor (p<0.001) (77) |  |
|  | **Oppression awareness socialization** |  | Female-only study:  Risk factor (p<0.05) (77) |  |
|  | **Pride and empowerment socialization** |  | Female-only study:  No associations (no p-values reported) (77) |  |
|  | **Time online discussing racism** |  |  |  |
|  | **Time online empowerment** |  |  |  |
|  | **Time online oppression awareness** |  |  |  |
|  | **Indirect effect: Gendered racial discrimination to anxiety via co-rumination with friends** |  | Female-only study:  Risk factor (no p-values reported) (77) |  |
|  | **Direct effect: Gendered racial discrimination to anxiety** |  |  |  |
|  | **Gender policing** | Male-only study: Risk factor (p<0.001) (8) |  |  |
|  | **Disciplinary actions related to gender policing** | Male-only study: Risk factor (p<0.005) (8) |  |  |
|  | **Sexual orientation concealment** | Male-only study: Risk factor (p<0.01) (63) |  |  |
|  | **Public self-consciousness** | Male-only study: Risk factor (p<0.01) (63) |  |  |
|  | **Racial discrimination frequency** |  |  | No evidence of sex/gender moderation (p=0.77) (13) |
|  | **Racial discrimination bother** |  |  | No evidence of sex/gender moderation (p=0.21) (13) |
|  | **Past-year experiences of sexism** |  | Female-only study:  Risk factor (p < 0.01) (19) |  |
| **Covid-19** | **Covid-19** |  | Sex/gender moderation (interaction p=0.00988):  Risk factor in females (p<0.001) (61). |  |
|  | **Life disruption due to Covid-19** |  | Sex/gender moderation (interaction p=0.010): Stronger risk factor for females (p-values not reported) (49). |  |
|  | **Fear of the Covid-19** | Sex/gender differences (p = 0.001): Stronger risk factor for males (p-values not reported) (29). |  |  |

**References**

1. Altin M, De Leo D, Tribbia N, Ronconi L, Cipolletta S. Problematic Pornography Use, Mental Health, and Suicidality among Young Adults. International journal of environmental research and public health. 2024;21(9).

2. Apsley HB, Padilla-Walker LM. Longitudinal links between parents' mental health, parenting, and adolescents' mental health: Moderation by adolescent sex. J Fam Psychol. 2020;34(7):886-92.

3. Araia E, King RM, Pouwer F, Speight J, Hendrieckx C. Psychological correlates of disordered eating in youth with type 1 diabetes: Results from diabetes MILES Youth-Australia. Pediatr Diabetes. 2020;21(4):664-72.

4. Arsandaux J, Boujut E, Salamon R, Tzourio C, Galéra C. Self-esteem in male and female college students: Does childhood/adolescence background matter more than young-adulthood conditions? Personality and Individual Differences. 2023;206.

5. Atkinson EA, Finn PR. Sex Differences in Trait Anxiety's Association with Alcohol Problems in Emerging Adults: The Influence of Symptoms of Depression and Borderline Personality. J Subst Use. 2019;24(3):323-8.

6. Barcaccia B, Balestrini V, Saliani AM, Baiocco R, Mancini F, Schneider BH. Dysfunctional eating behaviors, anxiety, and depression in Italian boys and girls: the role of mass media. Braz J Psychiatry. 2018;40(1):72-7.

7. Barry CT, Loflin DC, Doucette H. Adolescent self-compassion: Associations with narcissism, self-esteem, aggression, and internalizing symptoms in at-risk males. Personality and Individual Differences. 2015;77:118-23.

8. Bauermeister JA, Connochie D, Jadwin-Cakmak L, Meanley S. Gender Policing During Childhood and the Psychological Well-Being of Young Adult Sexual Minority Men in the United States. American journal of men's health. 2017;11(3):693-701.

9. Bauermeister JA, Johns MM, Sandfort TG, Eisenberg A, Grossman AH, D'Augelli AR. Relationship trajectories and psychological well-being among sexual minority youth. J Youth Adolesc. 2010;39(10):1148-63.

10. Bekman NM, Winward JL, Lau LL, Wagner CC, Brown SA. The impact of adolescent binge drinking and sustained abstinence on affective state. Alcohol Clin Exp Res. 2013;37(8):1432-9.

11. Benedetto L, La Fauci E, Ingrassia M. Exploring meta-worry and perceived parenting behaviors in adolescents’ anxiety. Life Span and Disability. 2018;XXI(2):117–41.

12. Berenz EC, McNett S, Rappaport LM, Vujanovic AA, Viana AG, Dick D, et al. Age of alcohol use initiation and psychiatric symptoms among young adult trauma survivors. Addict Behav. 2019;88:150-6.

13. Bernard DL, Lige QM, Willis HA, Sosoo EE, Neblett EW. Impostor phenomenon and mental health: The influence of racial discrimination and gender. Journal of Counseling Psychology. 2017;64(2):155-66.

14. Bernusky HCR, Tibbo PG, Conrod PJ, Yunus FM, Keough MT, Thompson KD, et al. Do Anxiety Symptoms Mediate the Association Between Cannabis Use Frequency and Psychotic-Like Experiences in Emerging Adult Undergraduates? Can J Psychiatry. 2023;68(11):860-9.

15. Bieniak KH, Tinkle BT, Tran ST. The role of functional disability and social support in psychological outcomes for individuals with pediatric hypermobile ehlers-danlos syndrome. J Child Health Care. 2022:13674935221143822.

16. Bilali A, Katsiroumpa A, Koutelekos I, Dafogianni C, Gallos P, Moisoglou I, et al. Association Between TikTok Use and Anxiety, Depression, and Sleepiness Among Adolescents: A Cross-Sectional Study in Greece. Pediatric reports. 2025;17(2).

17. Bluth K, Campo RA, Futch WS, Gaylord SA. Age and Gender Differences in the Associations of Self-Compassion and Emotional Well-being in A Large Adolescent Sample. J Youth Adolesc. 2017;46(4):840-53.

18. Brolin Laftman S, Ostberg V, Wahlstrom J, Ramstedt M, Raninen J. Exposure to parental problem drinking during adolescence and symptoms of depression and anxiety in young adulthood: A Swedish national cohort study. Drug and alcohol review. 2024;43(6):1461-72.

19. Buckner JD, Thomas KL, Morris PE. Sexism and alcohol-related problems among women: The role of social anxiety and coping motivated drinking. The American Journal on Addictions. 2024;33(6):641-7.

20. Burke LA, Chijioke S, Le TP. Gendered racial microaggressions and emerging adult Black women's social and general anxiety: Distress intolerance and stress as mediators. Journal of Clinical Psychology. 2023;79(4):1051-69.

21. Cano MA, Schwartz SJ, MacKinnon DP, Keum BTH, Prado G, Marsiglia FF, et al. Exposure to ethnic discrimination in social media and symptoms of anxiety and depression among Hispanic emerging adults: Examining the moderating role of gender. Journal of Clinical Psychology. 2021;77(3):571-86.

22. Carcedo RJ, Fernandez-Rouco N, Fernandez-Fuertes AA, Martinez-Alvarez JL. Association between Sexual Satisfaction and Depression and Anxiety in Adolescents and Young Adults. Int J Environ Res Public Health. 2020;17(3).

23. Carlberg Rindestig F, Gillander Gådin K, Semb O, Dennhag I. Unwanted Online Sexual Solicitation Among Young People in a Swedish Psychiatric Sample: Occurrence and Associations with Depression and Anxiety. Journal of Child Sexual Abuse. 2024;33(5):589-607.

24. Carollo A, De Marzo S, Esposito G. Parental care and overprotection predict worry and anxiety symptoms in emerging adult students. Acta psychologica. 2024;248:104398.

25. Chen JS, Huang YT, Lin CY, Yen CF, Griffiths MD, Pakpour AH. Relationships of sexual orientation microaggression with anxiety and depression among lesbian, gay, and bisexual Taiwanese youth: Self-identity disturbance mediates but gender does not moderate the relationships. International Journal of Environmental Research and Public Health. 2021;18(24):12981.

26. Christiansen J, Qualter P, Friis K, Pedersen SS, Lund R, Andersen CM, et al. Associations of loneliness and social isolation with physical and mental health among adolescents and young adults. Perspect Public Health. 2021;141(4):226-36.

27. Davila M, Tubman JG. Gender, Maltreatment and Psychiatric Symptoms among Adolescents in Outpatient Substance Abuse Treatment. Child Adolesc Social Work J. 2020;37(4):385-96.

28. Davis JP, Pedersen ER, Tucker JS, Prindle J, Dunbar MS, Rodriguez A, et al. Directional associations between cannabis use and anxiety symptoms from late adolescence through young adulthood. Drug Alcohol Depend. 2022;241:109704.

29. De France K, Hancock GR, Stack DM, Serbin LA, Hollenstein T. The mental health implications of COVID-19 for adolescents: Follow-up of a four-wave longitudinal study during the pandemic. Am Psychol. 2022;77(1):85-99.

30. Di Blasi M, Cavani P, Pavia L, Lo Baido R, La Grutta S, Schimmenti A. The relationship between self-Image and social anxiety in adolescence. Child Adolesc Ment Health. 2015;20(2):74-80.

31. Duncan N, Zimmer-Gembeck MJ, Furman W. Sexual harassment and appearance-based peer victimization: Unique associations with emotional adjustment by gender and age. J Adolesc. 2019;75:12-21.

32. Durham EL, Micciche ET, Reimann GE, Archer C, Jeong HJ, Dupont RM, et al. Emotion regulation strategies as moderators of the relationship between negative life events and trait anxiety. Journal of affective disorders. 2025;370:26-33.

33. El-Sheikh M, Zeringue MM, Saini EK, Fuller-Rowell TE, Yip T. Discrimination and adjustment in adolescence: The moderating role of sleep. Sleep: Journal of Sleep and Sleep Disorders Research. 2022;45(1):1-15.

34. Ferro MA. Major depressive disorder, suicidal behaviour, bipolar disorder, and generalised anxiety disorder among emerging adults with and without chronic health conditions. Epidemiol Psychiatr Sci. 2016;25(5):462-74.

35. Fitzsimmons-Craft EE, Bardone-Cone AM. Examining Prospective Mediation Models of Body Surveillance, Trait Anxiety, and Body Dissatisfaction in African American and Caucasian College Women. Sex Roles. 2012;67(3-4):187-200.

36. Fontaine NMG, Brendgen M, Vitaro F, Boivin M, Tremblay RE, Côté SM. Longitudinal associations between delinquency, depression and anxiety symptoms in adolescence: Testing the moderating effect of sex and family socioeconomic status. Journal of Criminal Justice. 2019;62:58-65.

37. Fortier L, Castellanos-Ryan N, Chaput-Langlois S, Yale-Souliere G. Transactional associations between physical activity and depressive and anxious symptoms in adolescent girls and boys: Considering screen time and sleep duration. Research on Child and Adolescent Psychopathology. 2025:No-Specified.

38. Gasso AM, Mueller-Johnson K, Montiel I. Sexting, Online Sexual Victimization, and Psychopathology Correlates by Sex: Depression, Anxiety, and Global Psychopathology. Int J Environ Res Public Health. 2020;17(3).

39. Giannotta F, Nilsson KW, Aslund C, Olofdotter S, Vadlin S, Larm P. Anxiety, Sleep Problems, and Vigorous Physical Activity: Bidirectional Associations from Early Adolescence to Early Adulthood in Swedish Adolescents. Journal of youth and adolescence. 2024;53(6):1355-69.

40. Gillespie S, Winer JP, Issa O, Ellis BH. The role of discrimination, assimilation, and gender in the mental health of resettled Somali young adults: A longitudinal, moderated mediation analysis. Transcultural Psychiatry. 2023;60(1):74-85.

41. Gonzalez-Diez Z, Orue I, Calvete E. The role of emotional maltreatment and looming cognitive style in the development of social anxiety symptoms in late adolescents. Anxiety Stress Coping. 2017;30(1):26-38.

42. Grapin SL, Warner CM, Bixter MT, Cunningham DJ, Bonumwezi J, Mahmud F, et al. Online racial discrimination and mental health among Black undergraduates: The moderating role of gender. Journal of American College Health. 2024;72(1):310-8.

43. Hawes T, Zimmer-Gembeck MJ, Campbell SM. Unique associations of social media use and online appearance preoccupation with depression, anxiety, and appearance rejection sensitivity. Body Image. 2020;33:66-76.

44. Hellemans KGC, Wilcox J, Nino JN, Young M, McQuaid RJ. Cannabis Use, Anxiety, and Perceptions of Risk among Canadian Undergraduates: The Moderating Role of Gender. Canadian Journal of Addiction. 2019;10(3):22-9.

45. Herring MP, Gordon BR, McDowell CP, Quinn LM, Lyons M. Physical activity and analogue anxiety disorder symptoms and status: Mediating influence of social physique anxiety. J Affect Disord. 2021;282:511-6.

46. Johannessen EL, Andersson HW, Bjorngaard JH, Pape K. Anxiety and depression symptoms and alcohol use among adolescents - a cross sectional study of Norwegian secondary school students. BMC Public Health. 2017;17(1):494.

47. Kaltschik S, Pieh C, Dale R, Probst T, Pammer B, Humer E. Assessment of the Long-Term Mental Health Effects on Austrian Students after COVID-19 Restrictions. Int J Environ Res Public Health. 2022;19(20).

48. Kim Y, Schacter HL, Corner GW, Rasmussen HF, Margolin G. Does Family Context Moderate the Effects of Discrimination on Emerging Adults’ Health? Journal of Family Issues. 2021;42(12):2920-41.

49. Kornilaki EN. The psychological effect of COVID-19 quarantine on Greek young adults: Risk factors and the protective role of daily routine and altruism. Int J Psychol. 2022;57(1):33-42.

50. Kouros CD, Pruitt MM, Ekas NV, Kiriaki R, Sunderland M. Helicopter Parenting, Autonomy Support, and College Students' Mental Health and Well-being: The Moderating Role of Sex and Ethnicity. J Child Fam Stud. 2017;26:939-49.

51. Laftman SB, Grigorian K, Lundin A, Ostberg V, Raninen J. Bullying experiences before and after the transition from lower to upper secondary school: associations with subsequent mental health in a Swedish cohort. BMC Public Health. 2024;24(1):27.

52. Larsen SR, Rakesh D, Whittle S, Allen NB, Enticott PG, Mygind L. Residential greenness and adolescent mental health trajectories: A longitudinal pre-registered study. Environmental Research. 2025;283:122150.

53. Leadbeater BJ, Thompson K, Sukhawathanakul P. It gets better or does it? Peer victimization and internalizing problems in the transition to young adulthood. Dev Psychopathol. 2014;26(3):675-88.

54. Lee JI, Chang YP, Tsai CS, Yen CF. Internalized Sexual Stigma among Lesbian, Gay, and Bisexual Individuals in Taiwan: Its Related Factors and Association with Mental Health Problems. Int J Environ Res Public Health. 2022;19(4).

55. Lin C-Y, Strong C, Latner JD, Lin Y-C, Tsai M-C, Cheung P. Mediated effects of eating disturbances in the association of perceived weight stigma and emotional distress. Eating and Weight Disorders. 2020;25(2):509-18.

56. Maciel L, Basto-Pereira M. Child Sexual Abuse: the Detrimental Impact of its Specific Features. Child Indicators Research. 2020;13(6):2117-33.

57. Mar J, Larranaga I, Ibarrondo O, Gonzalez-Pinto A, Hayas CL, Fullaondo A, et al. Socioeconomic and gender inequalities in mental disorders among adolescents and young adults. Span J Psychiatry Ment Health. 2024;17(2):95-102.

58. Marshal MP, Dermody SS, Shultz ML, Sucato GS, Stepp SD, Chung T, et al. Mental health and substance use disparities among urban adolescent lesbian and bisexual girls. J Am Psychiatr Nurses Assoc. 2013;19(5):271-9.

59. Mayorga NA, Jardin C, Bakhshaie J, Garey L, Viana AG, Cardoso JB, et al. Acculturative stress, emotion regulation, and affective symptomology among Latino/a college students. J Couns Psychol. 2018;65(2):247-58.

60. McKinney C, Milone MC, Renk K. Parenting and late adolescent emotional adjustment: mediating effects of discipline and gender. Child Psychiatry Hum Dev. 2011;42(4):463-81.

61. Minhas M, Belisario K, Gonzalez-Roz A, Halladay J, Murphy JG, MacKillop J. COVID-19 impacts on drinking and mental health in emerging adults: Longitudinal changes and moderation by economic disruption and sex. Alcohol Clin Exp Res. 2021;45(7):1448-57.

62. Neblett EW, Jr., Bernard DL, Banks KH. The moderating roles of gender and socioeconomic status in the association between racial discrimination and psychological adjustment. Cognitive and Behavioral Practice. 2016;23(3):385-97.

63. Pachankis JE, Bernstein LB. An etiological model of anxiety in young gay men: From early stress to public self-consciousness. Psychology of Men & Masculinity. 2012;13(2):107-22.

64. Perkins T, Durkee M, Banks J, Ribero-Brown B. Gender and Racial Identity Moderate the Effects of Online and Offline Discrimination on Mental Health: Dismantling Systems of Racism and Oppression during Adolescence. Journal of research on adolescence : the official journal of the Society for Research on Adolescence. 2022;32(1):244-53.

65. Poteat VP, Scheer JR, DiGiovanni CD, Mereish EH. Short-term prospective effects of homophobic victimization on the mental health of heterosexual adolescents. J Youth Adolesc. 2014;43(8):1240-51.

66. Ranta K, Kaltiala-Heino R, Frojd S, Marttunen M. Peer victimization and social phobia: a follow-up study among adolescents. Soc Psychiatry Psychiatr Epidemiol. 2013;48(4):533-44.

67. Reed E, Salazar M, Behar AI, Agah N, Silverman JG, Minnis AM, et al. Cyber Sexual Harassment: Prevalence and association with substance use, poor mental health, and STI history among sexually active adolescent girls. J Adolesc. 2019;75:53-62.

68. Rieselbach MM, Corley RP, Hewitt JK, Rhee SH. Anxiety-specific associations with substance use: Evidence of a protective factor in adolescence and a risk factor in adulthood. Dev Psychopathol. 2023;35(3):1484-96.

69. Sares-Jaske L, Czimbalmos M, Majlander S, Siukola R, Klemetti R, Luopa P, et al. Gendered Differences in Experiences of Bullying and Mental Health Among Transgender and Cisgender Youth. J Youth Adolesc. 2023;52(8):1531-48.

70. Schiffrin HH, Erchull MJ, Sendrick E, Yost JC, Power V, Saldanha ER. The Effects of Maternal and Paternal Helicopter Parenting on the Self-determination and Well-being of Emerging Adults. Journal of Child and Family Studies. 2019;28(12):3346-59.

71. Šeboková G, Popelková M. Self-consciousness and internalizing problems in adolescence: Moderating effect of family variables. Studia Psychologica. 2016;58(2):105–21.

72. Shorey RC, Anderson S, Lookatch S, Moore TM, Stuart GL. The Relation Between Moment-to-Moment Mindful Attention and Anxiety Among Young Adults in Substance Use Treatment. Subst Abus. 2015;36(3):374-9.

73. Skogen JC, Hjetland GJ, Boe T, Hella RT, Knudsen AK. Through the Looking Glass of Social Media. Focus on Self-Presentation and Association with Mental Health and Quality of Life. A Cross-Sectional Survey-Based Study. Int J Environ Res Public Health. 2021;18(6).

74. Skogen JC, Sivertsen B, Lundervold AJ, Stormark KM, Jakobsen R, Hysing M. Alcohol and drug use among adolescents: and the co-occurrence of mental health problems. Ung@hordaland, a population-based study. BMJ Open. 2014;4(9):e005357.

75. Stearns M, McKinney C. Parent-child anxiety symptoms in emerging adults: Moderation by gender and religiosity. Journal of Family Issues. 2021;42(11):2691-710.

76. Surprenant R, Bezeau D, Tiraboschi GA, Garon-Carrier G, Cabot I, Brodeur M, et al. Associations between youth lifestyle habits, sociodemographic characteristics, and health status with positive mental health: A gender-based analysis in a sample of Canadian postsecondary students. Preventive medicine reports. 2025;51:103015.

77. Tao X. Exposure to gendered racial/ethnic discrimination, friendships, and mental health among young women of color. Dissertation Abstracts International Section A: Humanities and Social Sciences. 2024;85(12-A):No-Specified.

78. Thornhill CW, Castillo LG, Pina-Watson B, Manzo G, Cano MA. Mental health among Latinx emerging adults: Examining the role of familial accusations of assimilation and ethnic identity. J Clin Psychol. 2022;78(5):892-912.

79. van Beusekom G, Bos HMW, Overbeek G, Sandfort TGM. Same-sex attraction, gender nonconformity, and mental health: The protective role of parental acceptance. Psychology of Sexual Orientation and Gender Diversity. 2015;2(3):307-12.

80. Vannucci A, Flannery KM, Ohannessian CM. Social media use and anxiety in emerging adults. J Affect Disord. 2017;207:163-6.

81. Whitton SW, Godfrey LM, Crosby S, Newcomb ME. Romantic Involvement and Mental Health in Sexual and Gender Minority Emerging Adults Assigned Female at Birth. J Soc Pers Relat. 2020;37(4):1340-61.

82. Wijsbroek SA, Hale WW, 3rd, Raaijmakers QA, Meeus WH. The direction of effects between perceived parental behavioral control and psychological control and adolescents' self-reported GAD and SAD symptoms. Eur Child Adolesc Psychiatry. 2011;20(7):361-71.

83. Wise MW, Haraldsdottir K, Anderson S, Stiner Q, McGuine T. Race and Socioeconomic Status Influence the Benefits of Returning to Sports During COVID-19. Annual Meeting American Medical Society for Sports Medicine, AMSSM 2023; Phoenix, AZ United States: Clinical Journal of Sport Medicine 2023. p. 283-98.

84. Woodward E, Fitzcharles S, Coutellier L. The effects of prepubertal ovariectomy and chronic stress on activity of brain limbic regions in adult mice. Stress (Amsterdam, Netherlands). 2025;28(1):2514494.

85. Zvolensky MJ, Kauffman BY, Bogiaizian D, Viana AG, Bakhshaie J, Rogers AH, et al. Worry Among Latinx Young Adults: Relations to Pain Experience, Pain-Related Anxiety, and Perceived Health. J Racial Ethn Health Disparities. 2019;6(5):981-9.

86. Tu E-N, Manley H, Saunders KEA, Creswell C. Systematic Review and Meta-analysis: Risks of Anxiety Disorders in Offspring of Parents With Mood Disorders. Journal of the American Academy of Child & Adolescent Psychiatry. 2023.

87. Woodward MJ, McGettrick CR, Dick OG, Ali M, Teeters JB. Time Spent on Social Media and Associations with Mental Health in Young Adults: Examining TikTok, Twitter, Instagram, Facebook, Youtube, Snapchat, and Reddit. Journal of Technology in Behavioral Science. 2025.

88. Murad MH, Mustafa RA, Schünemann HJ, Sultan S, Santesso N. Rating the certainty in evidence in the absence of a single estimate of effect. Evid Based Med. 2017;22(3):85-7.
